# Novel Conversions of a Multifunctional, Bio-sourced Lactone Carboxylic Acid

**DOI:** 10.24820/ark.5550190.p012.081

**Published:** 2023-12-10

**Authors:** Trinadh Kaicharla, Sangjun Lee, Ruiqin Wang, Ashok D. Pehere, Shu Xu, Thomas R. Hoye

**Affiliations:** Department of Chemistry, 207 Pleasant St. SE, University of Minnesota, Minneapolis, MN 55455 USA

**Keywords:** Lactone-containing acrylates/methacrylates, bio-sourced monomers, scaffolds

## Abstract

The plant-derived compounds furfuryl alcohol and itaconic anhydride are known to undergo a Diels-Alder reaction at room temperature and in bulk to efficiently give an alkene-containing lactone carboxylic acid. Reported here is the conversion of this substance to a variety of derivatives via hydrogenation, epoxidation, or halolactonization reactions. Most notable is the formation of a set of three related acrylate or methacrylate esters (see graphical abstract) produced by direct acylative ring opening of ether bonds using Sc(OTf)_3_ and (meth)acrylic anhydride. These esters are viewed as promising candidates for use as biorenewable monomers in reversible addition-fragmentation chain transfer (RAFT) polymerization reactions.

## Graphical Abstract

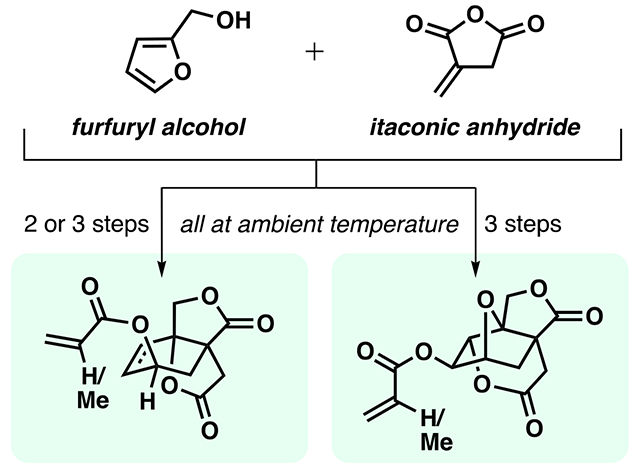

## Introduction

Professor Samir Zard’s impressive pioneering advances in and contributions to numerous aspects of radical chemistry (among other subjects) are renowned. Perhaps at the top of the Zard team’s accomplishments are his and his coworkers’ insightful contributions^1,2^ to the underpinnings^3,4^ of reversible addition-fragmentation chain transfer (RAFT) polymerization. By now, many classes of polymers can be produced using this extremely important and practical technology.^5–7^ Among the first examples of very effective RAFT monomers^1–4^ were acrylate and methacrylate esters, a class of monomers still widely used.

**Figure 1.**
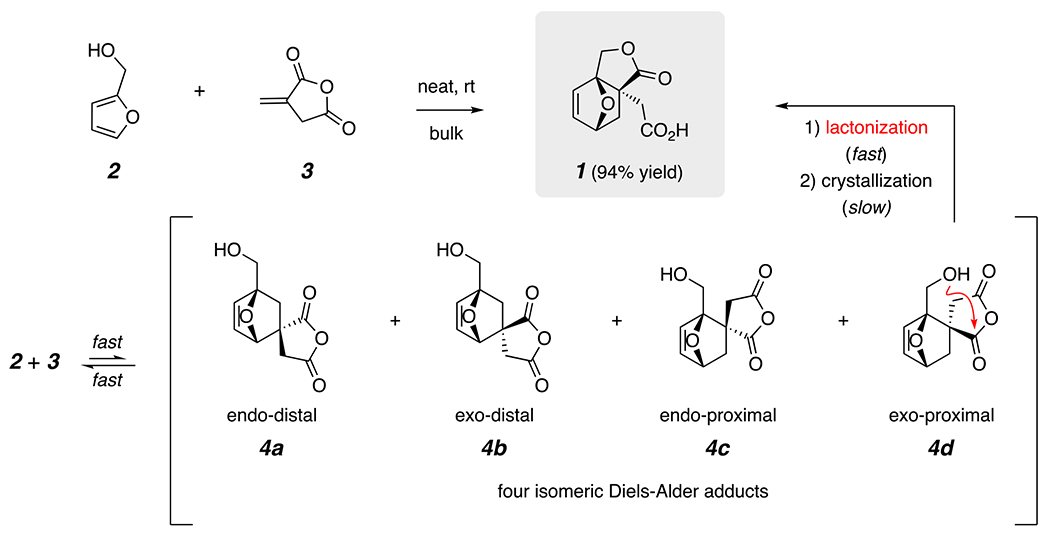
The known Diels-Alder reaction of a neat mixture of itaconic anhydride (**3**) and furfuryl alcohol (**2**) at room temperature^12–14^ proceeds by reversible formation of the four isomeric anhydrides **4a-d**, one of which selectively lactonizes to produce the crystalline lactone acid **1**.^12,13^

Efforts to produce useful polymers derived from monomers that can be readily accessed from, plant-based (ergo, renewable) substances abound.^8–11^ With an eye toward developing an efficient method for achieving novel bio-sourced monomers, we previously reported a method for the efficient production of the lactone acid **1** (Figure 1).^12,13^ Researchers at York University, carrying out concurrent independent studies, made a similar discovery.^14^ The process involves merely mixing furfuryl alcohol (**2**; sourced, e.g., from hemicelluloses) with itaconic anhydride (**3**; sourced, e.g., from starches) in the absence of solvent at ambient temperature. We determined that the metastable intermediates produced by a number of competing, yet reversible, Diels-Alder (DA) cycloadditions (giving the four isomeric 1:1 DA adducts **4**) were driven thermodynamically to the predominant product **1** in 94% yield by its selective intramolecular anhydride ring-opening and crystallization within the bulk mixture. The strained alkene in **1**, as well as in some of its derived esters and amides, has been shown to be a monomer amenable to ring-opening metathesis polymerization (ROMP).^12,14–16^

## Results and Discussion

**Figure 2.**
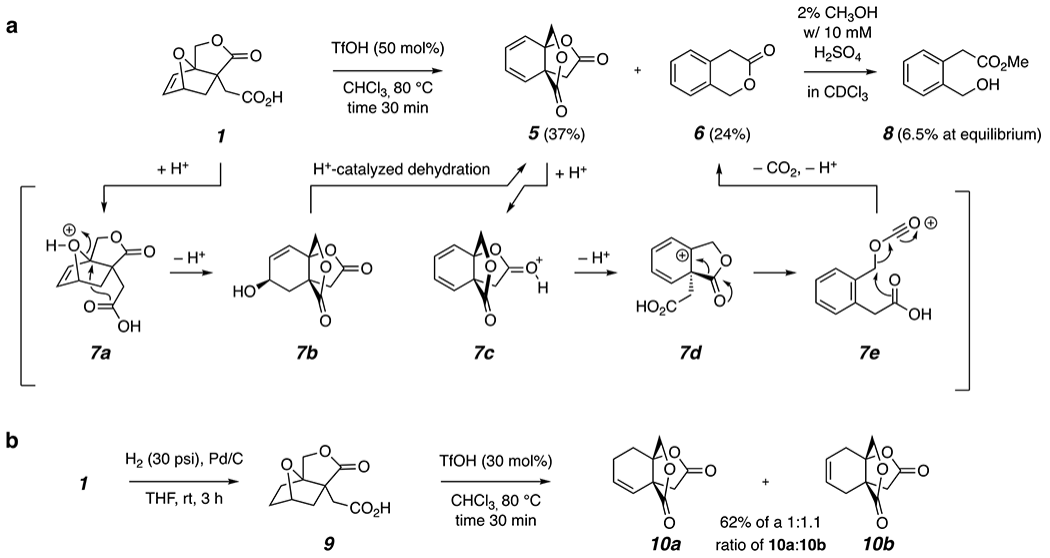
**(a)** Triflic acid-catalyzed conversion of **1** to a mixture of the dilactone-diene **5** and isochromanone **6** via the proposed sequence proceeding through intermediates **7a-e**. **(b)** Triflic acid-catalyzed conversion of the dihydro derivative of **1**, the saturated lactone acid **9**, leads to a mixture of the two monoenes **10a** and **10b**.

We first explored Bronsted acid-catalyzed reactions of **1** (Figure 2a). Treatment with 50 mol% of triflic acid in refluxing chloroform for 30 minutes gave rise to a mixture of the dilactone **5** and isochromanone (**6**). This reaction was always accompanied by formation of a considerable amount of a dark-colored resinous substance suggestive of highly conjugated oligomeric material. In contrast, purified **5** could be further (and quite cleanly) converted into **6** under the same conditions with very little darkening of the reaction mixture. Generation of **5** can be rationalized in straightforward fashion by the acid-catalyzed ring-opening of the strained allylic ether in **1** (cf. **7a**) followed by dehydration of the resulting allylic alcohol **7b**. The further conversion of dilactone **5** to **6** is more unusual; the elements of CO_2_ are lost in the process. This can be rationalized by the acid-catalyzed opening of the protonated lactone **7c** to the pentadienyl cation **7d**, which can then fragment to the acylium ion **7e** in an event driven by the aromatization of the benzene ring. Loss of carbon dioxide and the proton furnishes isochromanone (**6**).

We attempted, unsuccessfully, to achieve ring-opening transesterification polymerization (ROTEP) of **6**. This is consistent with the thermodynamic reluctance of **6** to undergo methanolysis in 2 vol% methanolic chloroform, a proxy we recently reported for evaluating the suitability of any lactone to function as a ROTEP monomer.^17^ Only 6.5% of the methyl ester **8** was formed at equilibrium; for comparison, γ-butyrolactone (another reluctant ROTEP monomer) and δ-valerolactone (a competent ROTEP monomer) produced their corresponding methyl esters to the extent of 18.7% and 86.8%, respectively, in the same assay.^17^ (Notably, radical ring-opening polymerization of the thionolactone analog of **6** was recently reported.^18^)

The alkene in **1** could be smoothly hydrogenated to the saturated derivative **9** (Figure 2b); no evidence of hydrogenolysis of an allylic C–O bond was seen. Triflic acid treatment of **9** also led to formation of a dilactone followed by dehydration to produce the isomeric alkenes **10a** and **10b** in an equilibrium ratio of ca. 1:1. As anticipated, the rate of the ring-opening reaction of the saturated bicyclic ether **9** was considerably slower (in situ ^1^H NMR) than that of the allylic analog **1**.

**Figure 3.**
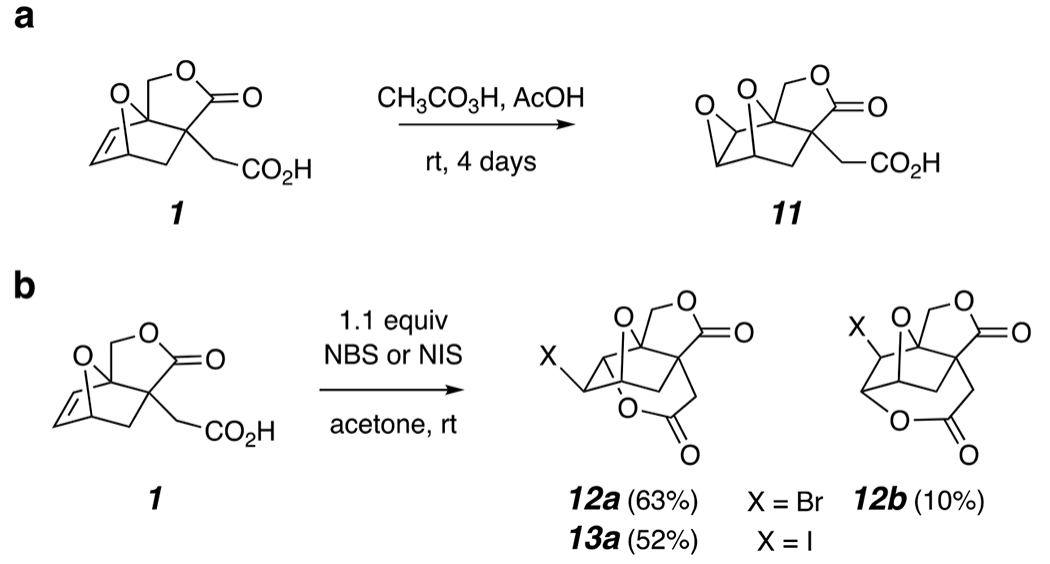
**(a)** Epoxidation of **1** with peracetic acid at room temperature. **(b)** Halolactonizations of **1** with NBS or NIS in acetone at room temperature.

We next explored reactions of the alkene **1** with electrophilic reagents (Figure 3). Predictably, epoxidation using peracetic acid led to efficient formation of the exo-epoxide **11**. We initially used in situ generated performic acid at 60 °C^19^ to effect this epoxidation, but on one occasion we observed a strong exotherm. We do not recommend use of this unstable reagent^20^ if alternative expoxidation reagents will suffice.

We also carried out the halolactonization reactions of the alkene acid **1** using NBS or NIS in acetone. In the first case, the major product was the bromovalerolactone derivative **12a**, accompanied by a small amount of the (largely coeluting) seven-membered lactone **12b**. These were present in a 6:1 ratio in the crude product mixture (^1^H NMR). Use of NIS gave **13a** the iodo analog of **12a**; the seven-membered isomer was not definitively identified.

With an eye toward converting this core skeleton into a(n) (meth)acrylate ester, we wondered whether some of the carboxylic acids **1**, **9**, or **11** could be transformed by an acid-catalyzed process into an isomeric dilactone alcohol by intramolecular ring-opening of a suitably reactive, cyclic, C–O ether bond by the pendant carboxylic acid (cf. **1** to **7b** or the analogous ring-opening of **9** enroute to **10a/b**). The hydroxyl group in the alcohol product would then be available for (meth)acrylation.

**Figure 4.**
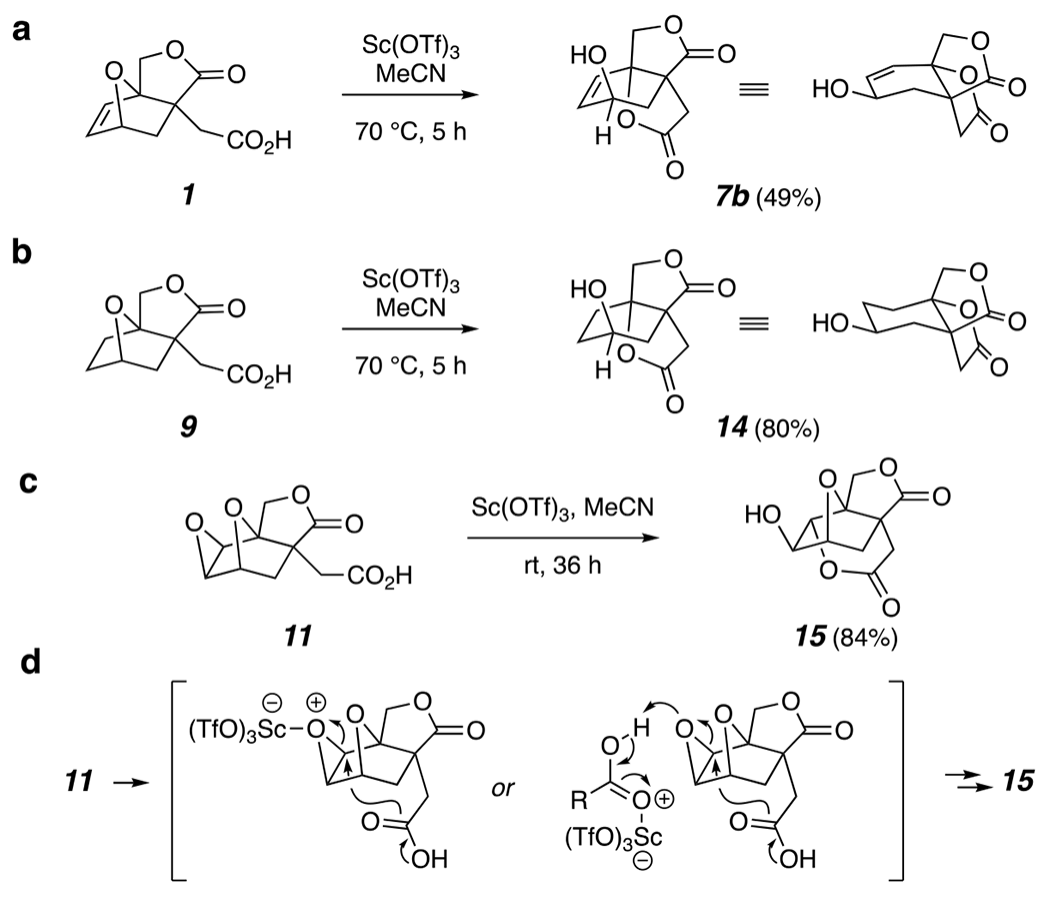
Scandium triflate catalyzed ring-opening to dilactone alcohols via cleavage of strained cyclic ether bonds in **(a)** the alkene **1**, **(b)** the alkane **9**, and **(c)** the epoxide **11**. **(d)** Depiction of two possibilities for the key step in a likely mechanism for the Sc(III)-catalyzed conversion of, for example, **11** to **15**.

For **1** it was necessary, of course, to identify conditions milder than those described in Figure 1a in which the now-desired intermediate **7b** was further transformed. We found that scandium triflate served as a very good catalyst for this purpose. Treating **1** with 10 mol% Sc(OTf)_3_ in acetonitrile, which provided a homogenous reaction mixture from the outset, at 70 °C for 5 hours provided the product **7b** in 49% yield. The saturated analog **9** could be rearranged under the same conditions to give the dilactone alcohol **14** in 80% yield (Figure 4a). The more labile epoxide substrate **11** was quickly opened to the six-membered lactone alcohol **15** at room temperature (Figure 4c). The use of BF3•OEt_2_ resulted in an essentially identical yield of **18c**, but other than that we did perform any further screening of catalysts. All of these lactonizations can be rationalized by acid activation of the ether bond with concomitant participation by the carboxylic acid. This could be achieved by direct activation of the ether oxygen by the Lewis acidic Sc(III) species or by the proton from a Lewis acid-activated Bronsted acid [RO(H)•Sc(III)], as depicted in the intermediate structures in Figure 4d.

**Figure 5.**
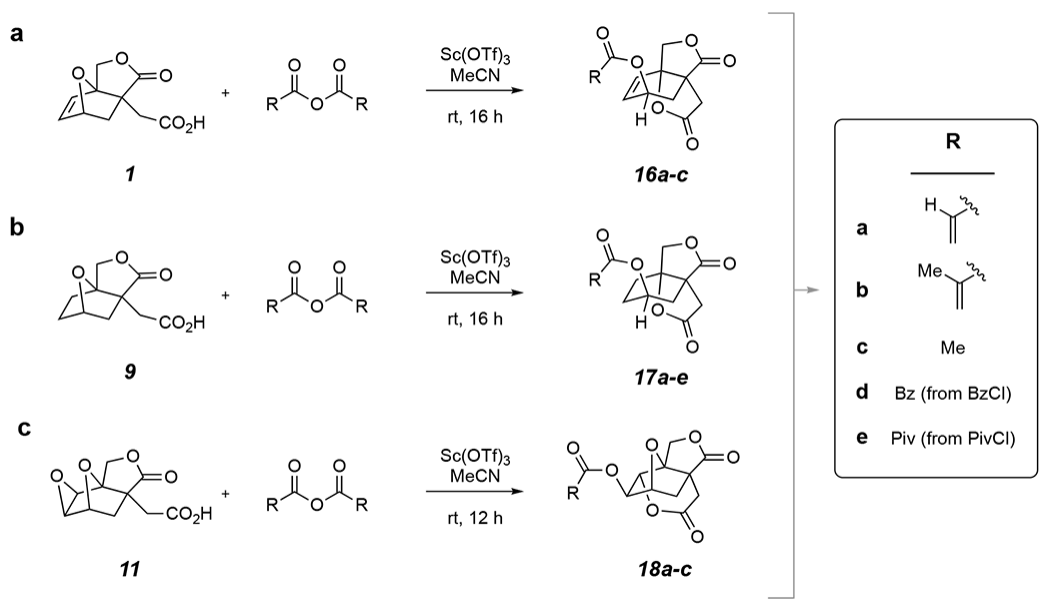
Acylative ether ring-opening directly to esters from: **(a)** alkene **1**, **(b)** alkane **9**, and **(c)** epoxide **11**.

Although we presumed it would be a straightforward matter to esterify these three alcohols with methacrylic or acrylic anhydride or chloride, these experiments were rendered unnecessary because of the reaction shown in Figure 5a. Namely, a mixture of the alkene acid **1** and methacrylic anhydride in acetonitrile was treated with, again, 10 mol% Sc(OTf)_3_. Pleasingly, the methacrylate ester **16b** was smoothly formed in this telescoped, one-pot, net acylative ring-opening reaction. Not surprisingly, acrylic anhydride proceeded analogously to give the acrylate ester **16a**. Furthermore, both of the substrates **9** and **11** underwent analogous processes, leading to **17a** or **17b** and **18a** or **18b**. Not surprisingly, acetate esters (from Ac_2_O) were directly produced from all three of the ethers **1**, **9**, and **11**. Finally, the benzoate (from BzCl) and pivalate esters (from PivCl) esters **17d** and **17e** were smoothly formed from the alkane acid **9** (the only ether we examined).

Finally, we addressed the question of whether the alcohol products in Figure 4 were intermediates in the formation of the esters in the reactions shown in Figure 5. This meant evaluating the relative rates of the two different classes of ring-opening: namely, the isomerization to the alcohol (*k*_alcohol_) vs. the acylative opening to give the esters (*k_ester_*). We did this for several of the reactions using in situ NMR monitoring. A solution of the ether-containing substrate in CD_3_CN was split into two portions into separate NMR sample tubes. The anhydride acylating agent was added to one of the two. Finally, an equal volume of a stock solution of Sc(OTf)_3_ in CD_3_CN was added to each tube. Using this protocol for reaction mixture preparation ensured that both of the reaction mixtures were subjected to essentially the same conditions (e.g., trace water in the acetonitrile). Reaction progress was then periodically monitored over time by in situ NMR spectroscopy.

**Figure 6.**
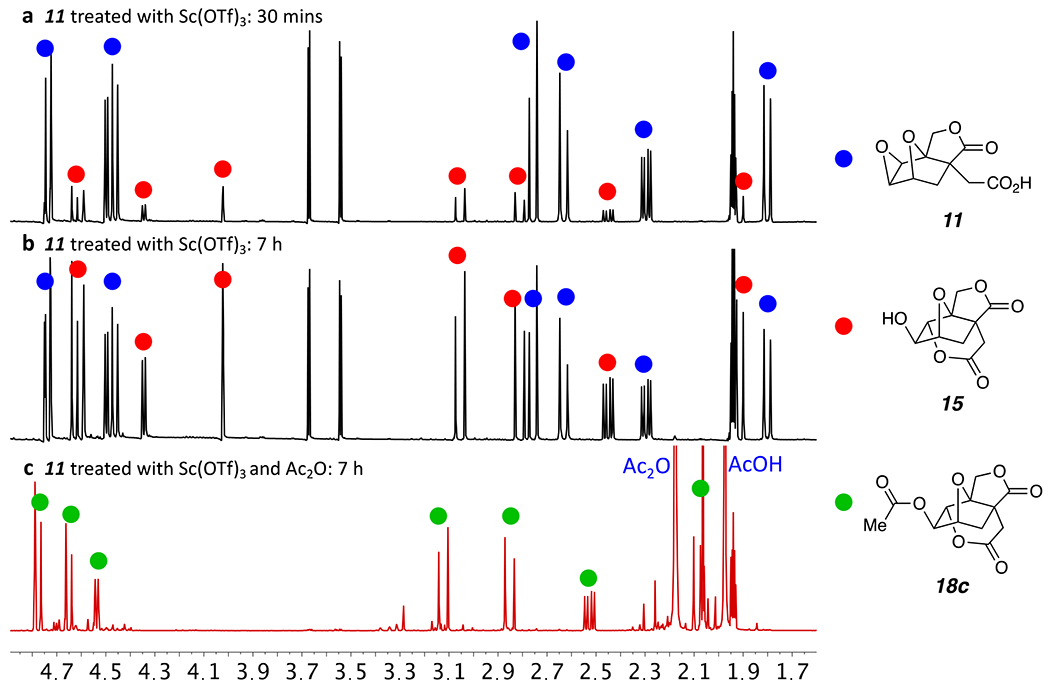
^1^H NMR spectra for reactions of epoxide **11** with Sc(OTf)_3_ (10 mol%) in CD_3_CN at room temperature with no added Ac_2_O after **(a)** 30 minutes and **(b**) 7 hours or **(c**) with added Ac_2_O (2 equiv) after 7 hours.

One example of (a portion of) the data collected from this kind of study is shown in Figure 6. This is for the reaction of the epoxide acid **11** without and with acetic anhydride (2 equiv) to give either the alcohol **15** or the acetate ester **18c**. After 30 minutes (panel a) and 7 hours (panel b) at ambient temperature, in the absence of the anhydride the reaction had proceeded to the alcohol **15** to the extent of 16% and 50% conversions, respectively. In contrast, in the presence of the Ac_2_O, after only 30 minutes the reaction had proceeded to form the acetate ester **18c** to 85% conversion (not shown) and after 7 h >99% conversion (panel c, the observable minor resonances are attributable to a mixed anhydride of **11**). These types of experiments strongly suggest that the electrophilic Sc(III)-activated anhydride is more effective at promoting the ring opening compared to Sc(III) alone. Similar observations were made with the substrates **1** and **9** as well.

## Conclusions

A variety of transformations of the readily available lactone acid **1** are reported. These lead to interesting scaffolds with different arrays of functional groups. Most notably, the acrylate or methacrylate esters **16a** and **16b** are available in two steps from furfuryl alcohol and itaconic anhydride. The (meth)acrylates **17a/b** and **18a/b** are efficiently available in three steps from the same commodity chemicals. We are now beginning studies to explore RAFT polymerizations of these (meth)acrylate monomers.

## Experimental Section

### General Experimental Protocols:

**Nuclear magnetic resonance (NMR) spectra** (^1^H and ^13^C) were recorded on a Bruker HD-500 spectrometer. ^1^H chemical shifts in CDCl_3_ samples are referenced to TMS (δ 0.00), in acetone-*d_6_* samples to residual solvent protons at 2.05, DMSO-*d_6_* to residual solvent protons at 2.50, and in CD_3_CN to residual solvent protons at 1.94. Data are reported using the following format: chemical shift (ppm) [multiplicity, coupling constant(s) (in Hz), integral (to the nearest whole integer), and assignment of the proton]. Coupling constant values have been deduced using protocols previously described.^21,22^ Non-first order multiplets in a ^1^H NMR spectrum are designated by ‘nfom’. Non-first order doublets in a ^1^H NMR spectrum (e.g., present in a 1,4-disubstitutedbenzene ring) are designated by ‘nfod’ and the apparent doublet coupling constant (actually, e.g., *J*_*2,3* +_ *J_2,3’_*) is indicated as *J_app_*. ^13^C{^1^H} NMR chemical shifts are referenced to the carbon atom in CDCl_3_ to 77.16 ppm, in acetone-*d_6_* to 29.8 ppm, and in DMSO-*d_6_* to 39.5 ppm. Where assigned, carbon chemical shifts were deduced from analysis of HSQC and/or HMBC data.

**Infrared (IR) spectral data** were collected on a Bruker spectrometer (model Alpha II). Samples were deposited as films on a diamond window (solids by evaporation from DCM; liquids by direct application) in the mode of attenuated total reflectance (ATR). Peaks are reported in cm^−1^.

**High-resolution mass spectrometry** (HRMS) measurements were made using ESI ionization with a Thermo instrument (model Orbitrap Velos, which has a mass accuracy of ≤3). An external calibrant (Pierce^™^ LTQ) was used. Samples were injected directly into the ion source.

**Medium pressure liquid chromatography** (MPLC) was used to purify products. Silica gel (normal-phase, 20-40 μm, 60 Å pore size, Teledyne RediSep Rf Gold^®^) columns were hand-packed into Michel-Miller^®^ glass columns. The equipment used consisted of a Waters HPLC pump (model 510) and a Waters (R401) differential refractive index detector. Preparative flash chromatography was done on silica gel (230-400 mesh) columns. Thin layer chromatography (TLC) was performed on silica-gel-coated, plastic-backed plates (Machery-Nagel) that were visualized by staining with KMnO_4_ solution.

**Heating** of reactions performed above ambient temperature was done in silicone oil baths that had been preequilibrated to the desired temperature prior to immersion of the reaction vessel.

#### (±)-3a,7a-(Methanooxymethano)benzofuran-2,10(3*H*)-dione (5) and 3-Isochromanone (6).

The lactone acid **1** (200 mg, 0.95 mmol) was suspended in chloroform (10 mL) in a 20 mL Schlenk flask. Trifluoromethanesulfonic acid (TfOH, 40 μL, 0.45 mmol) was added to the mixture. A brown color was immediately observed and this mixture was stirred at 80 °C for 30 min. The color of the solution turned dark brown and formation of a black precipitate was observed. The chloroform supernatant was filtered through short pad of silica that was eluted with 20 mL of additional chloroform. The obtained chloroform solution was concentrated in vacuo and the residue was purified by MPLC (7:3 hexanes:EtOAc elution) to give the rearranged dilactone **5** (68 mg, 0.38 mmol, 37%) as a white crystalline solid along with 3-isochromanone **6** (34 mg, 0.38 mmol, 24%), also as a white crystalline solid. Data for the dilactone (**5**): ^1^H NMR (500 MHz, Chloroform-*d*) δ 6.23–6.07 (nfom, 2*H*), 6.03–5.87 (nfom, 2*H*), 4.75 (dd, *J* = 10.8, 0.7 Hz, 1H, C8*H_a_*H_b_), 4.25 (d, *J* = 10.9 Hz, 1H, C8H_a_*H_b_*), 3.16 (dd, *J* = 18.1, 0.7 Hz, 1H, C3H_a_*H_b_*), and 2.75 (d, *J* = 18.1 Hz, 1H, C3*H_a_*H_b_). ^13^C{^1^H} NMR (126 MHz, Chloroform-*d*) δ 176.5, 171.7, 125.2, 123.9, 123.3, 122.9, 87.5, 76.1, 51.0, and 38.6. IR (neat): 1777, 1250, and 736 cm^−1^. HRMS (ESI) *m/z*: [M + Na]^+^ Calcd for C_10_H_8_NaO_4_^+^ for 215.0320; Found 215.0308. mp: 105–107 °C. 3-Isochromanone (**6**) (NMR data matches well with literature values^23^): ^1^H NMR (500 MHz, Chloroform-*d*) δ 7.36–7.29 (m, 2H), 7.26–7.21 (m, 2*H*), 5.31 (s, 2H, C*H*_2_O), and 3.71 (s, 2H, C*H_2_*CO). ^13^C{^1^H} NMR (126 MHz, Chloroform-*d*) δ 170.8, 131.7, 131.1, 128.9, 127.5, 127.2, 124.8, 70.2 and 36.3. IR (neat): 1746, 1408, 1250, 1222, 1030, and 775 cm^−1^. mp: 80–82 °C (lit.^24^ 80–81 °C).

#### (±)-2-((3a*R*,6*S*)-1-Oxotetrahydro-3*H*-3a,6-epoxyisobenzofuran-7a(1*H*)-yl)acetic acid (9).

The lactone acid **1** (4.9 g, 23.3 mmol) and Pd/C (10%, 350 mg) were placed in a 250 mL Fisher-Porter tube with a stirring bar. Under a nitrogen atmosphere, THF (100 mL) was added and the reaction vessel was purged with H_2_. The reactor headspace was pressurized to ca. 10 psi with H_2_ and then the pressure was slowly released. This cycle was repeated twice more. The reactor was then pressurized to 30 psi of H_2_ and the mixture was stirred for 3 h at room temperature. The crude reaction mixture was filtered through a small plug of silica (eluant: 10 mL THF). The volatiles were removed under reduced pressure to provide a white solid, which was dried under vacuum to give the lactone acid **9** (4.8 g, 97%) as a white crystalline solid. ^1^H NMR (500 MHz, Acetone-*d_6_*) δ 4.59 (dd, *J =* 10.3 Hz, 1H, C_3_*Ha*Hb), 4.51 (dd, *J =* 5.3, 5.3 Hz, 1H, *H6*), 4.41 (d, *J =* 10.3 Hz, 1H, C3H_a_*H_b_*), 2.85 (d, *J =* 15.6 Hz, 1H, *H_a_*H_b_), 2.60 (d, *J =* 15.6 Hz, 1H, C8H_a_*H_b_*), 2.22 (ddd, *J =* 12.4, 5.0, 2.4 Hz, 1H, C7H_endo_*H_exo_*), 2.09–2.01 (m, 1H), 1.89 (dddd, *J* = 12.3, 9.2, 5.6, 2.4 Hz, 1H), 1.82 (d, *J =* 12.4 Hz, 1H, C7*H_endo_*H_exo_), and 1.77–1.66 (m, 2H). ^13^C{^1^H} NMR (126 MHz, Acetone-*d*_6_) δ 179.7, 172.1, 92.8, 76.9, 69.5, 53.8, 45.6, 39.9, 29.5, and 25.1. IR (neat): 2978, 1767, 1732, 1134, and 1005 cm^−1^. HRMS (ESI) *m/z*: [M + Na]^+^ Calcd for C_10_H_12_NaO_5_^+^ for 235.0582; Found 235.0566. mp: 16 3–164 °C.

#### (±)-4,7-Dihydro-3a,7a-(methanooxymethano)benzofuran-2,10(3*H*)-dione (10b) and (±)-6,7-Dihydro-3a,7a-(methanooxymethano)benzofuran-2,10(3*H*)-dione (10a).

The lactone acid **9** (42 mg, 0.2 mmol) was suspended in chloroform (2 mL) in a 5 mL Schlenk flask. Trifluoromethanesulfonic acid (TfOH, 6 μL, 30 mol%) was added. A pale brown color was immediately observed. This solution was stirred for 2 h at 80 °C. The chloroform was concentrated in vacuo; a ^1^H NMR spectrum indicated a nearly 1:1 mixture of dilactone products. The residue was purified and partially separated by flash column chromatography on SiO_2_ (2:1 hexanes:EtOAc elution) to give the dilactones **10b** (7 mg) as the faster eluting and **10a** (17 mg) as the slower eluting components (24 mg, 62% total yield), each as a colorless oil. NMR Data for **10a**: ^1^H NMR (500 MHz, Chloroform-*d*) δ 6.06 (ddd, *J* = 9.8, 5.7, 2.2 Hz, 1H, *H5*), 5.80 (ddd, *J* = 9.9, 2.8, 1.4 Hz, 1H, *H4*), 4.53 (d, *J* = 11.2, 1H, C8*H_a_*H_b_), 4.32 (d, *J* = 11.1 Hz, 1H, C8*H_a_*H_b_), 3.21 (d, *J* = 18.2, 1H, C3*H_a_*H_b_), 2.68 (d, *J* = 18.3 Hz, 1H, C3H_a_*H_b_*), 2.40 (ddddd, *J* = 18.7, 5.9, 5.9, 2.0, 1.5 Hz, 1H, C6H_a_*H_b_*), 2.27 (ddd, *J* = 14.0, 5.8, 1.9, 1H, C7*H_a_*H_b_), 2.19–2.11 (ddddd, *J* = 18.7, 11.8, 5.8, 2.4, 2.4 Hz 1H, C6H_a_*H_b_*), and 1.79 (ddd, *J* = 13.8, 11.9, 6.2 Hz, 1H, C7H_a_*H_b_*). ^13^C{^1^H} NMR (126 MHz, Chloroform-*d*) δ 176.0, 172.2, 130.2, 122.5, 87.6, 71.4, 49.9, 38.4, 26.2, and 22.8. NMR Data for **10b**: ^1^H NMR (500 MHz, Chloroform-*d*) δ 5.97–5.91 (nfom, 1H, *H5* or *H6*), 5.91–5.87 (nfom, 1H, *H5* or *H6*), 4.59 (d, *J* = 10.9 Hz, 1H, C8*H_a_*H_b_), 4.20 (d, *J* = 10.8 Hz, 1H, C8H_a_*H_b_*), 3.10 (d, *J* = 18.3 Hz, 1H, C3*H_a_*H_b_), 2.74 (d, *J* = 18.4 Hz, 1H, C3H_a_*H_b_*), 2.68–2.56 (m, 2H), 2.50 (m, 2H). ^13^C{^1^H} NMR (126 MHz, Chloroform-*d*) δ 178.7, 172.4, 125.6, 124.8, 88.0, 75.0, 48.1, 38.2, 31.0, and 29.8. Data from the mixture: IR (neat): 2924, 2853, 1770, 1191,1041, and 983 cm^−1^. HRMS (ESI) *m/z*: [M + H]^+^ Calcd for C_10_H_11_O_4_^+^ for 195.0657; Found 195.0645.

#### (±)-2-((1a*R*,2*R*,6a*R*,6b*R*)-4-Oxotetrahydro-6*H*-2,6a-epoxyoxireno[2,3-*e*]isobenzofuran-3a(4*H*)-yl)acetic acid (11).

The lactone acid **1** (2.28 g) was added to a 100 mL round-bottom flask. To this was added 30% H_2_O_2_ (10 mL) and formic acid (36 mL). The resulting homogenous mixture was place into a preheated (60 °C) oil bath for 2 h. The resulting colorless solution was evaporated under vacuum to obtain a white, sticky, solid material. This solid was recrystallized from ethanol (10 mL) to provide the epoxide of **11** (2.1 g, 86%) as a white crystalline solid. *Caution:* On one occasion, a large exotherm was observed following this similar procedure.^20^ We do not recommend using this procedure involving the *in situ* generation of the unstable performic acid. Therefore, we developed the following protocol. A *preferred procedure for this epoxidation* reaction using peroxyacetic acid: Lactone acid **1** (210 mg, 1 mmol) was added to a 20 mL glass vial. To this was added CH_3_CO_3_H in AcOH (2 mL, 32% peracid, ca. 8 mmol). The resulting homogenous mixture was stirred for 4 days at ambient temperature. To this colorless solution was added Et_2_O (^~^4 mL) followed by hexanes (^~^8 mL). A white precipitate appeared. The supernatant liquid phase was decanted the precipitate was washed with a small amount of Et_2_O. This solid was recrystallized from ethanol (2 mL) to provide the epoxide of **11** (185 mg, 82%) as a white crystalline solid. ^1^H NMR (500 MHz, DMSO-*d*6): δ 12.64 (s, 1H, -CO_2_*H*), 4.80 (d, *J* = 10.8 Hz, 1H, *H6*), 4.54 (d, *J* = 5.2 Hz, 1H, *H2*), 4.48 (d, *J* = 10.8 Hz, 1H, *H6’*), 3.78 (d, *J* = 3.3 Hz, 1H, *H1* or *H7*), 3.67 (d, *J* = 3.3 Hz, 1H, *H1* or *H7*), 2.68 (d, *J* = 15.3 Hz, 1H, C*H2*CO_2_H), 2.63 (d, *J* = 15.3 Hz, 1H, C*H2*CO_2_H), 2.16 (dd, *J* = 12.9, 5.2 Hz, 1H, *H3_exo_*), and 1.83 (d, *J* = 12.9 Hz, 1H, *H3_endo_*). ^13^C{^1^H} NMR (126 MHz, DMSO-*d_6_*) δ 177.1, 171.0, 88.4, 74.6, 67.4, 54.6, 48.6, 46.1, 38.5, and 36.0. IR (neat): 3083 (br), 1769, 1730, 1004, and 862 cm^−1^. HRMS (ESI) *m/z:* [M + H]^+^ Calcd for C_10_H_11_O_6_^+^ for 227.0556; Found 227.0543. mp: 158–159 °C.

#### (±)-3-Bromodihydro-2*H*,7*H*,9*H*-2,6a-methanodifuro[3,2-b:3’,4’-c]pyran-5,7(6*H*)-dione (12a) and (±)-10-Bromodihydro-7*H*,9*H*-2,6a:3,9a-dimethanofuro[3,4-*e*][1,4]dioxocine-5,7(6*H*)-dione (12b).

The lactone acid **1** (210 mg, 1.0 mmol) was dissolved in acetone (5 mL) in a 20 mL Schlenk flask. *N*-Bromosuccinimide (196 mg, 1.1 mmol) was added to the mixture stirred at room temperature for overnight. The reaction mixture was concentrated in vacuo; a proton NMR spectrum of this crude product mixture indicated the presence of an ca. 6:1 mixture of **12a** to **12b**. The residue was purified by MPLC (6:4 hexanes:EtOAc elution) to give a mixture of these largely coeluting bromolactones **12a** and **12b** (210 mg, 73%) as a broad melting white crystalline solid. NMR Data for the major bromolactone **12a** (extracted from the spectrum of the mixture). ^1^H NMR (500 MHz, Chloroform-*d*) δ 5.14 (dd, *J* = 1.4, 0.8 Hz, 1H, *H3a*), 4.80 (d, *J* = 11.4 Hz, 1H, *H9*), 4.70 (dd, *J* = 5.5, 0.8 Hz, 1H, *H2*), 4.64 (d, *J* = 11.6 Hz, 1H, *H9’*), 4.03 (d, *J* = 1.6 Hz, 1H, *H3*), 3.18 (d, *J* = 18.8 Hz, 1H, *H6*), 2.83 (dd, *J* = 13.9, 5.7 Hz, 1H, *H10_exo_*), 2.73 (d, *J* = 18.7 Hz, 1H, *H6’*), and 1.96 (d, *J* = 13.9 Hz, 1H, *H10_endo_*). ^13^C{^1^H} NMR (126 MHz, Chloroform-*d*) δ 175.0 (C7), 163.7 (C5), 85.9 (C3a), 84.9 (9 a), 83.7 (C2), 65.7 (C9), 52.2 (C3), 48.6 (C6a), 40.3 (C10), and 32.6 (C6). NMR Data for minor bromolactone **12b** (extracted from the spectrum of the mixture): ^1^H NMR (500 MHz, Chloroform-*d*) δ 5.05 (nfom, 1H, *H3*), 4.64 (d, *J* = 11.9 Hz, 1H, H9), 4.55 (s, 1H, H10), 4.47 (d, *J* = 11.9 Hz, 1H, H9’), 3.41 (d, *J* = 18.7 Hz, 1H, *H6*), 3.03 (ddd, *J* = 18.6, 2.3, 1.0 Hz, 1H, *H6’*), 2.45 (dddd, *J* = 12.7, 2.4, 2.4, 1.5 Hz, 1H, *H11_exo_*), and 2.34 (dd, *J* = 12.7, 1.1 Hz, 1H, *H11_endo_*). The resonance for H2 could not be definitively identified. Data for the mixture: IR (neat): 3010, 1778, 1750, 1707, 1375, 1190, 1062, 1009, and 585 cm^−1^. HRMS (ESI) *m/z:* [M + H]^+^ Calcd for C_10_H_10_BrO_5_^+^ for 288.9712; Found 288.9697. mp: 188–194 °C.

#### (±)-3-Iododihydro-2*H*,7*H*,9*H*-2,6a-methanodifuro[3,2-b:3’,4’-c]pyran-5,7(6*H*)-dione (13a).

The lactone acid **1** (42 mg, 0.20 mmol) *N*-iodosuccinimide (44 mg, 0.22 mmol) were combined in a NMR tube and acetone-*d*_6_ (0.6 mL) was added. The pale brown homogenous mixture was allowed to stand at room temperature. After 4 days NMR analysis indicated ^~^40% of unreacted starting acid along with unreacted NIS. The reaction mixture was concentrated in vacuo and the residue was purified by MPLC (6:4 hexanes:EtOAc elution) to give a the iodolactone **13a** (35 mg, 52%) as a white crystalline solid. This sample contained ca. 4% of a contaminant with many analogous resonances in the ^1^H NMR spectrum, although its structure could not be definitively identified as that of an isomeric iodolactone (cf. **12b** vs. **12a**). NMR Data for iodolactone **13a**: ^1^H NMR (500 MHz, Chloroform-*d*) δ 5.25 (dd, *J* = 2.0, 0.6 Hz, 1H, *H3a*), 4.79 (d, *J* = 11.5 Hz, 1H, *H9*), 4.75 (dd, *J* = 5.2, 0.6 Hz, 1H, *H2*), 4.64 (d, *J* = 11.5 Hz, 1H, *H9’*), 3.99 (d, *J* = 2.0 Hz, 1H, *H3*), 3.18 (d, *J* = 18.7 Hz, 1H, *H6*), 2.734 (dd, *J* = 13.8, 5.4 Hz, 1H, *H10_exo_*), 2.733 (d, *J* = 18.7 Hz, 1H, *H6’*), and 1.99 (d, *J* = 13.9 Hz, 1H, *H10_endo_*). ^13^C{^1^H} NMR (126 MHz, Chloroform-*d*) δ 175.2, 163.9, 87.6, 85.3, 85.2, 65.6, 48.5, 41.5, 32.7, and 24.6. IR (neat): 3003, 1777, 1749, 1221, 1193, and 1121. HRMS (ESI) *m/z*: [M + H]^+^ Calcd for C_10_H_10_IO_5_^+^ for 336.9573; Found 336.9558. mp: 184–186 °C.

#### (±)-(3a*R*,5*R*,7a*R*)-5-Hydroxy-4,5-dihydro-3a,7a-(methanooxymethano)benzofuran-2,10 (3*H*)-dione (7b).

The lactone acid **1** (210 mg, 1.0 mmol) and Sc(OTf)_3_ (49 mg, 0.1 mmol) were combined in a screw-capped culture tube. Acetonitrile (5.0 mL) was added. This mixture was placed in a preheated (70 °C) oil bath and kept there for 6 h. The brownish homogenous mixture was evaporated under vacuum and the crude material was purified by flash column chromatography (hexanes:EtOAc 1:1) to provide a sample of **7b** as a white crystalline solid, which contained an impurity that appeared to be itaconic acid (ca. 7 mol%). This material was recrystallized from ethanol to give (102 mg, 49%) of **7b**. ^1^H NMR (500 MHz, Acetone-*d*_6_): δ = 6.15 (ddd, *J* = 10.3, 2.2, 1.1 Hz, 1H, *H6*), 5.91 (dd, *J* = 10.3, 2.1 Hz, 1H, *H7*), 4.65 (dd, *J* = 11.1, 0.6 Hz, 1H, *H8*), 4.64 (dddd, *J* = 9.6, 5.0, 2.1, 2.1 Hz, 1H, *H5*), 4.49 (d, *J* = 11.2 Hz, 1H, *H8’*), 3.07 (d, *J* = 18.4 Hz, 1H, *H3*), 2.82 (d, *J* = 18.4 Hz, 1H, *H3’*), 2.36 (ddd, *J* = 13.6, 5.1, 1.2 Hz, 1H, *H4_eq_*), and 2.21 (dd, *J* = 13.5, 9.4 Hz, 1H, *H4_ax_*). ^13^C{^1^H} NMR (126 MHz, Acetone-*d*_6_): δ 178.2, 172.7, 138.2, 123.1, 86.6, 74.7, 62.8, 49.8, 35.5 and 34.6. IR (neat): 3439, 1776, 1251, 1061, and 936 cm^−1^. HRMS (ESI) *m/z*: [M + Na]^+^ Calcd for C_10_H_9_NaO_5_^+^ for 233.0426; Found 233.0411. mp: 144–145 °C.

#### (±)-(3a*R*,5*S*,7a*R*)-5-Hydroxytetrahydro-3a,7a-(methanooxymethano)benzofuran-2,10(3*H*)-dione (14).

A mixture of Sc(OTf)_3_ (5 mg, 0.01mmol) and the lactone acid **9** (21 mg, 0.1 mmol) were taken into a screw-capped culture tube. Acetonitrile-d_3_ (0.6 mL) was added. The resulting colorless homogenous solution was heated at 80 °C for 5 h. The solvent was evaporated under vacuum from the resulting colorless solution and the crude material was eluted through a silica gel plug with the aid of EtOAc. The eluent was concentrated and gave **14** (17 mg, 80%) as a white crystalline solid. The proton NMR spectrum of this reaction mixture showed a very clean conversion to the set of resonances for the product **14**. ^1^H NMR (500 MHz, Acetone-*d_6_*): δ 4.64 (d, *J* = 11.3 Hz, 1H, *H8*), 4.45 (dd, *J* = 11.3, 0.6 Hz, 1H, *H8’*), 3.97 (dddd, *J* = 10.5, 10.5, 4.0, 4.0 Hz, 1H, *H5*), 3.09 (d, *J* = 18.0 Hz, 1H, *H3*), 2.79 (dd, *J* = 18.0, 0.7 Hz, 1H, *H3*’), 2.9 (br s, 1H, O*H*), 2.29 (ddd, *J* = 14.2, 4.4, 3.6 Hz, 1H, *H7_eq_*), 2.23 (ddd, *J* = 13.8, 4.0, 2.2, Hz, 1H, *H4_eq_*), 1.98 (ddddd, *J* = 13.5, 4.6 3.8, 3.8, 2.2 Hz, 1H, *H6_eq_*), 1.90 (ddd, *J* = 14.5, 13.4, 4.7 Hz, 1H, *H7_ax_*), 1.62 (ddd, *J* = 13.8, 10.6, 0.4 Hz, 1H, *H4_ax_*), and 1.50 (dddd, *J* = 13.5, 13.5, 10.2, 4.2 Hz, 1H, *H6_ax_*).^1^H NMR (500 MHz, MeCN-*d_3_*): δ 4.49 (d, *J* = 11.2 Hz, 1H, *H8*), 4.42 (dd, *J* = 11.3, 0.5 Hz, *H8’*), 3.80 (dddd, *J* = 10.3, 10.3, 3.8, 3.8 Hz, 1H, *H5*), 2.92 (d, *J* = 18.1 Hz, 1H, *H3*), 2.80 (dd, *J* = 18.1, 0.6 Hz, 1H, *H3*’), 2.22 (ddd, *J* = 14.5, 4.3, 3.8 Hz, 1H, *H7_eq_*), 2.15 (ddd, *J* = 13.8, 3.9, 2.2, Hz, 1H, *H4_eq_*), 1.91 (ddddd, *J* = 13.5, 4.8 3.7, 3.7, 2.2 Hz, 1H, *H6_eq_*), 1.79 (ddd, *J* = 14.4, 13.3, 4.7 Hz, 1H, *H7_ax_*), 1.55 (ddd, *J* = 13.9, 10.5, 0.4 Hz, 1H, *H4_ax_*), and 1.36 (dddd, *J* = 13.5, 13.5, 10.1, 4.1 Hz, 1H, *H6_ax_*). ^13^C{1H} NMR (126 MHz, Acetone-d6): δ 178.2, 172.0, 87.3, 70.9, 63.7, 49.2, 35.4, 35.2, 30.4, and 27.6. IR (neat): 3052, 3001, 2952, 2899, 2835, 2228, 1702, 1156, 1113, 1020, 999, 883, and 764 cm^−1^. HRMS (ESI) *m/z*: [M + Na]^+^ Calcd for C_10_H_12_NaO_5_^+^ for 235.0582; Found 235.0568. mp: 116–117 °C.

#### (±)-3-Hydroxydihydro-2*H*,7*H*,9*H*-2,6a-methanodifuro[3,2-*b*:3’,4’-*c*]pyran-5,7(6*H*)-dione (15).

##### Method A:

The epoxy acid **11** (1.59 g, 6.2 mmol) was added to a 250 mL round bottom flask. THF (70 mL) was added, followed by BF_3_.OEt_2_ (0.44 mL, 3.5 mmol). This mixture was placed in a preheated (70 °C) oil bath and kept there for 12 h. The resulting colorless solution was evaporated under vacuum to obtain a white sticky solid. This solid was triturated with Et_2_O, aided by sonication. The white solid was filtered under vacuum and washed several times with additional fresh Et_2_O. The solid was finally recrystallized from ethanol to provide the sample of **15** (1.35 g, 85%) as a white crystalline solid.

##### Method B:

The lactone epoxide **11** (22 mg, 0.1 mmol) and Sc(OTf)_3_ (4.9 mg, 0.01 mmol) were combined in a screw-capped culture tube. Acetonitrile-*d_3_* (0.6 mL) was added to the NMR tube. The colorless homogenous mixture was monitored at room temperature. After full consumption of the starting epoxide (36 h), the crude material was passed through a short pad of silica and eluted with 5 mL of EtOAc. The solution was evaporated to provide the white solid material, which was then washed with several mL of Et_2_O to give the sample of **15** (19 mg, 84%) as a white crystalline solid. ^1^H NMR (500 MHz, Acetone-*d*_6_) δ 4.92 (dd, *J* = 11.4, 0.5 Hz, 1H, *H9β*), 4.66 (d, *J* = 11.4 Hz, 1H, *H9α*), 4.65 (dd, *J* = 1.2, 1.2 Hz, 1H, *H3a*), 4.42 (dd, *J* = 5.8, 1.3 Hz, 1H, *H2*), 4.13 (dd, *J* = 1.2 Hz, 1H, *H3*), 3.05 (d, *J* = 18.7 Hz, 1H, *H6α*), 2.94 (dd, *J* = 18.6, 0.6 Hz, 1H, *H6β*), 2.47 (dd, *J* = 13.6, 5.9 Hz, 1H, *H1α*), and 2.06 (d, *J* = 13.7 Hz, 1H, *H1β*). ^13^C{1H} NMR (126 MHz, Acetone-*d*_6_) δ 177.3 (C7), 165.4 (C5), 85.9 (3a), 84.6 (*9a*), 84.0 (C2), 81.2 (C3), 66.9 (C9), 49.6 (6a), 38.0 (C1), and 32.5 (C6). IR (neat): 3453, 1776, 1745, 1378, 1223, 1065, and 1008 cm^−1^. HRMS (ESI) *m/z*: [M + Na]^+^ Calcd for C_10_H_10_NaO_6_^+^ for 249.0375; Found 249.0357. mp: 196–198 °C.

#### (±)-(3a*R*,5*R*,7a*R*)-2,10-Dioxo-2,3,4,5-tetrahydro-3a,7a-(methanooxymethano)benzofuran-5-yl acrylate (16a).

A mixture of the lactone acid **1** (210 mg, 1.0 mmol) and Sc(OTf)_3_ (49 mg, 0.1 mmol) were combined in a screw-capped culture tube. Acetonitrile (5.0 mL) and acrylic anhydride (230 mL, 1.95 mmol) were added. The homogenous mixture was stirred at room temperature for 20 h. The solvent was evaporated under vacuum and the crude material was purified by MPLC (hexanes:EtOAc 4:6) to provide the sample of **16a** (176 mg, 67%) as a white crystalline solid. ^1^H NMR (500 MHz, Chloroform-*d*): = 6.42 (dd, *J=* 17.3, 1.5 Hz, 1H, *H_Z_*H_E_C=C), 6.20 (ddd, *J* = 10.3, 3.2, 0.8 Hz, 1H, *H6*), 6.10 (dd, *J* = 10.3, 1.6 Hz, 1H, *H7*), 6.09 (dd, *J=* 17.3, 10.5 Hz, 1H, =CHC=O), 5.92 (dd, *J=* 10.5, 1.3 Hz, 1H, H_Z_*H_E_*C=C), 5.56 (dddd, *J* = 8.1, 4.7, 3.2, 1.6 Hz, 1H, *H5*), 4.69 (d, *J* = 11.0 Hz, 1H, *H8*), 4.38 (d, *J* = 11.0 Hz, 1H, *H8’*), 3.09 (d, *J* = 18.6 Hz, 1H, *H3*), 2.84 (d, *J* = 18.6 Hz, 1H, *H3’*), 2.40 (ddd, *J* = 13.9, 4.7, 0.8 Hz, 1H, *H4_eq_*) and 2.21 (dd, *J* = 13.9, 8.1 Hz, 1H, *H4_ax_*). ^13^C{^1^H} NMR (126 MHz, Chloroform-d): δ 176.5, 171.4, 165.1, 132.6, 131.7, 127.5, 126.4, 84.7, 74.9, 64.8, 47.8, 36.2 and 31.5. IR (neat): 2989, 1781, 1721, 1634, 1189, 1051, and 941 cm^−1^. HRMS (ESI) *m/z:* [M + H]^+^ Calcd for C_13_H_13_O_6_^+^ for 265.0712; Found 265.0696. mp: 122–123 °C.

#### (±)-(3a*R*,5*R*,7a*R*)-2,10-Dioxo-2,3,4,5-tetrahydro-3a,7a-(methanooxymethano)benzofuran-5-yl methacrylate (16b).

A mixture of the lactone acid **1** (2.2 g, 10.5 mmol) and Sc(OTf)_3_ (515 mg, 1.05 mmol) were combined in a screw-capped culture tube. Acetonitrile (30 mL) and methacrylic anhydride (2.2 mL, 15.7 mmol) were added. The homogenous mixture was stirred at room temperature for 16 h. The solvent was evaporated under vacuum and the crude material was purified by flash column chromatography (hexanes:EtOAc 4:6) to provide the sample of **16b** (1.9 gm, 65%) as a white crystalline solid. ^1^H NMR (500 MHz, Chloroform-*d*): δ 6.24 (ddd, *J* = 10.2, 3.4, 0.6 Hz, 1H, *H6*), 6.12 (dd, *J* = 10.3, 1.4 Hz, 1H, *H7*), 6.09 (dq, *J* = 1.1, 1.1 Hz, 1H, *H_Z_*H_E_C=C), 5.65 (dq, *J* = 1.5, 1.5 Hz, 1H, H_Z_*H_E_*C=C), 5.53 (dddd, *J* = 7.5, 4.8, 3.4, 1.4 Hz, 1H, *H5*), 4.69 (d, *J* = 10.9 Hz, 1H, *H8*), 4.38 (d, *J* = 11.0 Hz, 1H, *H8’*), 3.11 (d, *J* = 18.6 Hz, 1H, *H3*), 2.85 (d, *J* = 18.6 Hz, 1H, *H3’*), 2.35 (ddd, *J* = 14.0, 4.7, 0.6 Hz, 1H, *H4*), 2.29 (dd, *J* = 14.0, 7.5 Hz, 1H, *H4’*), and 1.94 (dd, *J* = 1.7, 1.0 Hz, 3H, -C*H_3_*). ^13^C{^1^H} NMR (126 MHz, Chloroform-d): δ 176.6, 171.5, 166.4, 135.5, 131.6, 127.2, 126.7, 84.7, 75.3, 64.8, 47.6, 36.7, 31.8, and 18.3. IR (neat): 1781, 1713, 1635, and 1168 cm^−1^. HRMS (ESI) *m/z*: [M + H]^+^ Calcd for C_14_H_15_O_6_^+^ for 279.0869; Found 279.0851. mp: 105–106 °C.

#### (±)-(3aR,5R,7aR)-2,10-Dioxo-2,3,4,5-tetrahydro-3a,7a-(methanooxymethano)benzofuran-5-yl Acetate (16c).

A mixture of Sc(OTf)_3_ (49 mg, 0.1mmol) and the lactone acid **1** (210 mg, 1 mmol) were taken into a screw-capped culture tube. Acetonitrile (5 mL) and acetic anhydride (190 μL, 2.0 mmol) were added. The dark green-colored homogenous mixture was stirred at room temperature for 16 h. The solvent was evaporated under vacuum and the crude material was purified by flash column chromatography (hexanes:EtOAc, 1:1) to provide the sample of **16c** (130 mg, 51%) as a sticky oil. ^1^H NMR (500 MHz, CDCl_3_): δ 6.13 (ddd, *J* = 10.4, 3.0, 0.8 Hz, 1H, *H6*), 6.07 (ddd, *J* = 10.3, 1.6 Hz, 1H, *H7*), 5.49 (dddd, *J* = 8.1, 4.7, 3.0, 1.6 Hz, 1H, *H5*), 4.67 (dd, *J* = 11.0, 0.5 Hz, 1H, *H8*), 4.37 (d, *J* = 11.0 Hz, 1H, *H8’*), 3.06 (d, *J* = 18.6 Hz, 1H, *H3*), 2.82 (d, *J* = 18.5 Hz, 1H, *H3’*), 2.38 (ddd, *J* = 14.0, 4.8, 0.8 Hz, 1H, *H4*), 2.12 (dd, *J* = 14.0, 8.3 Hz, 1H, *H4’*), and 2.07 (s, 3H). ^13^C{1H} NMR (126 MHz, CDCl_3_) δ 176.5 (*C*10), 171.4 (*C*2), 170.1 (CH_3_*C*O_2_), 131.8 (*C*6), 126.1 (*C*7), 84.7 (*C*7a), 74.7 (*C*8), 64.6 (*C*5), 47.9 (*C*3a), 36.0 (*C*3), 31.2 (*C*4), and 20.9 (*C*H_3_CO_2_). IR (neat): 3023, 2937, 1775, 1732, 1373, 1224, 1009, and 938 cm^−1^. HRMS (ESI) *m/z*: [M + Na]^+^ Calcd for C_12_H_12_NaO_6_^+^ for 275.0532; Found 275.0515.

#### (3a*R*,5*S*,7a*R*)-2,10-Dioxohexahydro-3a,7a-(methanooxymethano)benzofuran-5-yl acrylate (17a).

A mixture of Sc(OTf)_3_ (38 mg, 0.094 mmol) and the lactone acid **9** (200 mg, 0.94 mmol) was taken into a screw-capped culture tube. Acetonitrile (2 mL) was added followed by acrylic anhydride (178 mL 1.9 mmol). The colorless homogeneous mixture was stirred at room temperature for 16 h. The solvent was evaporated under vacuum and the crude material was purified by flash column chromatography (hexanes:EtOAc, 2:3) to provide the sample of **17a** (186 mg, 0.70 mmol 75%) as a white crystalline solid. ^1^H NMR (500 MHz, Acetone-*d_6_*): δ = 6.35 (dd, *J=* 17.2, 1.5 Hz, 1H, *H_Z_*H_E_C=C), 6.11 (dd, *J=* 17.2, 10.5 Hz, 1H, CHCOO), 5.90 (dd, *J=* 10.5, 1.5 Hz, 1H, *H_Z_*H_E_C=C), 5.11 (dddd, *J=* 9.9, 9.9, 3.9, 3.9, 1H, H*5*), 4.70 (d, *J* = 11.3 Hz, 1H, *H8*),, 4.50 (d, *J* = 11.3, 0.5 Hz, 1H, *H8’*), 3.20 (d, *J* =18.0 Hz, 1H, *H3*) , 2.89 (dd, *J* =18.0, 0.6 Hz, 1H, *H3’*), 2.39–2.34 (nfom, 1H, *H6^exo^*), 2.13–2.06 (two nfom, 2H, *H6^endo^* and *H7*), 2.34 (ddd, *J* = 13.8, 4.0, 1.8 Hz, 1H, *H4^exo^*), 1.95 (ddd, *J* = 13.9, 9.9, 0.4 Hz, 1H, *H4^endo^*), and 1.84–1.70 (nfom, 1H, *H7’*). ^13^C NMR (126 MHz, Acetone-*d_6_*): δ = 178.3, 172.6, 165.5, 131.6, 129.3, 87.5, 72.2, 68.2, 49.3, 36.4, 32.5, 27.8, and 27.2. IR (neat): 2949, 2874, 1779, 1719, 1409, 1190, and 764 cm^−1^. HRMS (ESI) *m/z:* [M + H]^+^ Calcd for C_13_H_15_O_6_^+^ for 267.0869; Found 267.0851. mp: 138–140 °C.

#### (±)-(3a*R*,5*S*,7a*R*)-2,10-Dioxohexahydro-3a,7a-(methanooxymethano)benzofuran-5-yl acetate (17c).

A mixture of Sc(OTf)_3_ (215 mg, 0.47 mmol) and the lactone acid **9** (1 g, 4.7 mmol) were taken into a screw-capped culture tube. Acetonitrile (8 mL) followed by acetic anhydride (1.1 mL, 9.4 mmol) was added. The colorless homogenous mixture was stirred at room temperature for 16 h. The solvent was evaporated under vacuum and the crude material was purified by flash column chromatography (hexanes:EtOAc, 4:6) to provide the sample of **17c** (980 mg, 82%) as a white crystalline solid. ^1^H NMR (500 MHz, CDCl_3_): 4.91 (dddd, *J* = 9.3, 8.9, 3.9, 3.9 Hz, 1H, *H5*), 4.48 (dd, *J* = 11.1, 0.7 Hz, 1H, *H8*), 4.38 (d, *J* = 11.0 Hz, 1H, *H8’*), 3.03 (dd, *J* = 18.3, 0.7 Hz, 1H, *H3*), 2.78 (d, *J* = 18.3 Hz, 1H, *H3’*), 2.28 (ddd, *J* = 14.9, 4.7, 4.4 Hz, 1H, *H7*), 2.21 (ddd, *J* = 14.3, 3.9, 1.9 Hz, 1H, *H4*), 2.08 (ddddd, *J* = 14.0, 4.9, 4.9, 3.9, 1.9 Hz, 1H, *H6*), 2.03 (s, 3H, COC*H_3_*), 1.90 (dd, *J* = 14.1, 9.2 Hz, 1H, *H4’*), 1.89 (dd, *J* = 14.9, 12.1, 5.0 Hz, 1H, *H7’*), and 1.54 (dddd, *J* = 13.9, 11.9, 8.8, 4.5 Hz, 1H, *H6’*). ^13^C{1H} NMR (126 MHz, CDCl_3_) δ 176.8 (C10), 171.6 (C2), 170.1 (CH_3_*C*O), 86.2 (C7a), 71.9 (C8), 66.5 (C5), 48.0 (C3a), 36.4 (C3), 32.5 (C4), 27.7 (C7), 26.6 (C6), and 21.1 (*C*H_3_CO). IR (neat): 1780, 1750, 1727, 1185, and 1063 cm^−1^. HRMS (ESI) *m/z:* [M + Na]^+^ Calcd for C_12_H_14_NaO_6_^+^ for 277.0688; Found 277.0672. mp: 125–127 °C.

In a separate experiment, this transformation was monitored over time by in situ ^1^H NMR spectroscopy in CD_3_CN at ambient temperature. Sc(OTf)_3_ (10 mol% ), Ac_2_O (18 uL), and acid **1** were used. The reaction was very selective for formation of the acetate **17c**. A copy of the spectrum of the reaction mixture sample is included at the end of this SI. The following spectral data were taken from a NMR sample of purified **17c** in CD_3_CN. ^1^H NMR (500 MHz, CD_3_CN): δ 4.91 (dddd, *J* = 9.8, 9.8, 4.0, 4.0 Hz, 1H, *H5*), 4.51 (d, *J* = 11.2 Hz, 1H, *H8*), 4.38 (dd, *J* = 11.4, 0.8 Hz, 1H, *H8’*), 2.96 (d, *J* = 18.2 Hz, 1H, *H3*), 2.78 (dd, *J* = 18.3, 0.8 Hz, 1H, *H3’*), 2.26 (ddd, *J* = 14.6, 4.2, 4.2 Hz, 1H, *H7*), 2.20 (ddd, *J* = 14.0, 4.0, 2.0 Hz, 1H, *H4*), 2.00 (ddddd, *J* = 13.5, 4.9, 4.2, 4.2 1.9 Hz, 1H, *H6*), 1.97 (s, 3H, COC*H_3_*), 1.91 (ddd, *J* = 14.7, 12.6, 4.9 Hz, 1H, *H7’*), 1.83 (dd, *J* = 14.0, 10.0 Hz, 1H, *H4’*), and 1.54 (dddd, *J* = 13.7, 12.7, 9.5, 4.3 Hz, 1H, *H6’*).

#### (±)-(3a*R*,5*S*,7a*R*)-2,10-Dioxohexahydro-3a,7a-(methanooxymethano)benzofuran-5-yl methacrylate (17b).

A mixture of the lactone acid **9** (5.0 g, 23.6 mmol) and Sc(OTf)_3_ (580 mg, 1.18 mmol) were combined in a screw-capped culture tube. Acetonitrile (50 mL) and methacrylic anhydride (5.3 mL, 35.3 mmol) were added. The homogenous mixture was stirred at room temperature for 16 h. The solvent was evaporated under vacuum and the crude material was purified by flash column chromatography (hexanes:EtOAc 4:6) to provide the sample of **17b** (5.1 gm, 76%) as a white crystalline solid. ^1^H NMR (500 MHz, Chloroform-d): δ 6.08 (dq, *J* = 1.5, 1.0 Hz, 1H, *H_Z_*H_E_C=C), 5.61 (dq, *J* = 1.5, 1.5 Hz, 1H, H_Z_*H_E_*C=C), 5.01 (dddd, *J* = 8.7, 8.7, 3.9, 3.9 Hz, 1H, *H5*), 4.49 (dd, *J* = 11.0, 0.4 Hz, 1H, *H8*), 4.40 (d, *J* = 11.0 Hz, 1H, *H8’*), 3.06 (dd, *J* = 18.3, 0.6 Hz, 1H, *H3*), 2.81 (d, *J* = 18.3 Hz, 1H, *H3’*), 2.29 (ddd, *J* = 14.8, 5.5, 4.5 Hz, 1H, *H7_eq_*), 2.24 (ddd, *J* = 14.4, 3.8, 1.6 Hz, 1H, *H4_eq_*), 2.13 (ddddd, *J* = 14.1, 5.2, 5.1, 4.1, 1.6 Hz, 1H, *H6_eq_*), 2.03 (dd, *J* = 14.3, 8.8 Hz, 1H, *H4_ax_*), 1.96 (ddd, *J* = 14.9, 11.5, 4.8 Hz, 1H, *H7_ax_*), 1.92 (dd, *J* = 1.0, 1.5 Hz, 3H, -C*H_3_*), and 1.63 (dddd, *J* = 14.2, 11.5, 8.5, 4.5 Hz, 1H, *H6’*). ^13^C{^1^H} NMR (126 MHz, Chloroform-d): δ 176.7 (C10), 171.7 (C2), 166.4 (=C*C*=O), 135.8 (=*C*C=O), 126.7 (=*C*H_2_), 86.2 (C7a), 72.1 (C8), 66.8 (C5), 47.9 (C3a), 36.7 (C3), 32.7 (C4), 27.6 (C7), 26.4 (C6), and 18.3 (CH_3_). IR (neat): 2957, 1781, 1714, 1635, and 1168 cm^−1^. HRMS (ESI) *m/z*: [M + H]^+^ Calcd for C_14_H_17_O_6_^+^ for 281.1025; Found 281.1006. mp: 155–157 °C.

#### (±)-(3aR,5S,7aR)-2,10-Dioxohexahydro-3a,7a-(methanooxymethano)benzofuran-5-yl Benzoate (17d).

A mixture of Sc(OTf)_3_ (25 mg, 0.05 mmol) and the lactone acid **9** (106 mg, 0.5 mmol) were taken into a screw-capped culture tube. Acetonitrile (3 mL) and benzoyl chloride (116 μL, 1.0 mmol) were added. The colorless homogenous mixture was stirred at room temperature for 16 h. The solvent was evaporated under vacuum and the crude material was purified by flash column chromatography (hexanes:EtOAc, 1:1) to provide the **17d** (128 mg, 81%) as a white crystalline solid. ^1^H NMR (500 MHz, CDCl_3_): δ 7.98 (nfom, 2H, *Ph_o+o’_*), 7.63 (tt, *J* = 7.5, 1.4 Hz, 1H, *Ph_p_*), 7.42 (nfom, 2H, *Ph_m+m’_*), 5.19 (dddd, *J* = 9.0, 9.0, 3.9, 3.9 Hz, 1H, *H5*), 4.52 (dd, *J* = 11.0, 0.6 Hz, 1H, *H8*), 4.44 (d, *J* = 11.0 Hz, 1H, *H8’*), 3.09 (dd, *J* = 18.2, 0.6 Hz, 1H, *H3*), 2.78 (d, *J* = 18.3 Hz, 1H, *H3’*), 2.36 (ddd, *J* = 14.3, 4.0, 1.8 Hz, 1H, *H4_eq_*), 2.33 (ddd, *J* = 14.9, 5.1, 4.4 Hz, 1H, *H7_eq_*), 2.23 (ddddd, *J* = 14.0, 4.9, 4.9, 3.8, 1.8 Hz, 1H, *H6_eq_*), 2.10 (dd, *J* = 14.3, 9.1 Hz, 1H, *H4_ax_*), 2.0 (ddd, *J* = 14.9, 11.9, 4.9 Hz, 1H, *H7_ax_*), and 1.72 (dddd, *J* = 14.1, 11.8, 8.7, 4.4 Hz, 1H, *H6_ax_*). ^13^C{1H} NMR (126 MHz, CDCl_3_) δ 176.8, 171.7, 165.7, 133.6, 129.8, 129.5, 128.7, 86.3, 72.0, 67.2, 48.0, 36.6, 32.8, 27.7 and 26.7. IR (neat): 1780, 1714, 1274, 1113, and 714 cm^−1^. HRMS (ESI) *m/z*: [M + Na]^+^ Calcd for C_17_H_16_NaO_6_^+^ for 339.0845; Found 339.0823. mp: 143–144 °C.

#### (±)-(3aR,5S,7aR)-2,10-Dioxohexahydro-3a,7a-(methanooxymethano)benzofuran-5-yl Pivalate (17e).

A mixture of Sc(OTf)_3_ (25 mg, 0.05 mmol) and the lactone acid **9** (106 mg, 0.5 mmol) were taken into a screw-capped culture tube. Acetonitrile (3 mL) and pivaloyl chloride (123 μL, 1.0 mmol) were added. The colorless homogenous mixture was stirred at room temperature for 20 h. The solvent was evaporated under vacuum and the crude material was purified by flash column chromatography (hexanes:EtOAc, 1:1) to provide the pivalate **17e** (105 mg, 71 %) as a white crystalline solid. ^1^H NMR (500 MHz, CDCl_3_): δ 5.0 (dddd, *J* = 9.3, 9.3, 4.0, 4.0 Hz, 1H, *H5*), 4.68 (dd, *J* = 11.2, 0.5 Hz, 1H, *H8*), 4.50 (d, *J* = 11.2 Hz, 1H, *H8’*), 3.13 (dd, *J* = 18.1, 0.5 Hz, 1H, *H3*), 2.90 (d, *J* = 18.1 Hz, 1H, *H3’*), 2.32 (ddd, *J* = 14.2, 4.5, 1.9 Hz, 1H, *H4_eq_*), 2.25 (ddd, *J* = 14.0, 4.0, 1.8 Hz, 1H, *H7_eq_*), 2.08 (dd, *J* = 14.4, 12.1, 4.7 Hz, 1H, *H7_ax_*), 2.03 (dddd, *J* = 13.7, 4.9, 3.9, 1.9 Hz, 1H, *H6_eq_*), 1.95 (dd, *J* = 14.1, 9.5 Hz, 1H, *H4_ax_*), and 1.70 (dddd, *J* = 13.7, 11.9, 9.1, 5.3 Hz, 1H, *H6_ax_*). ^13^C{1H} NMR (126 MHz, CDCl_3_) δ 177.3, 176.7, 171.8, 86.5, 71.5, 66.7, 48.1, 38.3, 35.9, 31.7, 26.8, 26.4, and 26.1. IR (neat): 2971, 1780, 1721, 1281, and 1154 cm^−1^. HRMS (ESI) *m/z:* [M + Na]^+^ Calcd for C_15_H_20_NaO_6_^+^ for 319.1158; Found 319.1133. mp: 86–88 °C.

#### (±)-5,7-Dioxotetrahydro-2*H*,7*H*,9*H*-2,6a-methanodifuro[3,2-b:3’,4’-c]pyran-3-yl acrylate (18a).

A mixture of the lactone epoxide **11** (252 mg, 2 mmol) and Sc(OTf)_3_ (98 mg, 0.2 mmol) was placed into a screw-capped culture tube. Acetonitrile (8 mL) and acrylic anhydride (400 µL, 3.4 mmol) were added. The homogenous mixture was stirred at room temperature for 12 h. The solvent was evaporated under vacuum and the crude material was purified by flash column chromatography (hexanes:EtOAc 4:6) to provide **18a** (354 mg, 63%) as a white crystalline solid. ^1^H NMR (500 MHz, Acetone-*d_6_*) δ = 6.43 (dd, *J=* 17.3, 1.5 Hz, 1H, H_Z_*H_E_*C=C), 6.21 (dd, *J=* 17.3, 10.4 Hz, 1H, CHCOO), 5.98 (dd, *J=* 10.5, 1.5 Hz, 1H, *H_Z_*H_E_C=C), 5.12 (d, *J* = 1.4 Hz, 1H, *H3*), 5.00 (d, *J* = 11.6 Hz, 1H, *H9*), 4.98 (dd, *J* = 1.3, 1.3 Hz, 1H, *H3a*), 4.72 (d, *J* = 11.5 Hz, 1H, *H9’*), 4.67 (dd, *J* = 5.8, 1.2 Hz, 1H, *H2*), 3.17 (d, *J* = 18.8 Hz, 1H, *H6*), 3.01 (d, *J* = 18.8 Hz, 1H, *H6’*), 2.59 (dd, *J* = 13.8, 6.0 Hz, 1H, *H1_exo_*), and 2.34 (d, *J* = 13.8 Hz, 1H, *H1_endo_*). ^13^C{^1^H} NMR (126 MHz, Acetone-*d*_6_) δ 177.0, 165.6, 165.0, 132.6, 128.6, 85.0, 82.7, 82.6, 81.7, 66.7, 49.6, 37.8, and 32.3. IR (neat): 1779, 1749, 1725, 1633, 1184, and 1063 cm^−1^. HRMS (ESI) *m/z*: [M + H]^+^ Calcd for C_13_H_13_O_7_^+^ for 281.0661; Found 281.0645. mp: 197–199 °C.

#### (±)-(5,7-Dioxotetrahydro-2*H*,7*H*,9*H*-2,6a-methanodifuro[3,2-*b*:3’,4’-*c*]pyran-3-yl methacrylate (18b).

##### Smaller scale (with chromatography):

A mixture of lactone epoxide **11** (226 mg, 1 mmol) and Sc(OTf)_3_ (49 mg, 0.1 mmol) was placed into a screw-capped culture tube. Acetonitrile (6 mL) and methacrylic anhydride (220 µL, 1.5 mmol) were added. The homogenous mixture was stirred at room temperature for 12 h. The solvent was evaporated under vacuum and the crude material was purified by flash column chromatography (hexanes:EtOAc 4:6) to provide the sample of **18b** (220 mg, 75%) as a white crystalline solid.

##### Larger scale (with recrystallization):

A mixture of the lactone epoxide **11** (4.0 g, 17.7 mmol) and Sc(OTf)_3_ (712 mg, 1.8 mmol) was placed into a screw-capped culture tube. Acetonitrile (120 mL) and methacrylic anhydride (4.0 mL, 35.4 mmol) were added. The homogenous mixture was stirred at room temperature for 12 h. The solvent was evaporated under vacuum to obtain a white solid. This solid was washed several times with Et_2_O (these washing contained largely methacrylic acid and anhydride; ^1^H NMR). The solid was recrystallized from hexane and methanol to provide the sample of **18b** (4.3 g, 82%) as a white crystalline solid. ^1^H NMR (500 MHz, Acetone-*d_6_*) δ 6.14 (dq, *J* = 1.5, 1.1 Hz, 1H, *H_Z_*H_E_C=C), 5.72 (dq, *J* = 1.6, 1.6 Hz, 1H, H_Z_*H_E_*C=C), 5.09 (d, *J* = 1.4 Hz, 1H, *H3*), 5.00 (d, *J* = 11.5 Hz, 1H, *H9*), 4.98 (dd, *J* = 1.3, 1.3 Hz, 1H, *H3a*), 4.72 (d, *J* = 11.5 Hz, 1H, *H9’*), 4.67 (dd, *J* = 5.9, 1.3 Hz, 1H, *H2*), 3.18 (d, *J* = 18.8 Hz, 1H, *H6*), 3.01 (d, *J* = 18.8 Hz, 1H, *H6’*), 2.59 (dd, *J* = 13.8, 5.9 Hz, 1H, *H1_exo_*), 2.34 (d, *J* = 13.7 Hz, 1H, *H1_endo_*), and 1.93 (dd, *J =* 1.6, 1.0 Hz, 3H, -C*H_3_*). ^13^C{^1^H} NMR (126 MHz, Acetone-*d*_6_) δ 177.0, 166.8, 165.0, 136.7, 126.8, 85.0, 82.8, 82.6, 81.7, 66.7, 49.6, 37.9, 32.3, and 18.2. IR (neat): 3052, 3001, 2952, 2899, 2835, 1783, 1719, 1702, 1156, 1113, 1020, 999, 883, and 764 cm^−1^. HRMS (ESI) *m/z:* [M + H]^+^ Calcd for C_14_H_15_O_7_^+^ for 295.0818; Found 295.0800. mp: 169–170 °C.

#### (±)-5,7-Dioxotetrahydro-2*H*,7*H*,9*H*-2,6a-methanodifuro[3,2-*b*:3’,4’-*c*]pyran-3-yl acetate (18c).

A mixture of Sc(OTf)_3_ (36 mg, 0.07 mmol) and epoxy lactone acid **11** (163 mg, 0.73 mmol) were combined in a screw-capped culture tube. Acetonitrile (1.5 mL) and acetic anhydride (140 μL, 1.48 mmol) were added. The colorless homogenous mixture was stirred at room temperature for 16 h. The solvent was evaporated under vacuum and the crude material was purified by flash column chromatography (hexanes:EtOAc, 4:6) to provide the sample of **18c** (165 mg, 84%) as a white crystalline solid. ^1^H NMR (500 MHz, Acetone-*d*_6_) δ 5.01 (d, *J* = 1.4 Hz, 1H, *H3*), 4.98 (dd, *J* = 11.5, 0.5 Hz, 1H, *H9β*), 4.89 (dd, *J* = 1.3, 1.3 Hz, 1H, *H3a*), 4.70 (d, *J* = 11.6 Hz, 1H, *H9α*), 4.60 (dd, *J* = 5.9, 1.3 Hz, 1H, *H2*), 3.15 (d, *J* = 18.8 Hz, 1H, *H6α*), 3.00 (dd, *J* = 18.8, 0.5 Hz, 1H, *H6β*), 2.56 (dd, *J* = 13.7, 5.9 Hz, 1H, *H1α*), 2.30 (d, *J* = 13.7 Hz, 1H, *H1β*), and 2.08 (s, 3H). ^13^C{1H} NMR (126 MHz, Acetone-*d*_6_) δ 177.0 (C7), 170.4 (Me*C*O), 165.0 (C5), 84.9 (9a), 82.7(C3a), 82.4 (C3), 81.8 (C2), 66.7 (C9), 49.6 (6a), 37.9 (C1), 32.3 (C6), and 20.7 (CH_3_). IR (neat): 2990, 1780, 1750, 1374, and 1237 cm^−1^. HRMS (ESI) *m/z:* [M + Na]^+^ Calcd for C_12_H_12_NaO_7_^+^ for 291.0481; Found 291.0462. mp: 197–198 °C.

## Supplementary Material

Supplementary Information
