# Supplementary material for: Novel Conversions of a Multifunctional, Bio-sourced Lactone Carboxylic Acid
Source: ARKIVOC. Author manuscript; Available in PMC 2024 Feb 15. (PMC10869105; doi:10.24820/ark.5550190.p012.081)
Supplement: Supplementary Information [file NIHMS1964371-supplement-Supplementary_Information.pdf]

Supplementary Material

Novel Conversions of a Multifunctional, Bio-sourced Lactone Carboxylic Acid

Trinadh Kaicharla, Sangjun Lee, Ruiqin Wang, Ashok D. Pehere, Shu Xu, and Thomas R. Hoye*

Department of Chemistry, 207 Pleasant St. SE, University of Minnesota, Minneapolis, MN 55455

email: hoye@umn.edu

Table of Contents

General Experimental Protocols S2

Experimental Procedures for Synthesis and Characterization of All New Compounds S3–S23

Supplementary Material References S24

Copies of ^{1}H and ^{13}C NMR Spectra S24–S86

General Experimental Protocols:

Nuclear magnetic resonance (NMR) spectra (^{1}H and ^{13}C) were recorded on a Bruker HD-500 spectrometer. ^{1}H chemical shifts in $CDCl_3$ samples are referenced to TMS (δ 0.00), in acetone-d_6 samples to residual solvent protons at 2.05, DMSO-d_6 to residual solvent protons at 2.50, and in CD3CN to residual solvent protons at 1.94. Data are reported using the following format: chemical shift (ppm) [multiplicity, coupling constant(s) (in Hz), integral (to the nearest whole integer), and assignment of the proton]. Coupling constant values have been deduced using protocols previously described.[1,2] Non-first order multiplets in a ^{1}H NMR spectrum are designated by 'nfom'. Non-first order doublets in a ^{1}H NMR spectrum (e.g., present in a 1,4-disubstitutedbenzene ring) are designated by 'nfod' and the apparent doublet coupling constant (actually, e.g., $J_{2,3} + J_{2,3'}$) is indicated as J_{app}. ^{13}C{^{1}H} NMR chemical shifts are referenced to the carbon atom in $CDCl_3$ to 77.16 ppm, in acetone-d_6 to 29.8 ppm, and in DMSO-d_6 to 39.5 ppm.

Infrared (IR) spectral data were collected on a Bruker spectrometer (model Alpha II). Samples were deposited as films on a diamond window (solids by evaporation from DCM; liquids by direct application) in the mode of attenuated total reflectance (ATR). Peaks are reported in cm^{-1}.

High-resolution mass spectrometry (HRMS) measurements were made using ESI ionization with a Thermo instrument (model Orbitrap Velos, which has a mass accuracy of ≤3). An external calibrant (Pierce™ LTQ) was used. Samples were injected directly into the ion source.

Medium pressure liquid chromatography (MPLC) was used to purify products. Silica gel (normal-phase, 20-40 µm, 60 Å pore size, Teledyne RediSep Rf Gold®) columns were hand-packed into Michel-Miller® glass columns. The equipment used consisted of a Waters HPLC pump (model 510) and a Waters (R401) differential refractive index detector. Preparative flash chromatography was done on silica gel (230-400 mesh) columns. Thin layer chromatography (TLC) was performed on silica-gel-coated, plastic-backed plates (Machery-Nagel) that were visualized by staining with $KMnO_4$ solution.

Heating of reactions performed above ambient temperature was done in silicone oil baths that had been preequilibrated to the desired temperature prior to immersion of the reaction vessel.

(±)-3a,7a-(Methanooxymethano)benzofuran-2,10(3*H*)-dione (5) and 3-Isochromanone (6)

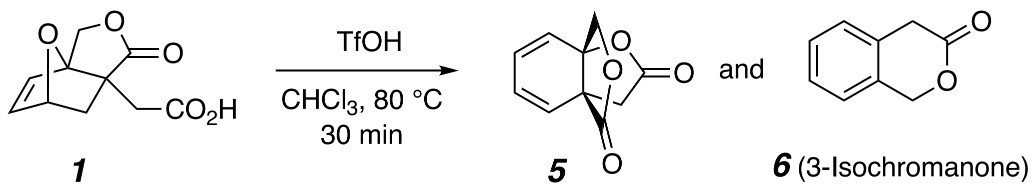

The lactone acid **1** (200 mg, 0.95 mmol) was suspended in chloroform (10 mL) in a 20 mL Schlenk flask. Trifluoromethanesulfonic acid (TfOH, 40 μL, 0.45 mmol) was added to the mixture. A brown color was immediately observed and this mixture was stirred at 80 ºC for 30 min. The color of the solution turned dark brown and formation of a black precipitate was observed. The chloroform supernatant was filtered through short pad of silica that was eluted with 20 mL of additional chloroform. The obtained chloroform solution was concentrated in vacuo and the residue was purified by MPLC (7:3 hexanes:EtOAc elution) to give the rearranged dilactone **5** (68 mg, 0.38 mmol, 37%) as a white crystalline solid along with 3-isochromanone **6** (34 mg, 0.38 mmol, 24%), also as a white crystalline solid.

Data for the dilactone (5)

^{1}H NMR (500 MHz, Chloroform-*d*) δ 6.23–6.07 (nfom, 2*H*), 6.03–5.87 (nfom, 2*H*), 4.75 (dd, *J* = 10.8, 0.7 Hz, 1H, C8H_aH$_b$), 4.25 (d, *J* = 10.9 Hz, 1H, C8H$_a$$H_b$), 3.16 (dd, *J* = 18.1, 0.7 Hz, 1H, C3H$_a$$H_b$), and 2.75 (d, *J* = 18.1 Hz, 1H, C3H_aH$_b$).

^{13}C{^{1}H} NMR (126 MHz, Chloroform-*d*) δ 176.5, 171.7, 125.2, 123.9, 123.3, 122.9, 87.5, 76.1, 51.0, and 38.6.

IR (neat): 1777, 1250, and 736 cm^{-1}.

HRMS (ESI) *m/z*: [M + Na]$^+$ Calcd for $C_{10}H_8NaO_4^+$ for 215.0320; Found 215.0308.

mp: 105–107 °C

3-Isochromanone (6) (NMR data matches well with literature values[3]):

^{1}H NMR (500 MHz, Chloroform-*d*) δ 7.36–7.29 (m, 2H), 7.26–7.21 (m, 2*H*), 5.31 (s, 2H, CH_2O), and 3.71 (s, 2H, CH_2CO).

^{13}C{^{1}H} NMR (126 MHz, Chloroform-*d*) δ 170.8, 131.7, 131.1, 128.9, 127.5, 127.2, 124.8, 70.2 and 36.3.

IR (neat): 1746, 1408, 1250, 1222, 1030, and 775 cm^{-1}.

mp: 80–82 °C (lit.[4] 80–81 °C).

(±)-2-((3a*R*,6*S*)-1-Oxotetrahydro-3*H*-3a,6-epoxyisobenzofuran-7a(1*H*)-yl)acetic acid (9)

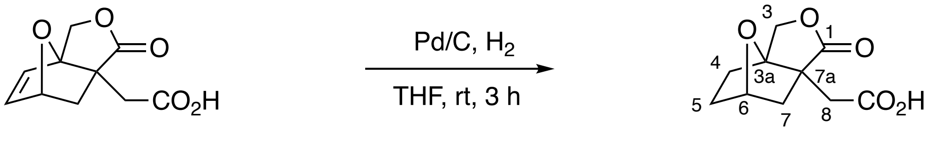

1 *9*

The lactone acid **1** (4.9 g, 23.3 mmol) and Pd/C (10%, 350 mg) were placed in a 250 mL Fisher-Porter tube with a stirring bar. Under a nitrogen atmosphere, THF (100 mL) was added and the reaction vessel was purged with H_2. The reactor headspace was pressurized to ca. 10 psi with H_2 and then the pressure was slowly released. This cycle was repeated twice more. The reactor was then pressurized to 30 psi of H_2 and the mixture was stirred for 3 h at room temperature. The crude reaction mixture was filtered through a small plug of silica (eluant: 10 mL THF). The volatiles were removed under reduced pressure to provide a white solid, which was dried under vacuum to give the lactone acid **9** (4.8 g, 97%) as a white crystalline solid.

^{1}H NMR (500 MHz, Acetone-d_6) δ 4.59 (dd, *J* = 10.3 Hz, 1H, C_3*Ha*Hb), 4.51 (dd, *J* = 5.3, 5.3 Hz, 1H, *H6*), 4.41 (d, *J* = 10.3 Hz, 1H, C3H_a*H_b*), 2.85 (d, *J* = 15.6 Hz, 1H, *H_a*H_b), 2.60 (d, *J* = 15.6 Hz, 1H, C8H_a*H_b*), 2.22 (ddd, *J* = 12.4, 5.0, 2.4 Hz, 1H, C7H_{endo}*H_{exo}*), 2.09–2.01 (m, 1H), 1.89 (dddd, *J* = 12.3, 9.2, 5.6, 2.4 Hz, 1H), 1.82 (d, *J* = 12.4 Hz, 1H, C7*H_{endo}*H_{exo}), and 1.77–1.66 (m, 2H).

^{13}C{^{1}H} NMR (126 MHz, Acetone-d_6) δ 179.7, 172.1, 92.8, 76.9, 69.5, 53.8, 45.6, 39.9, 29.5, and 25.1.

IR (neat): 2978, 1767, 1732, 1134, and 1005 cm^{-1}.

HRMS (ESI) *m/z*: $[M + Na]^+$ Calcd for $C_{10}H_{12}NaO_5^+$ for 235.0582; Found 235.0566.

mp: 163–164 °C.

(±)-4,7-Dihydro-3a,7a-(methanooxymethano)benzofuran-2,10(3*H*)-dione (10b) and (±)-6,7-Dihydro-3a,7a-(methanooxymethano)benzofuran-2,10(3*H*)-dione (10a):

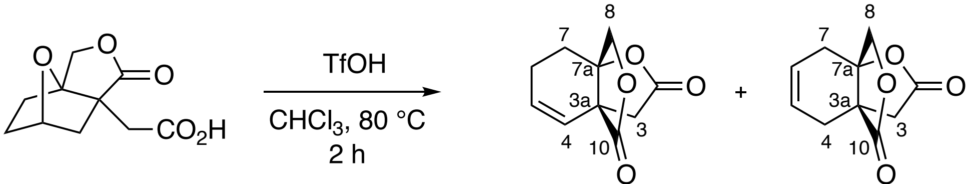

9 ***10a*** ***10b***

The lactone acid **9** (42 mg, 0.2 mmol) was suspended in chloroform (2 mL) in a 5 mL Schlenk flask. Trifluoromethanesulfonic acid (TfOH, 6 µL, 30 mol%) was added. A pale brown color was immediately observed. This solution was stirred for 2 h at 80 ºC. The chloroform was concentrated in vacuo; a ^{1}H NMR spectrum indicated a nearly 1:1 mixture of dilactone products. The residue was purified and partially separated by flash column chromatography on SiO_2 (2:1 hexanes:EtOAc elution) to give the dilactones **10b** (7 mg) as the faster eluting and **10a** (17 mg) as the slower eluting components (24 mg, 62% total yield), each as a colorless oil.

NMR Data for 10a

^{1}H NMR (500 MHz, Chloroform-*d*) δ 6.06 (ddd, *J* = 9.8, 5.7, 2.2 Hz, 1H, *H5*), 5.80 (ddd, *J* = 9.9, 2.8, 1.4 Hz, 1H, *H4*), 4.53 (d, *J* = 11.2, 1H, C8H_aH$_b$), 4.32 (d, *J* = 11.1 Hz, 1H, C8H_aH$_b$), 3.21 (d, *J* = 18.2, 1H, C3H_aH$_b$), 2.68 (d, *J* = 18.3 Hz, 1H, C3H$_a$$H_b$), 2.40 (ddddd, *J* = 18.7, 5.9, 5.9, 2.0, 1.5 Hz, 1H, C6H$_a$$H_b$), 2.27 (ddd, *J* = 14.0, 5.8, 1.9, 1H, C7H_aH$_b$), 2.19-2.11 (ddddd, *J* = 18.7, 11.8, 5.8, 2.4, 2.4 Hz 1H, C6H$_a$$H_b$), and 1.79 (ddd, *J* = 13.8, 11.9, 6.2 Hz, 1H, C7H$_a$$H_b$).

^{13}C{^{1}H} NMR (126 MHz, Chloroform-*d*) δ 176.0, 172.2, 130.2, 122.5, 87.6, 71.4, 49.9, 38.4, 26.2, and 22.8.

NMR Data for 10b

^{1}H NMR (500 MHz, Chloroform-*d*) δ 5.97–5.91 (nfom, 1H, *H5* or *H6*), 5.91–5.87 (nfom, 1H, *H5* or *H6*), 4.59 (d, *J* = 10.9 Hz, 1H, C8H_aH$_b$), 4.20 (d, *J* = 10.8 Hz, 1H, C8H$_a$$H_b$), 3.10 (d, *J* = 18.3 Hz, 1H, C3H_aH$_b$), 2.74 (d, *J* = 18.4 Hz, 1H, C3H$_a$$H_b$), 2.68–2.56 (m, 2H), 2.50 (m, 2H).

^{13}C{^{1}H} NMR (126 MHz, Chloroform-*d*) δ 178.7, 172.4, 125.6, 124.8, 88.0, 75.0, 48.1, 38.2, 31.0, and 29.8.

Data from the mixture:

IR (neat): 2924, 2853, 1770, 1191,1041, and 983 cm^{-1}.

HRMS (ESI) *m/z*: $[M + H]^+$ Calcd for $C_{10}H_{11}O_4^+$ for 195.0657; Found 195.0645.

(±)-2-((1a*R*,2*R*,6a*R*,6b*R*)-4-Oxotetrahydro-6*H*-2,6a-epoxyoxireno[2,3-*e*]isobenzofuran-3a(4*H*)-yl)acetic acid (11)

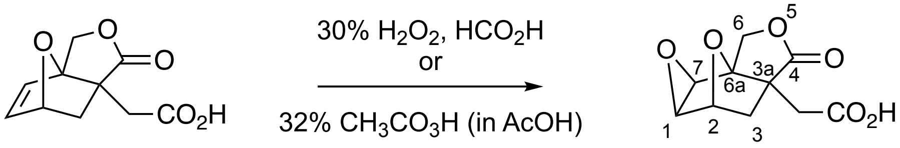

1 ***11***

The lactone acid **1** (2.28 g) was added to a 100 mL round-bottom flask. To this was added 30% H_2O_2 (10 mL) and formic acid (36 mL). The resulting homogenous mixture was place into a preheated (60 °C) oil bath for 2 h. The resulting colorless solution was evaporated under vacuum to obtain a white, sticky, solid material. This solid was recrystallized from ethanol (10 mL) to provide the epoxide of **11** (2.1 g, 86%) as a white crystalline solid.

***Caution**:* On one occasion, a large exotherm was observed following this similar procedure.[5] We do not recommend using this procedure involving the *in situ* generation of the unstable performic acid. Therefore, we developed the following protocol.

A **preferred procedure for this epoxidation** reaction using peroxyacetic acid.

Lactone acid **1** (210 mg, 1 mmol) was added to a 20 mL glass vial. To this was added CH_3CO_3H in AcOH (2 mL, 32% peracid, ca. 8 mmol). The resulting homogenous mixture was stirred for 4 days at ambient temperature. To this colorless solution was added Et_2O (~4 mL) followed by hexanes (~8 mL). A white precipitate appeared. The supernatant liquid phase was decanted the precipitate was washed with a small amount of Et_2O. This solid was recrystallized from ethanol (2 mL) to provide the epoxide of **11** (185 mg, 82%) as a white crystalline solid.

^{1}H NMR (500 MHz, DMSO-*d*6): δ 12.64 (s, 1H, -CO_2*H*), 4.80 (d, *J* = 10.8 Hz, 1H, *H6*), 4.54 (d, *J* = 5.2 Hz, 1H, *H2*), 4.48 (d, *J* = 10.8 Hz, 1H, *H6'*), 3.78 (d, *J* = 3.3 Hz, 1H, *H1* or *H7*), 3.67 (d, *J* = 3.3 Hz, 1H, *H1* or *H7*), 2.68 (d, *J* = 15.3 Hz, 1H, C*H2*CO_2H), 2.63 (d, *J* = 15.3 Hz, 1H, C*H2*CO_2H), 2.16 (dd, *J* = 12.9, 5.2 Hz, 1H, $H3_{exo}$), and 1.83 (d, *J* = 12.9 Hz, 1H, $H3_{endo}$).

^{13}C{^{1}H} NMR (126 MHz, DMSO-d_6) δ 177.1, 171.0, 88.4, 74.6, 67.4, 54.6, 48.6, 46.1, 38.5, and 36.0.

IR (neat): 3083 (br), 1769, 1730, 1004, and 862 cm^{-1}.

HRMS (ESI) *m/z:* $[M + H]^+$ Calcd for $C_{10}H_{11}O_6^+$ for 227.0556; Found 227.0543.

mp: 158–159 °C.

(±)-3-Bromodihydro-2*H*,7*H*,9*H*-2,6a-methanodifuro[3,2-b:3',4'-c]pyran-5,7(6*H*)-dione (12a) and
(±)-10-Bromodihydro-7*H*,9*H*-2,6a:3,9a-dimethanofuro[3,4-*e*][1,4]dioxocine-5,7(6*H*)-dione (12b)

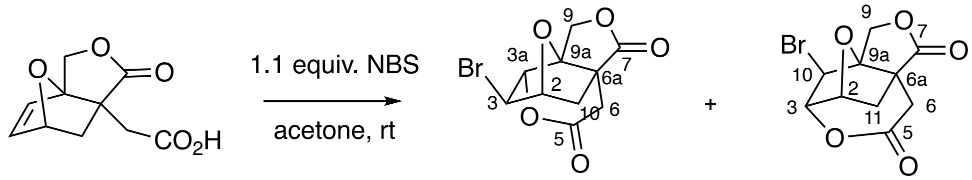

1 ***12a*** (6:1) ***12b***

The lactone acid **1** (210 mg, 1.0 mmol) was dissolved in acetone (5 mL) in a 20 mL Schlenk flask. *N*-Bromosuccinimide (196 mg, 1.1 mmol) was added to the mixture stirred at room temperature for overnight. The reaction mixture was concentrated in vacuo; a proton NMR spectrum of this crude product mixture indicated the presence of an ca. 6:1 mixture of **12a** to **12b**. The residue was purified by MPLC (6:4 hexanes:EtOAc elution) to give a mixture of these largely coeluting bromolactones **12a and 12b** (210 mg, 73%) as a broad melting white crystalline solid.

NMR Data for major bromolactone 12a (extracted from the spectrum of the mixture).

^{1}H NMR (500 MHz, Chloroform-*d*) δ 5.14 (dd, *J* = 1.4, 0.8 Hz, 1H, *H3a*), 4.80 (d, *J* = 11.4 Hz, 1H, *H9*), 4.70 (dd, *J* = 5.5, 0.8 Hz, 1H, *H2*), 4.64 (d, *J* = 11.6 Hz, 1H, *H9'*), 4.03 (d, *J* = 1.6 Hz, 1H, *H3*), 3.18 (d, *J* = 18.8 Hz, 1H, *H6*), 2.83 (dd, *J* = 13.9, 5.7 Hz, 1H, $H10_{exo}$), 2.73 (d, *J* = 18.7 Hz, 1H, *H6'*), and 1.96 (d, *J* = 13.9 Hz, 1H, $H10_{endo}$).

^{13}C{^{1}H} NMR (126 MHz, Chloroform-*d*) δ 175.0 (C7), 163.7 (C5), 85.9 (C3a), 84.9 (9 a), 83.7 (C2), 65.7 (C9), 52.2 (C3), 48.6 (C6a), 40.3 (C10), and 32.6 (C6).

HMBC HSQC

NMR Data for minor bromolactone 12b (extracted from the spectrum of the mixture).

^{1}H NMR (500 MHz, Chloroform-*d*) δ 5.05 (nfom, 1H, *H3*), 4.64 (d, *J* = 11.9 Hz, 1H, H9), 4.55 (s, 1H, H10), 4.47 (d, *J* = 11.9 Hz, 1H, H9'), 3.41 (d, *J* = 18.7 Hz, 1H, *H6*), 3.03 (ddd, *J* = 18.6, 2.3, 1.0 Hz, 1H, *H6'*), 2.45 (dddd, *J* = 12.7, 2.4, 2.4, 1.5 Hz, 1H, $H11_{exo}$), and 2.34 (dd, *J* = 12.7, 1.1 Hz, 1H, $H11_{endo}$). The resonance for H2 could not be definitively identified.

Data for the mixture:

IR (neat): 3010, 1778, 1750, 1707, 1375, 1190, 1062, 1009, and 585 cm^{-1}.

HRMS (ESI) *m/z:* $[M + H]^+$ Calcd for $C_{10}H_{10}BrO_5^+$ for 288.9712; Found 288.9697.

mp: 188–194 °C.

(±)-3-Iododihydro-2*H*,7*H*,9*H*-2,6a-methanodifuro[3,2-b:3',4'-c]pyran-5,7(6*H*)-dione (13a)

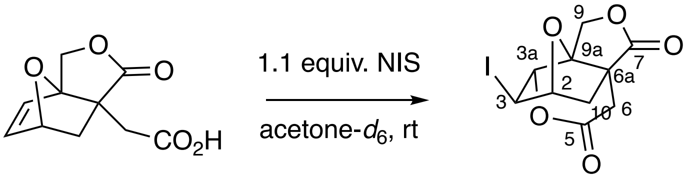

1 ***13a***

The lactone acid **1** (42 mg, 0.20 mmol) *N*-iodosuccinimide (44 mg, 0.22 mmol) were combined in a NMR tube and acetone-d_6 (0.6 mL) was added. The pale brown homogenous mixture was allowed to stand at room temperature. After 4 days NMR analysis indicated ~40% of unreacted starting acid along with unreacted NIS. The reaction mixture was concentrated in vacuo and the residue was purified by MPLC (6:4 hexanes:EtOAc elution) to give a the iodolactone **13a** (35 mg, 52%) as a white crystalline solid. This sample contained ca. 4% of a contaminant with many analogous resonances in the ^{1}H NMR spectrum, although its structure could not be definitively identified as that of an isomeric iodolactone (cf. **12b** vs. **12a**).

NMR Data for iodolactone 13a

^{1}H NMR (500 MHz, Chloroform-*d*) δ 5.25 (dd, *J* = 2.0, 0.6 Hz, 1H, *H3a*), 4.79 (d, *J* = 11.5 Hz, 1H, *H9*), 4.75 (dd, *J* = 5.2, 0.6 Hz, 1H, *H2*), 4.64 (d, *J* = 11.5 Hz, 1H, *H9'*), 3.99 (d, *J* = 2.0 Hz, 1H, *H3*), 3.18 (d, *J* = 18.7 Hz, 1H, *H6*), 2.734 (dd, *J* = 13.8, 5.4 Hz, 1H, $H10_{exo}$), 2.733 (d, *J* = 18.7 Hz, 1H, *H6'*), and 1.99 (d, *J* = 13.9 Hz, 1H, $H10_{endo}$).

^{13}C{^{1}H} NMR (126 MHz, Chloroform-*d*) δ 175.2, 163.9, 87.6, 85.3, 85.2, 65.6, 48.5, 41.5, 32.7, and 24.6.

IR (neat): 3003, 1777, 1749, 1221, 1193, and 1121.

HRMS (ESI) *m/z*: $[M + H]^+$ Calcd for $C_{10}H_{10}IO_5^+$ for 336.9573; Found 336.9558.
mp: 184–186 °C.

(±)-(3a*R*,5*R*,7a*R*)-5-Hydroxy-4,5-dihydro-3a,7a-(methanooxymethano)benzofuran-2,10 (3*H*)-dione (7b)

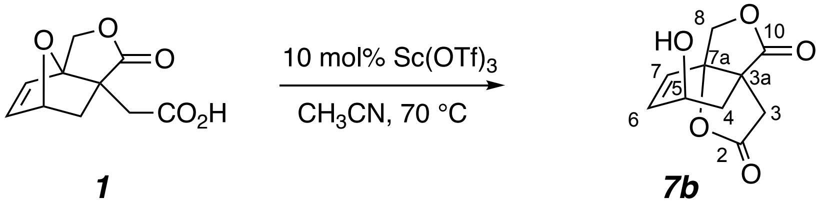

1 ***7b***

The lactone acid **1** (210 mg, 1.0 mmol) and $Sc(OTf)_3$ (49 mg, 0.1 mmol) were combined in a screw-capped culture tube. Acetonitrile (5.0 mL) was added. This mixture was placed in a preheated (70 °C) oil bath and kept there for 6 h. The brownish homogenous mixture was evaporated under vacuum and the crude material was purified by flash column chromatography (hexanes:EtOAc 1:1) to provide a sample of **7b** as a white crystalline solid, which contained an impurity that appeared to be itaconic acid (ca. 7 mol%). This material was recrystallized from ethanol to give (102 mg, 49%) of **7b**.

^{1}H NMR (500 MHz, Acetone-d_6): δ = 6.15 (ddd, *J* = 10.3, 2.2, 1.1 Hz, 1H, *H6*), 5.91 (dd, *J* = 10.3, 2.1 Hz, 1H, *H7*), 4.65 (dd, *J* = 11.1, 0.6 Hz, 1H, *H8*), 4.64 (dddd, *J* = 9.6, 5.0, 2.1, 2.1 Hz, 1H, *H5*), 4.49 (d, *J* = 11.2 Hz, 1H, *H8'*), 3.07 (d, *J* = 18.4 Hz, 1H, *H3*), 2.82 (d, *J* = 18.4 Hz, 1H, *H3'*), 2.36 (ddd, *J* = 13.6, 5.1, 1.2 Hz, 1H, $H4_{eq}$), and 2.21 (dd, *J* = 13.5, 9.4 Hz, 1H, $H4_{ax}$).

^{13}C{^{1}H} NMR (126 MHz, Acetone-d_6): δ 178.2, 172.7, 138.2, 123.1, 86.6, 74.7, 62.8, 49.8, 35.5 and 34.6.

IR (neat): 3439, 1776, 1251, 1061, and 936 cm^{-1}.

HRMS (ESI) *m/z*: $[M + Na]^+$ Calcd for $C_{10}H_9NaO_5^+$ for 233.0426; Found 233.0411.

mp: 144–145 °C.

(±)-(3a*R*,5*S*,7a*R*)-5-Hydroxytetrahydro-3a,7a-(methanooxymethano)benzofuran-2,10(3*H*)-dione (14):

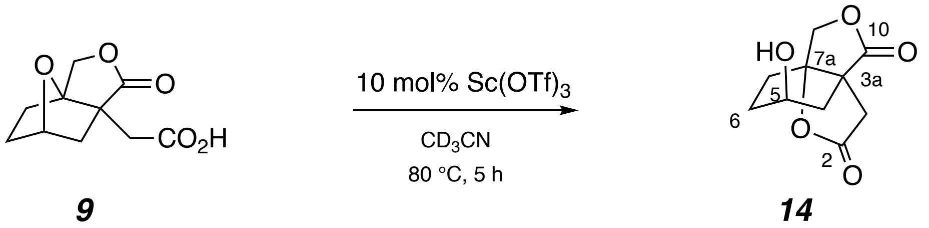

A mixture of $Sc(OTf)_3$ (5 mg, 0.01mmol) and the lactone acid **9** (21 mg, 0.1 mmol) were taken into a screw-capped culture tube. Acetonitrile-d_3 (0.6 mL) was added. The resulting colorless homogenous solution was heated at 80 °C for 5 h. The solvent was evaporated under vacuum from the resulting colorless solution and the crude material was eluted through a silica gel plug with the aid of EtOAc. The eluent was concentrated and gave **14** (17 mg, 80%) as a white crystalline solid. The proton NMR spectrum of this reaction mixture showed a very clean conversion to the set of resonances for the product **14**.

^{1}H NMR (500 MHz, Acetone-d_6): δ 4.64 (d, J = 11.3 Hz, 1H, *H8*), 4.45 (dd, J = 11.3, 0.6 Hz, 1H, *H8'*), 3.97 (dddd, J = 10.5, 10.5, 4.0, 4.0 Hz, 1H, *H5*), 3.09 (d, J = 18.0 Hz, 1H, *H3*), 2.79 (dd, J = 18.0, 0.7 Hz, 1H, *H3'*), 2.9 (br s, 1H, O*H*), 2.29 (ddd, J = 14.2, 4.4, 3.6 Hz, 1H, $H7_{eq}$), 2.23 (ddd, J = 13.8, 4.0, 2.2, Hz, 1H, $H4_{eq}$), 1.98 (ddddd, J = 13.5, 4.6 3.8, 3.8, 2.2 Hz, 1H, $H6_{eq}$), 1.90 (ddd, J = 14.5, 13.4, 4.7 Hz, 1H, $H7_{ax}$), 1.62 (ddd, J = 13.8, 10.6, 0.4 Hz, 1H, $H4_{ax}$), and 1.50 (dddd, J = 13.5, 13.5, 10.2, 4.2 Hz, 1H, $H6_{ax}$).

^{1}H NMR (500 MHz, MeCN-d_3): δ 4.49 (d, J = 11.2 Hz, 1H, *H8*), 4.42 (dd, J = 11.3, 0.5 Hz, *H8'*), 3.80 (dddd, J = 10.3, 10.3, 3.8, 3.8 Hz, 1H, *H5*), 2.92 (d, J = 18.1 Hz, 1H, *H3*), 2.80 (dd, J = 18.1, 0.6 Hz, 1H, *H3'*), 2.22 (ddd, J = 14.5, 4.3, 3.8 Hz, 1H, $H7_{eq}$), 2.15 (ddd, J = 13.8, 3.9, 2.2, Hz, 1H, $H4_{eq}$), 1.91 (ddddd, J = 13.5, 4.8 3.7, 3.7, 2.2 Hz, 1H, $H6_{eq}$), 1.79 (ddd, J = 14.4, 13.3, 4.7 Hz, 1H, $H7_{ax}$), 1.55 (ddd, J = 13.9, 10.5, 0.4 Hz, 1H, $H4_{ax}$), and 1.36 (dddd, J = 13.5, 13.5, 10.1, 4.1 Hz, 1H, $H6_{ax}$).

13C{1H} NMR (126 MHz, Acetone-d6): δ 178.2, 172.0, 87.3, 70.9, 63.7, 49.2, 35.4, 35.2, 30.4, and 27.6.

IR (neat): 3052, 3001, 2952, 2899, 2835, 2228, 1702, 1156, 1113, 1020, 999, 883, and 764 cm^{-1}.

HRMS (ESI) *m/z*: $[M + Na]^+$ Calcd for $C_{10}H_{12}NaO_5^+$ for 235.0582; Found 235.0568.

mp: 116–117 °C.

(±)-3-Hydroxydihydro-2*H*,7*H*,9*H*-2,6a-methanodifuro[3,2-*b*:3',4'-*c*]pyran-5,7(6*H*)-dione (15)

Method A:

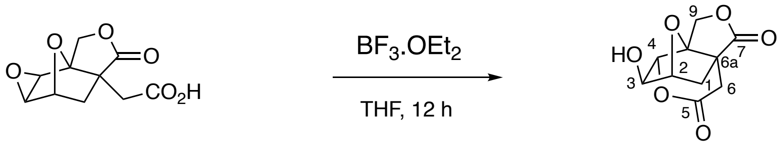

The epoxy acid **11** (1.59 g, 6.2 mmol) was added to a 250 mL round bottom flask. THF (70 mL) was added, followed by $BF_3.OEt_2$ (0.44 mL, 3.5 mmol). This mixture was placed in a preheated (70 °C) oil bath and kept there for 12 h. The resulting colorless solution was evaporated under vacuum to obtain a white sticky solid. This solid was triturated with Et_2O, aided by sonication. The white solid was filtered under vacuum and washed several times with additional fresh Et_2O. The solid was finally recrystallized from ethanol to provide the sample of **15** (1.35 g, 85%) as a white crystalline solid.

Method B:

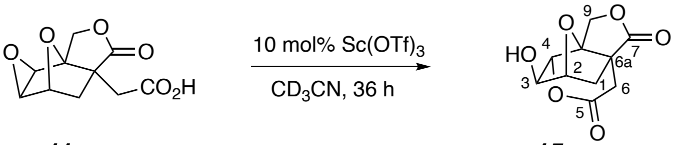

The lactone epoxide **11** (22 mg, 0.1 mmol) and $Sc(OTf)_3$ (4.9 mg, 0.01 mmol) were combined in a screw-capped culture tube. Acetonitrile-d_3 (0.6 mL) was added to the NMR tube. The colorless homogenous mixture was monitored at room temperature. After full consumption of the starting epoxide (36 h), the crude material was passed through a short pad of silica and eluted with 5 mL of EtOAc. The solution was evaporated to provide the white solid material, which was then washed with several mL of Et_2O to give the sample of **15** (19 mg, 84%) as a white crystalline solid.

^{1}H NMR (500 MHz, Acetone-d_6) δ 4.92 (dd, J = 11.4, 0.5 Hz, 1H, *H9β*), 4.66 (d, J = 11.4 Hz, 1H, *H9α*), 4.65 (dd, J = 1.2, 1.2 Hz, 1H, *H3a*), 4.42 (dd, J = 5.8, 1.3 Hz, 1H, *H2*), 4.13 (dd, J = 1.2 Hz, 1H, *H3*), 3.05 (d, J = 18.7 Hz, 1H, *H6α*), 2.94 (dd, J = 18.6, 0.6 Hz, 1H, *H6β*), 2.47 (dd, J = 13.6, 5.9 Hz, 1H, *H1α*), and 2.06 (d, J = 13.7 Hz, 1H, *H1β*).

^{13}C{1H} NMR (126 MHz, Acetone-d_6) δ 177.3 (C7), 165.4 (C5), 85.9 (3a), 84.6 (*9a*), 84.0 (C2), 81.2 (C3), 66.9 (C9), 49.6 (6a), 38.0 (C1), and 32.5 (C6).

Assignments based on: HSQC, HMBC, NOESY correlations.

IR (neat): 3453, 1776, 1745, 1378, 1223, 1065, and 1008 cm^{-1}.

HRMS (ESI) *m/z*: $[M + Na]^+$ Calcd for $C_{10}H_{10}NaO_6^+$ for 249.0375; Found 249.0357.

mp: 196–198 °C.

(±)-(3a*R*,5*R*,7a*R*)-2,10-Dioxo-2,3,4,5-tetrahydro-3a,7a-(methanooxymethano)benzofuran-5-yl acrylate (16a)

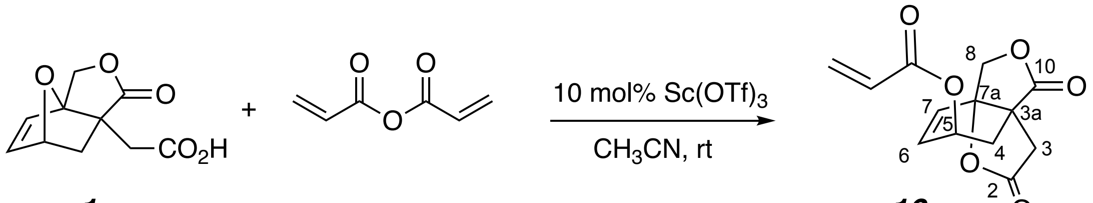

1 **16a**

A mixture of the lactone acid **1** (210 mg, 1.0 mmol) and $Sc(OTf)_3$ (49 mg, 0.1 mmol) were combined in a screw-capped culture tube. Acetonitrile (5.0 mL) and acrylic anhydride (230 mL, 1.95 mmol) were added. The homogenous mixture was stirred at room temperature for 20 h. The solvent was evaporated under vacuum and the crude material was purified by MPLC (hexanes:EtOAc 4:6) to provide the sample of **16a** (176 mg, 67%) as a white crystalline solid.

^{1}H NMR (500 MHz, Chloroform-*d*): δ = 6.42 (dd, *J*= 17.3, 1.5 Hz, 1H, H_ZH_EC=C), 6.20 (ddd, *J* = 10.3, 3.2, 0.8 Hz, 1H, *H6*), 6.10 (dd, *J* = 10.3, 1.6 Hz, 1H, *H7*), 6.09 (dd, *J*= 17.3, 10.5 Hz, 1H, =CHC=O), 5.92 (dd, *J*= 10.5, 1.3 Hz, 1H, H_ZH_EC=C), 5.56 (dddd, *J* = 8.1, 4.7, 3.2, 1.6 Hz, 1H, *H5*), 4.69 (d, *J* = 11.0 Hz, 1H, *H8*), 4.38 (d, *J* = 11.0 Hz, 1H, *H8'*), 3.09 (d, *J* = 18.6 Hz, 1H, *H3*), 2.84 (d, *J* = 18.6 Hz, 1H, *H3'*), 2.40 (ddd, *J* = 13.9, 4.7, 0.8 Hz, 1H, $H4_{eq}$) and 2.21 (dd, *J* = 13.9, 8.1 Hz, 1H, $H4_{ax}$).

^{13}C{^{1}H} NMR (126 MHz, Chloroform-d): δ 176.5, 171.4, 165.1, 132.6, 131.7, 127.5, 126.4, 84.7, 74.9, 64.8, 47.8, 36.2 and 31.5.

IR (neat): 2989, 1781, 1721, 1634, 1189, 1051, and 941 cm^{-1}.

HRMS (ESI) *m/z:* $[M + H]^+$ Calcd for $C_{13}H_{13}O_6^+$ for 265.0712; Found 265.0696.

mp: 122–123 °C.

(±)-(3a*R*,5*R*,7a*R*)-2,10-Dioxo-2,3,4,5-tetrahydro-3a,7a-(methanooxymethano)benzofuran-5-yl methacrylate (16b)

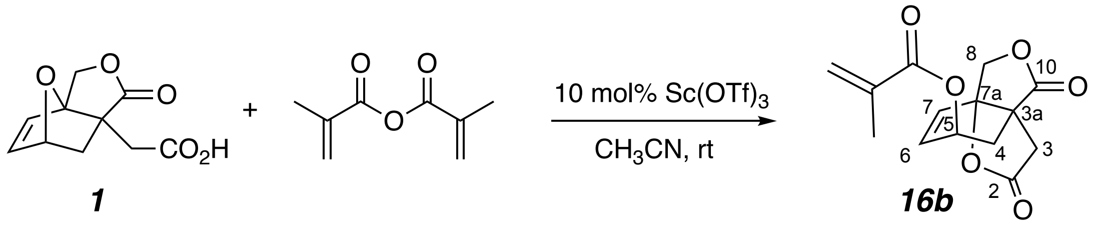

A mixture of the lactone acid **1** (2.2 g, 10.5 mmol) and $Sc(OTf)_3$ (515 mg, 1.05 mmol) were combined in a screw-capped culture tube. Acetonitrile (30 mL) and methacrylic anhydride (2.2 mL, 15.7 mmol) were added. The homogenous mixture was stirred at room temperature for 16 h. The solvent was evaporated under vacuum and the crude material was purified by flash column chromatography (hexanes:EtOAc 4:6) to provide the sample of **16b** (1.9 gm, 65%) as a white crystalline solid.

^{1}H NMR (500 MHz, Chloroform-*d*): δ 6.24 (ddd, *J* = 10.2, 3.4, 0.6 Hz, 1H, *H6*), 6.12 (dd, *J* = 10.3, 1.4 Hz, 1H, *H7*), 6.09 (dq, *J* = 1.1, 1.1 Hz, 1H, H_ZH_EC=C), 5.65 (dq, *J* = 1.5, 1.5 Hz, 1H, H_ZH_EC=C), 5.53 (dddd, *J* = 7.5, 4.8, 3.4, 1.4 Hz, 1H, *H5*), 4.69 (d, *J* = 10.9 Hz, 1H, *H8*), 4.38 (d, *J* = 11.0 Hz, 1H, *H8'*), 3.11 (d, *J* = 18.6 Hz, 1H, *H3*), 2.85 (d, *J* = 18.6 Hz, 1H, *H3'*), 2.35 (ddd, *J* = 14.0, 4.7, 0.6 Hz, 1H, *H4*), 2.29 (dd, *J* = 14.0, 7.5 Hz, 1H, *H4'*), and 1.94 (dd, *J* = 1.7, 1.0 Hz, 3H, -CH_3).

^{13}C{^{1}H} NMR (126 MHz, Chloroform-d): δ 176.6, 171.5, 166.4, 135.5, 131.6, 127.2, 126.7, 84.7, 75.3, 64.8, 47.6, 36.7, 31.8, and 18.3.

IR (neat): 1781, 1713, 1635, and 1168 cm^{-1}.

HRMS (ESI) *m/z*: $[M + H]^+$ Calcd for $C_{14}H_{15}O_6^+$ for 279.0869; Found 279.0851.

mp: 105–106 °C.

(±)-(3aR,5R,7aR)-2,10-Dioxo-2,3,4,5-tetrahydro-3a,7a-(methanooxymethano)benzofuran-5-yl Acetate (16c):

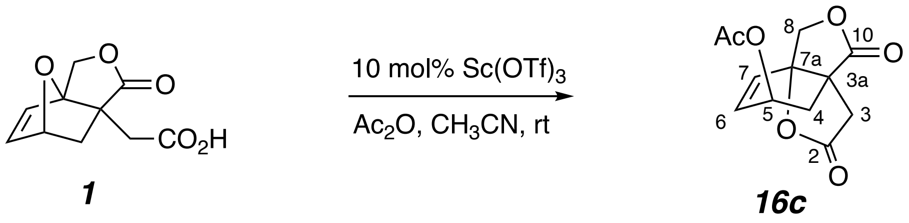

A mixture of $Sc(OTf)_3$ (49 mg, 0.1mmol) and the lactone acid **1** (210 mg, 1 mmol) were taken into a screw-capped culture tube. Acetonitrile (5 mL) and acetic anhydride (190 µL, 2.0 mmol) were added. The dark green-colored homogenous mixture was stirred at room temperature for 16 h. The solvent was evaporated under vacuum and the crude material was purified by flash column chromatography (hexanes:EtOAc, 1:1) to provide the sample of **16c** (130 mg, 51%) as a sticky oil.

^{1}H NMR (500 MHz, **$CDCl_3$**): 6.13 (ddd, *J* = 10.4, 3.0, 0.8 Hz, 1H, *H6*), 6.07 (ddd, *J* = 10.3, 1.6 Hz, 1H, *H7*), 5.49 (dddd, *J* = 8.1, 4.7, 3.0, 1.6 Hz, 1H, *H5*), 4.67 (dd, *J* = 11.0, 0.5 Hz, 1H, *H8*), 4.37 (d, *J* = 11.0 Hz, 1H, *H8'*), 3.06 (d, *J* = 18.6 Hz, 1H, *H3*), 2.82 (d, *J* = 18.5 Hz, 1H, *H3'*), 2.38 (ddd, *J* = 14.0, 4.8, 0.8 Hz, 1H, *H4*), 2.12 (dd, *J* = 14.0, 8.3 Hz, 1H, *H4'*), and 2.07 (s, 3H).

Assignments based on HMBC HSQC correlations.

^{13}C{1H} NMR (126 MHz, **$CDCl_3$**) δ 176.5 (*C*10), 171.4 (*C*2), 170.1 (CH_3CO_2), 131.8 (*C6*), 126.1 (*C*7), 84.7 (*C*7a), 74.7 (*C*8), 64.6 (*C*5), 47.9 (*C*3a), 36.0 (*C*3), 31.2 (*C*4), and 20.9 (CH_3CO_2).

IR (neat): 3023, 2937, 1775, 1732, 1373, 1224, 1009, and 938 cm^{-1}.

HRMS (ESI) *m/z*: $[M + Na]^+$ Calcd for $C_{12}H_{12}NaO_6^+$ for 275.0532; Found 275.0515.

(3a*R*,5*S*,7a*R*)-2,10-Dioxohexahydro-3a,7a-(methanooxymethano)benzofuran-5-yl acrylate (17a)

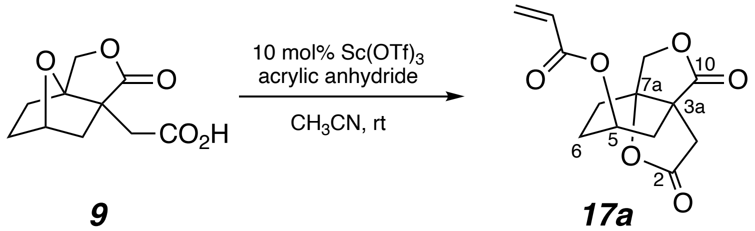

9 ***17a***

A mixture of $Sc(OTf)_3$ (38 mg, 0.094 mmol) and the lactone acid **9** (200 mg, 0.94 mmol) was taken into a screw-capped culture tube. Acetonitrile (2 mL) was added followed by acrylic anhydride (178 μL 1.9 mmol). The colorless homogeneous mixture was stirred at room temperature for 16 h. The solvent was evaporated under vacuum and the crude material was purified by flash column chromatography (hexanes:EtOAc, 2:3) to provide the sample of **17a** (186 mg, 0.70 mmol 75%) as a white crystalline solid.

^{1}H NMR (500 MHz, Acetone-d_6): δ = 6.35 (dd, *J*= 17.2, 1.5 Hz, 1H, H_ZH_EC=C), 6.11 (dd, *J*= 17.2, 10.5 Hz, 1H, CHCOO), 5.90 (dd, *J*= 10.5, 1.5 Hz, 1H, H_ZH_EC=C), 5.11 (dddd, *J*= 9.9, 9.9, 3.9, 3.9, 1H, H5), 4.70 (d, *J* = 11.3 Hz, 1H, *H8*),, 4.50 (d, *J* = 11.3, 0.5 Hz, 1H, *H8'*), 3.20 (d, *J* =18.0 Hz, 1H, *H3*) , 2.89 (dd, *J* =18.0, 0.6 Hz, 1H, *H3'*), 2.39–2.34 (nfom, 1H, $H6^{exo}$), 2.13–2.06 (two nfom, 2H, $H6^{endo}$ and *H7*), 2.34 (ddd, *J* = 13.8, 4.0, 1.8 Hz, 1H, $H4^{exo}$), 1.95 (ddd, *J* = 13.9, 9.9, 0.4 Hz, 1H, $H4^{endo}$), and 1.84–1.70 (nfom, 1H, *H7'*).

^{13}C NMR (126 MHz, Acetone-d_6): δ = 178.3, 172.6, 165.5, 131.6, 129.3, 87.5, 72.2, 68.2, 49.3, 36.4, 32.5, 27.8, and 27.2.

IR (neat): 2949, 2874, 1779, 1719, 1409, 1190, and 764 cm^{-1}.

HRMS (ESI) *m/z:* $[M + H]^+$ Calcd for $C_{13}H_{15}O_6^+$ for 267.0869; Found 267.0851.

mp: 138–140 °C.

(±)-(3a*R*,5*S*,7a*R*)-2,10-Dioxohexahydro-3a,7a-(methanooxymethano)benzofuran-5-yl acetate (17c)

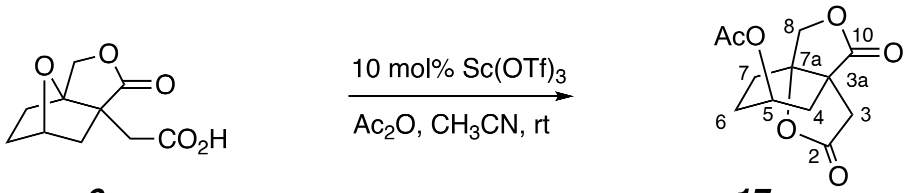

9 ***17c***

A mixture of Sc(OTf)$_3$ (215 mg, 0.47 mmol) and the lactone acid **9** (1 g, 4.7 mmol) were taken into a screw-capped culture tube. Acetonitrile (8 mL) followed by acetic anhydride (1.1 mL, 9.4 mmol) was added. The colorless homogenous mixture was stirred at room temperature for 16 h. The solvent was evaporated under vacuum and the crude material was purified by flash column chromatography (hexanes:EtOAc, 4:6) to provide the sample of **17c** (980 mg, 82%) as a white crystalline solid.

^{1}H NMR (500 MHz, **CDCl$_3$**): 4.91 (dddd, *J* = 9.3, 8.9, 3.9, 3.9 Hz, 1H, *H5*), 4.48 (dd, *J* = 11.1, 0.7 Hz, 1H, *H8*), 4.38 (d, *J* = 11.0 Hz, 1H, *H8'*), 3.03 (dd, *J* = 18.3, 0.7 Hz, 1H, *H3*), 2.78 (d, *J* = 18.3 Hz, 1H, *H3'*), 2.28 (ddd, *J* = 14.9, 4.7, 4.4 Hz, 1H, *H7*), 2.21 (ddd, *J* = 14.3, 3.9, 1.9 Hz, 1H, *H4*), 2.08 (ddddd, *J* = 14.0, 4.9, 4.9, 3.9, 1.9 Hz, 1H, *H6*), 2.03 (s, 3H, COC*H$_3$*), 1.90 (dd, *J* = 14.1, 9.2 Hz, 1H, *H4'*), 1.89 (dd, *J* = 14.9, 12.1, 5.0 Hz, 1H, *H7'*), and 1.54 (dddd, *J* = 13.9, 11.9, 8.8, 4.5 Hz, 1H, *H6'*).

^{13}C{1H} NMR (126 MHz, **CDCl$_3$**) δ 176.8 (C10), 171.6 (C2), 170.1 (CH$_3$*C*O), 86.2 (C7a), 71.9 (C8), 66.5 (C5), 48.0 (C3a), 36.4 (C3), 32.5 (C4), 27.7 (C7), 26.6 (C6), and 21.1 (*C*H$_3$CO).

Assignments based on HMBC HSQC correlations.

IR (neat): 1780, 1750, 1727, 1185, and 1063 cm^{-1}.

HRMS (ESI) *m/z:* [M + Na]$^+$ Calcd for $C_{12}H_{14}NaO_6^+$ for 277.0688; Found 277.0672.

mp: 125–127 °C.

In a separate experiment, this transformation was monitored over time by in situ ^{1}H NMR spectroscopy in CD$_3$CN at ambient temperature. Sc(OTf)$_3$ (10 mol%), Ac$_2$O (18 uL), and acid **1** were used. The reaction was very selective for formation of the acetate **17c**. A copy of the spectrum of the reaction mixture sample is included at the end of this SI. The following spectral data were taken from a NMR sample of purified **17c** in CD$_3$CN.

^{1}H NMR (500 MHz, **CD$_3$CN**): 4.91 (dddd, *J* = 9.8, 9.8, 4.0, 4.0 Hz, 1H, *H5*), 4.51 (d, *J* = 11.2 Hz, 1H, *H8*), 4.38 (dd, *J* = 11.4, 0.8 Hz, 1H, *H8'*), 2.96 (d, *J* = 18.2 Hz, 1H, *H3*), 2.78 (dd, *J* = 18.3, 0.8 Hz, 1H, *H3'*), 2.26 (ddd, *J* = 14.6, 4.2, 4.2 Hz, 1H, *H7*), 2.20 (ddd, *J* = 14.0, 4.0, 2.0 Hz, 1H, *H4*), 2.00 (ddddd, *J* = 13.5, 4.9, 4.2, 4.2 1.9 Hz, 1H, *H6*), 1.97 (s, 3H, COC*H$_3$*), 1.91 (ddd, *J* = 14.7, 12.6, 4.9 Hz, 1H, *H7'*), 1.83 (dd, *J* = 14.0, 10.0 Hz, 1H, *H4'*), and 1.54 (dddd, *J* = 13.7, 12.7, 9.5, 4.3 Hz, 1H, *H6'*).

(±)-(3a*R*,5*S*,7a*R*)-2,10-Dioxohexahydro-3a,7a-(methanooxymethano)benzofuran-5-yl methacrylate (17b)

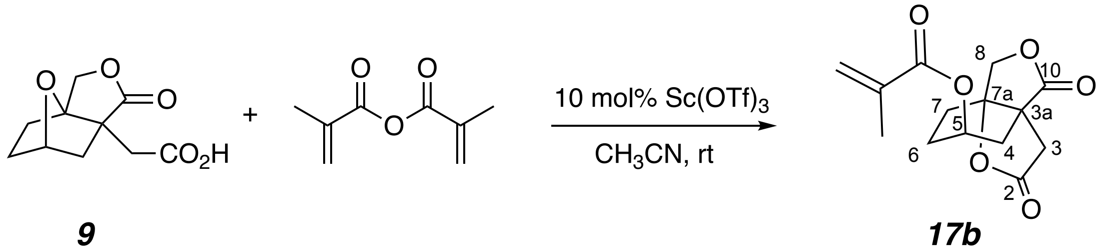

A mixture of the lactone acid **9** (5.0 g, 23.6 mmol) and $Sc(OTf)_3$ (580 mg, 1.18 mmol) were combined in a screw-capped culture tube. Acetonitrile (50 mL) and methacrylic anhydride (5.3 mL, 35.3 mmol) were added. The homogenous mixture was stirred at room temperature for 16 h. The solvent was evaporated under vacuum and the crude material was purified by flash column chromatography (hexanes:EtOAc 4:6) to provide the sample of **17b** (5.1 gm, 76%) as a white crystalline solid.

^{1}H NMR (500 MHz, Chloroform-d): 6.08 (dq, *J* = 1.5, 1.0 Hz, 1H, H_ZH_EC=C), 5.61 (dq, *J* = 1.5, 1.5 Hz, 1H, H_ZH_EC=C), 5.01 (dddd, *J* = 8.7, 8.7, 3.9, 3.9 Hz, 1H, *H5*), 4.49 (dd, *J* = 11.0, 0.4 Hz, 1H, *H8*), 4.40 (d, *J* = 11.0 Hz, 1H, *H8'*), 3.06 (dd, *J* = 18.3, 0.6 Hz, 1H, *H3*), 2.81 (d, *J* = 18.3 Hz, 1H, *H3'*), 2.29 (ddd, *J* = 14.8, 5.5, 4.5 Hz, 1H, $H7_{eq}$), 2.24 (ddd, *J* = 14.4, 3.8, 1.6 Hz, 1H, $H4_{eq}$), 2.13 (ddddd, *J* = 14.1, 5.2, 5.1, 4.1, 1.6 Hz, 1H, $H6_{eq}$), 2.03 (dd, *J* = 14.3, 8.8 Hz, 1H, $H4_{ax}$), 1.96 (ddd, *J* = 14.9, 11.5, 4.8 Hz, 1H, $H7_{ax}$), 1.92 (dd, *J* = 1.0, 1.5 Hz, 3H, -CH_3), and 1.63 (dddd, *J* = 14.2, 11.5, 8.5, 4.5 Hz, 1H, *H6'*).

^{13}C{^{1}H} NMR (126 MHz, Chloroform-d): δ 176.7 (C10), 171.7 (C2), 166.4 (=C*C*=O), 135.8 (=*C*C=O), 126.7 (=CH_2), 86.2 (C7a), 72.1 (C8), 66.8 (C5), 47.9 (C3a), 36.7 (C3), 32.7 (C4), 27.6 (C7), 26.4 (C6), and 18.3 (CH_3).

Assignments based on HMBC HSQC correlations.

IR (neat): 2957, 1781, 1714, 1635, and 1168 cm^{-1}.

HRMS (ESI) *m/z*: [M + H]$^+$ Calcd for $C_{14}H_{17}O_6^+$ for 281.1025; Found 281.1006.

mp: 155–157 °C.

(±)-(3aR,5S,7aR)-2,10-Dioxohexahydro-3a,7a-(methanooxymethano)benzofuran-5-yl Benzoate (17d):

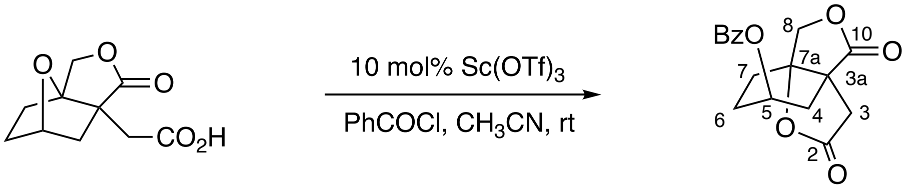

9 ***17d***

A mixture of $Sc(OTf)_3$ (25 mg, 0.05 mmol) and the lactone acid **9** (106 mg, 0.5 mmol) were taken into a screw-capped culture tube. Acetonitrile (3 mL) and benzoyl chloride (116 µL, 1.0 mmol) were added. The colorless homogenous mixture was stirred at room temperature for 16 h. The solvent was evaporated under vacuum and the crude material was purified by flash column chromatography (hexanes:EtOAc, 1:1) to provide the **17d** (128 mg, 81%) as a white crystalline solid.

^{1}H NMR (500 MHz, **$CDCl_3$**): 7.98 (nfom, 2H, *$Ph_{o+o'}$*), 7.63 (tt, *J* = 7.5, 1.4 Hz, 1H, *Ph_p*), 7.42 (nfom, 2H, *$Ph_{m+m'}$*), 5.19 (dddd, *J* = 9.0, 9.0, 3.9, 3.9 Hz, 1H, *H5*), 4.52 (dd, *J* = 11.0, 0.6 Hz, 1H, *H8*), 4.44 (d, *J* = 11.0 Hz, 1H, *H8'*), 3.09 (dd, *J* = 18.2, 0.6 Hz, 1H, *H3*), 2.78 (d, *J* = 18.3 Hz, 1H, *H3'*), 2.36 (ddd, *J* = 14.3, 4.0, 1.8 Hz, 1H, *$H4_{eq}$*), 2.33 (ddd, *J* = 14.9, 5.1, 4.4 Hz, 1H, *$H7_{eq}$*), 2.23 (ddddd, *J* = 14.0, 4.9, 4.9, 3.8, 1.8 Hz, 1H, *$H6_{eq}$*), 2.10 (dd, *J* = 14.3, 9.1 Hz, 1H, *$H4_{ax}$*), 2.0 (ddd, *J* = 14.9, 11.9, 4.9 Hz, 1H, *$H7_{ax}$*), and 1.72 (dddd, *J* = 14.1, 11.8, 8.7, 4.4 Hz, 1H, *$H6_{ax}$*).

^{13}C{1H} NMR (126 MHz, **$CDCl_3$**) δ 176.8, 171.7, 165.7, 133.6, 129.8, 129.5, 128.7, 86.3, 72.0, 67.2, 48.0, 36.6, 32.8, 27.7 and 26.7.

IR (neat): 1780, 1714, 1274, 1113, and 714 cm^{-1}.

HRMS (ESI) *m/z*: $[M + Na]^+$ Calcd for $C_{17}H_{16}NaO_6^+$ for 339.0845; Found 339.0823.

mp: 143–144 °C.

(±)-(3aR,5S,7aR)-2,10-Dioxohexahydro-3a,7a-(methanooxymethano)benzofuran-5-yl Pivalate (17e):

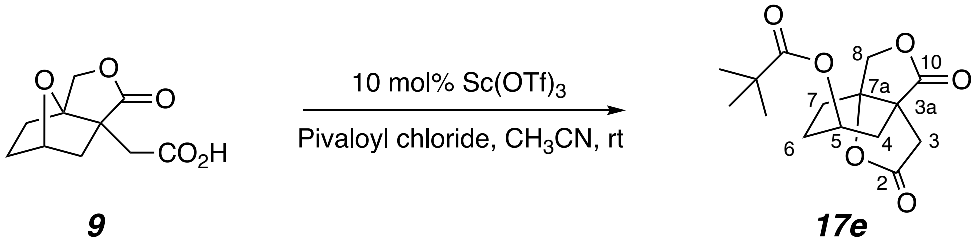

A mixture of $Sc(OTf)_3$ (25 mg, 0.05 mmol) and the lactone acid **9** (106 mg, 0.5 mmol) were taken into a screw-capped culture tube. Acetonitrile (3 mL) and pivaloyl chloride (123 µL, 1.0 mmol) were added. The colorless homogenous mixture was stirred at room temperature for 20 h. The solvent was evaporated under vacuum and the crude material was purified by flash column chromatography (hexanes:EtOAc, 1:1) to provide the pivalate **17e** (105 mg, 71 %) as a white crystalline solid.

^{1}H NMR (500 MHz, **$CDCl_3$**): δ 5.0 (dddd, *J* = 9.3, 9.3, 4.0, 4.0 Hz, 1H, *H5*), 4.68 (dd, *J* = 11.2, 0.5 Hz, 1H, *H8*), 4.50 (d, *J* = 11.2 Hz, 1H, *H8'*), 3.13 (dd, *J* = 18.1, 0.5 Hz, 1H, *H3*), 2.90 (d, *J* = 18.1 Hz, 1H, *H3'*), 2.32 (ddd, *J* = 14.2, 4.5, 1.9 Hz, 1H, $H4_{eq}$), 2.25 (ddd, *J* = 14.0, 4.0, 1.8 Hz, 1H, $H7_{eq}$), 2.08 (dd, *J* = 14.4, 12.1, 4.7 Hz, 1H, $H7_{ax}$), 2.03 (dddd, *J* = 13.7, 4.9, 3.9, 1.9 Hz, 1H, $H6_{eq}$), 1.95 (dd, *J* = 14.1, 9.5 Hz, 1H, $H4_{ax}$), and 1.70 (dddd, *J* = 13.7, 11.9, 9.1, 5.3 Hz, 1H, $H6_{ax}$).

^{13}C{1H} NMR (126 MHz, **$CDCl_3$**) δ 177.3, 176.7, 171.8, 86.5, 71.5, 66.7, 48.1, 38.3, 35.9, 31.7, 26.8, 26.4, and 26.1.

IR (neat): 2971, 1780, 1721, 1281, and 1154 cm^{-1}.

HRMS (ESI) *m/z:* $[M + Na]^+$ Calcd for $C_{15}H_{20}NaO_6^+$ for 319.1158; Found 319.1133.

mp: 86–88 °C.

(±)-5,7-Dioxotetrahydro-2*H*,7*H*,9*H*-2,6a-methanodifuro[3,2-b:3',4'-c]pyran-3-yl acrylate (18a)

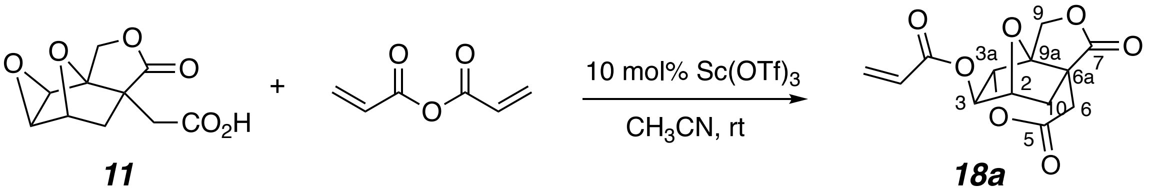

11 **18a**

A mixture of the lactone epoxide **11** (252 mg, 2 mmol) and $Sc(OTf)_3$ (98 mg, 0.2 mmol) was placed into a screw-capped culture tube. Acetonitrile (8 mL) and acrylic anhydride (400 µL, 3.4 mmol) were added. The homogenous mixture was stirred at room temperature for 12 h. The solvent was evaporated under vacuum and the crude material was purified by flash column chromatography (hexanes:EtOAc 4:6) to provide **18a** (354 mg, 63%) as a white crystalline solid.

^{1}H NMR (500 MHz, Acetone-d_6) δ = 6.43 (dd, *J*= 17.3, 1.5 Hz, 1H, H_Z*H_E*C=C), 6.21 (dd, *J*= 17.3, 10.4 Hz, 1H, CHCOO), 5.98 (dd, *J*= 10.5, 1.5 Hz, 1H, *H_Z*H_EC=C), 5.12 (d, *J* = 1.4 Hz, 1H, *H3a*), 5.00 (d, *J* = 11.6 Hz, 1H, *H9*), 4.98 (dd, *J* = 1.3, 1.3 Hz, 1H, *H3*), 4.72 (d, *J* = 11.5 Hz, 1H, *H9'*), 4.67 (dd, *J* = 5.8, 1.2 Hz, 1H, *H2*), 3.17 (d, *J* = 18.8 Hz, 1H, *H6*), 3.01 (d, *J* = 18.8 Hz, 1H, *H6'*), 2.59 (dd, *J* = 13.8, 6.0 Hz, 1H, $H10_{exo}$), and 2.34 (d, *J* = 13.8 Hz, 1H, $H10_{endo}$).

^{13}C{^{1}H} NMR (126 MHz, Acetone-d_6) δ 177.0, 165.6, 165.0, 132.6, 128.6, 85.0, 82.7, 82.6, 81.7, 66.7, 49.6, 37.8, and 32.3.

IR (neat): 1779, 1749, 1725, 1633, 1184, and 1063 cm^{-1}.

HRMS (ESI) *m/z*: $[M + H]^+$ Calcd for $C_{13}H_{13}O_7^+$ for 281.0661; Found 281.0645.

mp: 197–199 °C.

(±)-(5,7-Dioxotetrahydro-2*H*,7*H*,9*H*-2,6a-methanodifuro[3,2-*b*:3',4'-*c*]pyran-3-yl methacrylate (18b)

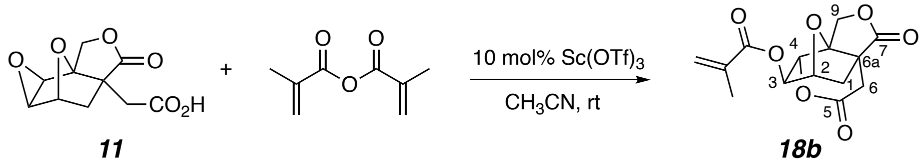

11 ***18b***

Smaller scale (with chromatography): A mixture of lactone epoxide **11** (226 mg, 1 mmol) and $Sc(OTf)_3$ (49 mg, 0.1 mmol) was placed into a screw-capped culture tube. Acetonitrile (6 mL) and methacrylic anhydride (220 µL, 1.5 mmol) were added. The homogenous mixture was stirred at room temperature for 12 h. The solvent was evaporated under vacuum and the crude material was purified by flash column chromatography (hexanes:EtOAc 4:6) to provide the sample of **18b** (220 mg, 75%) as a white crystalline solid.

Larger scale (with recrystallization): A mixture of the lactone epoxide **11** (4.0 g, 17.7 mmol) and $Sc(OTf)_3$ (712 mg, 1.8 mmol) was placed into a screw-capped culture tube. Acetonitrile (120 mL) and methacrylic anhydride (4.0 mL, 35.4 mmol) were added. The homogenous mixture was stirred at room temperature for 12 h. The solvent was evaporated under vacuum to obtain a white solid. This solid was washed several times with Et_2O (these washing contained largely methacrylic acid and anhydride; ^{1}H NMR). The solid was recrystallized from hexane and methanol to provide the sample of **18b** (4.3 g, 82%) as a white crystalline solid.

^{1}H NMR (500 MHz, Acetone-d_6) δ 6.14 (dq, J = 1.5, 1.1 Hz, 1H, H_ZH_EC=C), 5.72 (dq, J = 1.6, 1.6 Hz, 1H, H_ZH_EC=C), 5.09 (d, J = 1.4 Hz, 1H, *H4*), 5.00 (d, J = 11.5 Hz, 1H, *H9*), 4.98 (dd, J = 1.3, 1.3 Hz, 1H, *H3*), 4.72 (d, J = 11.5 Hz, 1H, *H9'*), 4.67 (dd, J = 5.9, 1.3 Hz, 1H, *H2*), 3.18 (d, J = 18.8 Hz, 1H, *H6*), 3.01 (d, J = 18.8 Hz, 1H, *H6'*), 2.59 (dd, J = 13.8, 5.9 Hz, 1H, $H1_{exo}$), 2.34 (d, J = 13.7 Hz, 1H, $H1_{endo}$), and 1.93 (dd, J = 1.6, 1.0 Hz, 3H, -CH_3).

^{13}C{^{1}H} NMR (126 MHz, Acetone-d_6) δ 177.0, 166.8, 165.0, 136.7, 126.8, 85.0, 82.8, 82.6, 81.7, 66.7, 49.6, 37.9, 32.3, and 18.2.

IR (neat): 3052, 3001, 2952, 2899, 2835, 1783, 1719, 1702, 1156, 1113, 1020, 999, 883, and 764 cm^{-1}.

HRMS (ESI) *m/z:* $[M + H]^+$ Calcd for $C_{14}H_{15}O_7^+$ for 295.0818; Found 295.0800.

mp: 169–170 °C.

(±)-5,7-Dioxotetrahydro-2*H*,7*H*,9*H*-2,6a-methanodifuro[3,2-*b*:3',4'-*c*]pyran-3-yl acetate (18c)

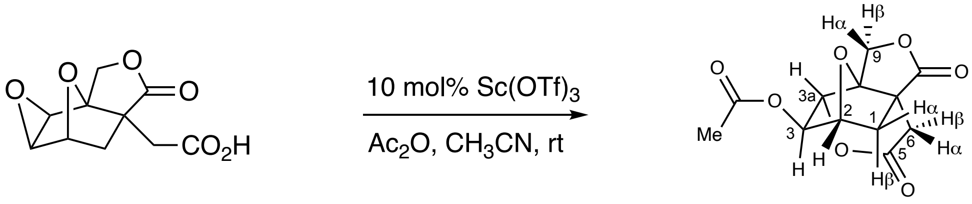

11 ***18c***

A mixture of $Sc(OTf)_3$ (36 mg, 0.07 mmol) and epoxy lactone acid **11** (163 mg, 0.73 mmol) were combined in a screw-capped culture tube. Acetonitrile (1.5 mL) and acetic anhydride (140 µL, 1.48 mmol) were added. The colorless homogenous mixture was stirred at room temperature for 16 h. The solvent was evaporated under vacuum and the crude material was purified by flash column chromatography (hexanes:EtOAc, 4:6) to provide the sample of **18c** (165 mg, 84%) as a white crystalline solid.

^{1}H NMR (500 MHz, Acetone-d_6) δ 5.01 (d, J = 1.4 Hz, 1H, *H3*), 4.98 (d, J = 11.5 Hz, 1H, *H9β*), 4.89 (dd, J = 1.3, 1.3 Hz, 1H, *H3a*), 4.70 (d, J = 11.6 Hz, 1H, *H9α*), 4.60 (dd, J = 5.9, 1.3 Hz, 1H, *H2*), 3.15 (d, J = 18.8 Hz, 1H, *H6α*), 3.00 (d, J = 18.8 Hz, 1H, *H6β*), 2.56 (dd, J = 13.7, 5.9 Hz, 1H, *H1α*), 2.30 (d, J = 13.7 Hz, 1H, *H1β*), and 2.08 (s, 3H).

^{13}C{1H} NMR (126 MHz, Acetone-d_6) δ 177.0 (C7), 170.4 (Me*C*O), 165.0(C5), 84.9 (9a), 82.7(C3a), 82.4 (C3), 81.8 (C2), 66.7 (C9), 49.6 (6a), 37.9 (C1), 32.3 (C6), and 20.7 (CH_3).

Assignments based on: HSQC, HMBC, NOESY correlations.

IR (neat): 2990, 1780, 1750, 1374, and 1237 cm^{-1}.

HRMS (ESI) *m/z:* $[M + Na]^+$ Calcd for $C_{12}H_{12}NaO_7^+$ for 291.0481; Found 291.0462.

mp: 197–198 °C.

Summary of selected HMBC and NOESY data as well as side-by-side listing of ^{13}C and ^{1}H NMR chemical shifts and proton-proton coupling constants (Table S1).

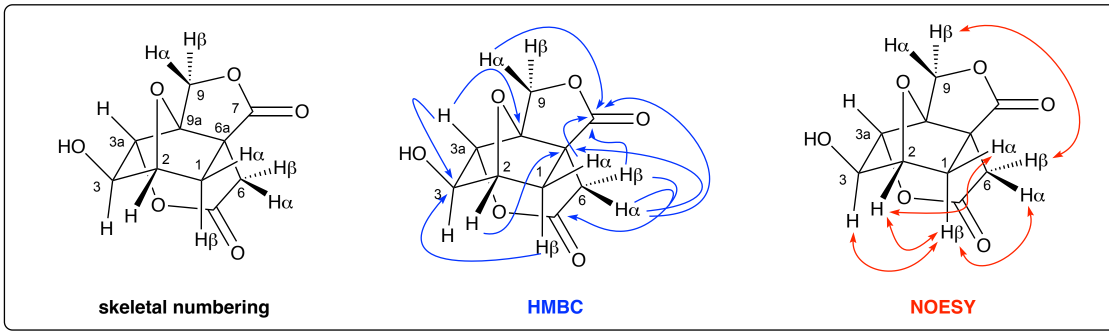

15

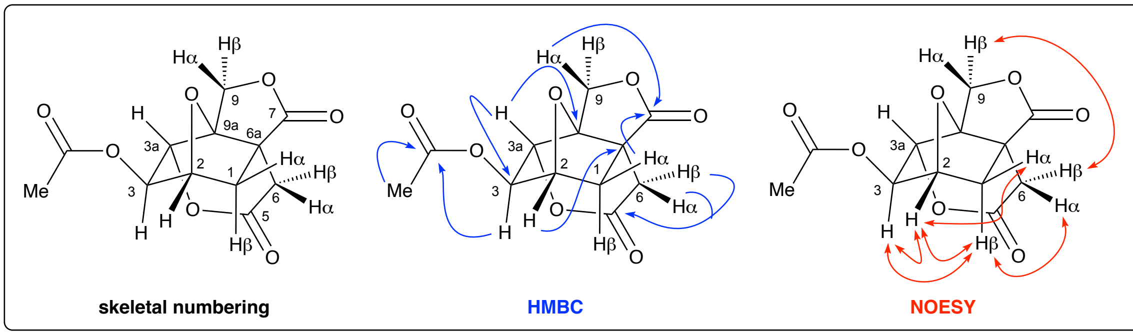

18c

Table S1. 1D ^{13}C and ^{1}H NMR data for the alcohol 15 and the acetate ester 18c

Atom #	$\delta_{13C(OH)}$	$\delta_{13C(OAc)}$	$\delta_{1H(OH)}$	Js$_{(OH)}$ (Hz)	$\delta_{1H(OAc)}$	Js$_{(OAc)}$ (Hz)
1α	38.0	37.9	2.47	13.6, 5.9	2.56	13.7, 5.9
1β	"	"	2.06	13.7	2.30	13.7
2	84.0	81.8	4.42	5.8, 1.3	4.60	5.9, 1.3
3	81.2	82.4	4.13	1.2	5.01	1.4
3a	85.9	82.7	4.65	1.2, 1.2	4.89	1.3, 1.3
5	165.4	165.0	-	-	-	-
6α	32.5	32.3	3.05	18.7	3.15	18.8
6β	"	"	2.94	18.6, 0.6	3.00	18.8, 0.5
6a	49.6	49.6	-	-	-	-
7	177.3	177.0	-	-	-	-
9α	66.9	66.7	4.66	11.4	4.70	11.6
9β	"	"	4.92	11.4, 0.5	4.98	11.5, 0.5
9a	84.6	84.9	-	-	-	-
$H_3\underline{C}CO_2$	-	20.7	-	-	2.08	-
$H_3C\underline{C}O_2$	-	170.4	-	-	-	-

Carbon δs from HSQC, HMBC; Proton δs assignments supported by NOESY with protons in addition to their geminal partners

Supplementary Material References

1 Hoye, T. R.; Hanson, P. R.; Vyvyan, J. R. A practical guide to first-order multiplet analysis in ^{1}H NMR spectroscopy. *J. Org. Chem*. **1994**, *59*, 4096–4103.

2 Hoye, T. R.; Zhao, H. A method for easily determining coupling constant values: an addendum to "a practical guide to first-order multiplet analysis in ^{1}H NMR spectroscopy". *J. Org. Chem*. **2002**, *67*, 4014–4016.

3 Sim, J.; Ryou, B.; Choi, M.; Lee, C.; Park, C.-M. Electro-chemical C(sp3)-H functionalization of γ-lactams based on hydrogen atom transfer. *Org. Lett*. **2022**, *24*, 4264−4269.

4 Bertolini, V.; Appiani, R.; Pallavicini, M.; Bolchi, C. Green oxidation of ketones to lactones with oxone in water. *J. Org. Chem*. **2021**, *86*, 15712−15716.

5 Santacesaria, E.; Russo, V.; Tesser, R.; Turco, R.; Di Serio, M. Kinetics of performic acid synthesis and decomposition. *Ind. Eng. Chem. Res*. **2017**, *56*, 12940–12952.

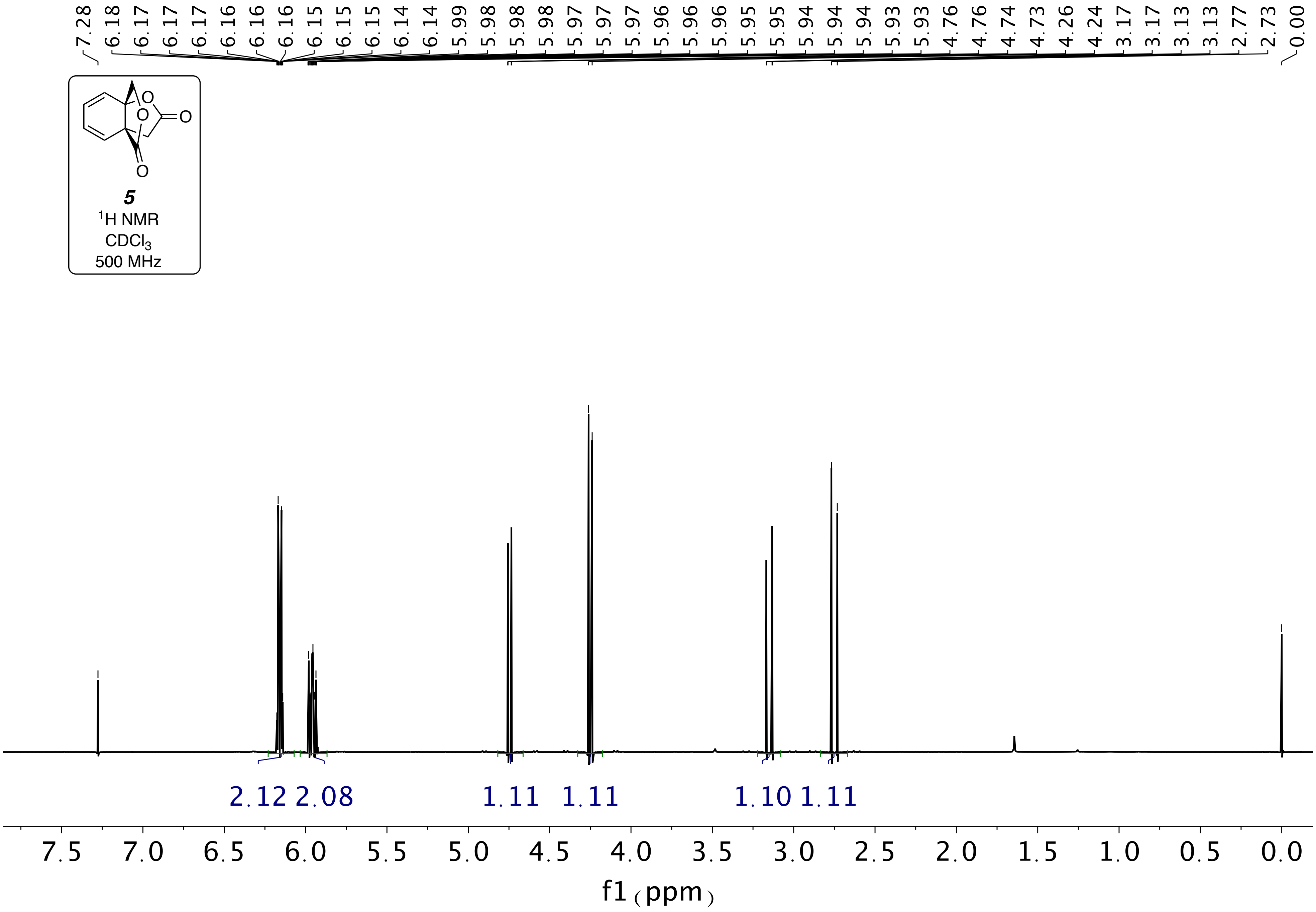
7.28
6.18
6.17
6.17
6.17
6.16
6.16
6.16
6.15
6.15
6.15
6.14
6.14
5.99
5.98
5.98
5.98
5.97
5.97
5.97
5.96
5.96
5.96
5.95
5.95
5.94
5.94
5.94
5.93
5.93
4.76
4.76
4.74
4.73
4.26
4.24
3.17
3.17
3.13
3.13
2.77
2.73
0.00
O
O
O
O
5
^{1}H NMR
$CDCl_3$
500 MHz
2.12
2.08
1.11
1.11
1.10
1.11
7.5
7.0
6.5
6.0
5.5
5.0
4.5
4.0
3.5
3.0
2.5
2.0
1.5
1.0
0.5
0.0
f1 (ppm)

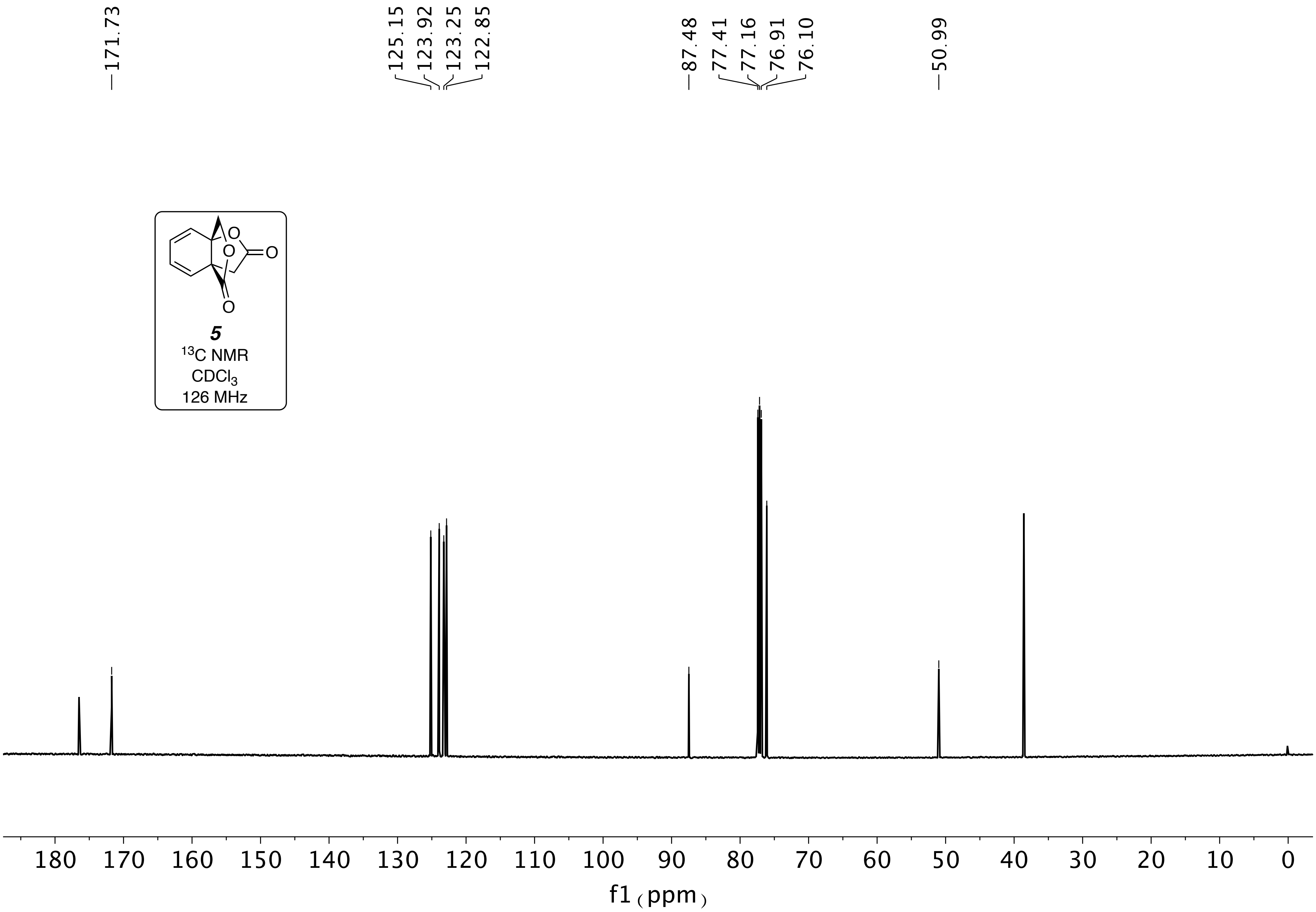
171.73
125.15
123.92
123.25
122.85
87.48
77.41
77.16
76.91
76.10
50.99
O
O
O
O
5
^{13}C NMR
CDCl$_3$
126 MHz
180
170
160
150
140
130
120
110
100
90
80
70
60
50
40
30
20
10
0
f1 (ppm)

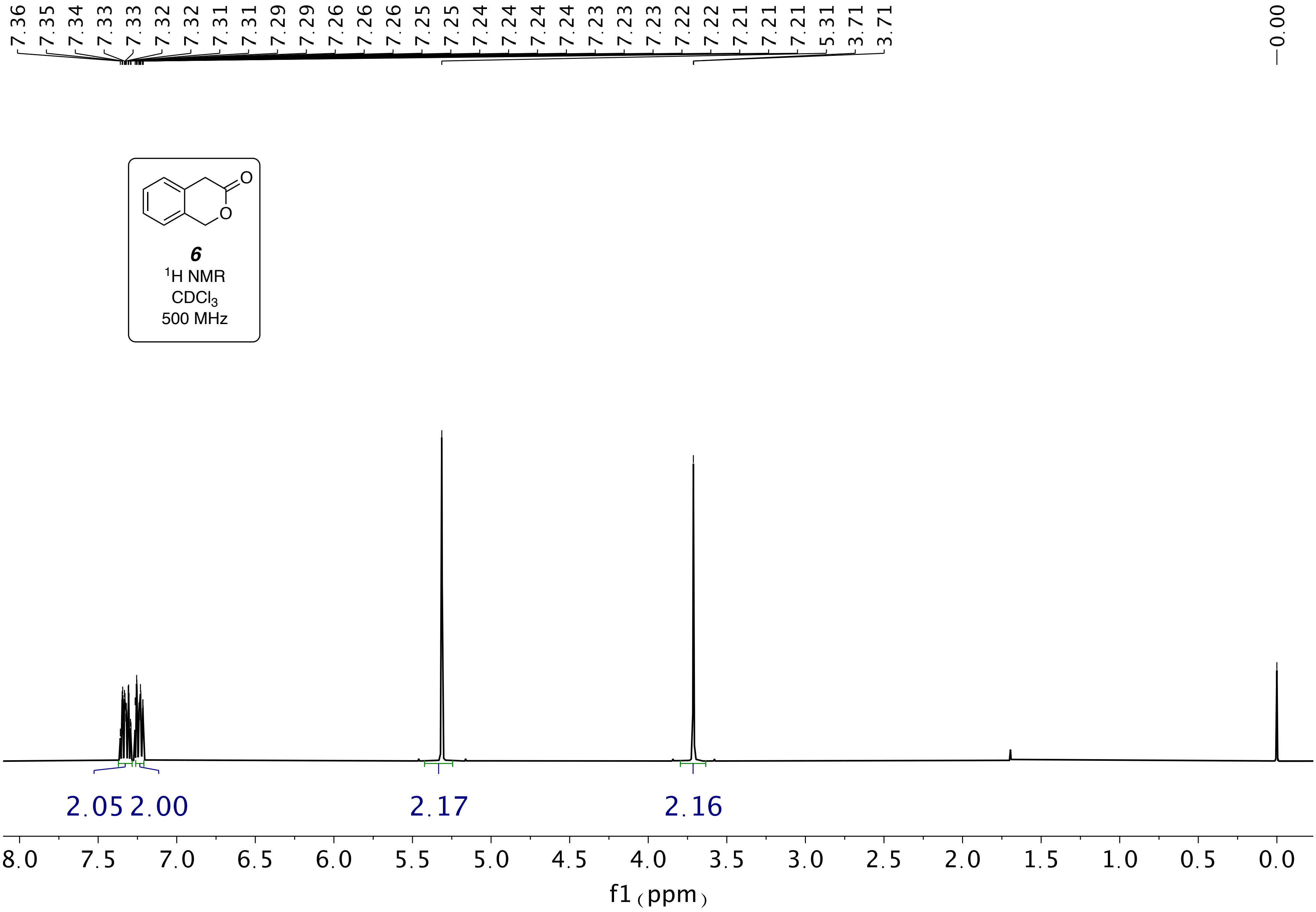
7.36 7.35 7.34 7.33 7.33 7.32 7.32 7.31 7.31 7.29 7.29 7.26 7.26 7.26 7.25 7.25 7.24 7.24 7.24 7.24 7.23 7.23 7.23 7.22 7.22 7.21 7.21 7.21 5.31 3.71 3.71
0.00
O
O
6
1H NMR
CDCl3
500 MHz
2.05 2.00
2.17
2.16
8.0 7.5 7.0 6.5 6.0 5.5 5.0 4.5 4.0 3.5 3.0 2.5 2.0 1.5 1.0 0.5 0.0
f1 (ppm)

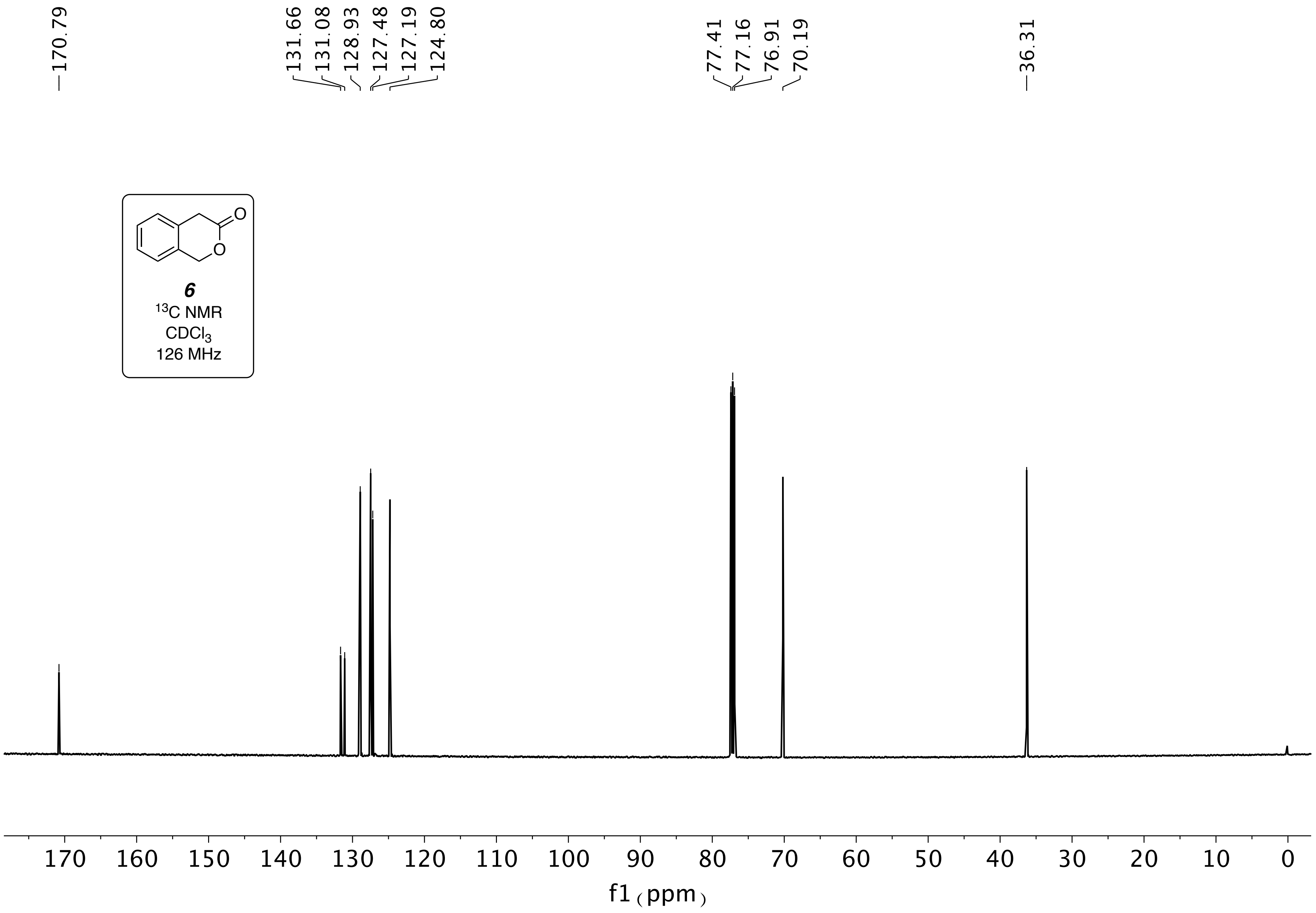
170.79
131.66
131.08
128.93
127.48
127.19
124.80
77.41
77.16
76.91
70.19
36.31
6
13C NMR
CDCl3
126 MHz
170
160
150
140
130
120
110
100
90
80
70
60
50
40
30
20
10
0
f1 (ppm)

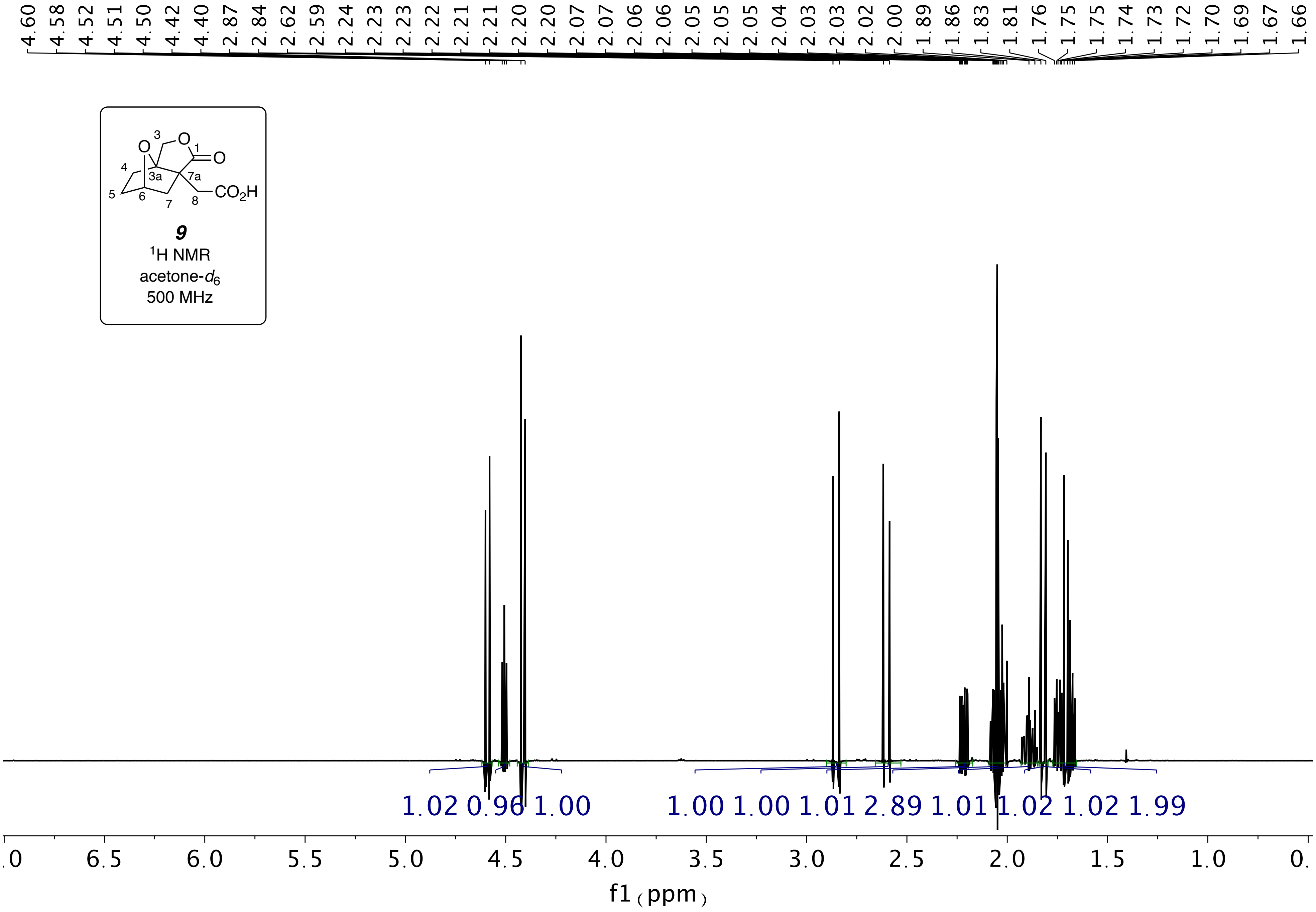
4.60 4.58 4.52 4.51 4.50 4.42 4.40 2.87 2.84 2.62 2.59 2.24 2.23 2.23 2.22 2.21 2.21 2.20 2.20 2.07 2.07 2.06 2.06 2.05 2.05 2.05 2.04 2.03 2.03 2.02 2.00 1.89 1.86 1.83 1.81 1.76 1.75 1.75 1.74 1.73 1.72 1.70 1.69 1.67 1.66
9
1H NMR
acetone-d6
500 MHz
1.02 0.96 1.00 1.00 1.00 1.01 2.89 1.01 1.02 1.02 1.99
f1 (ppm)

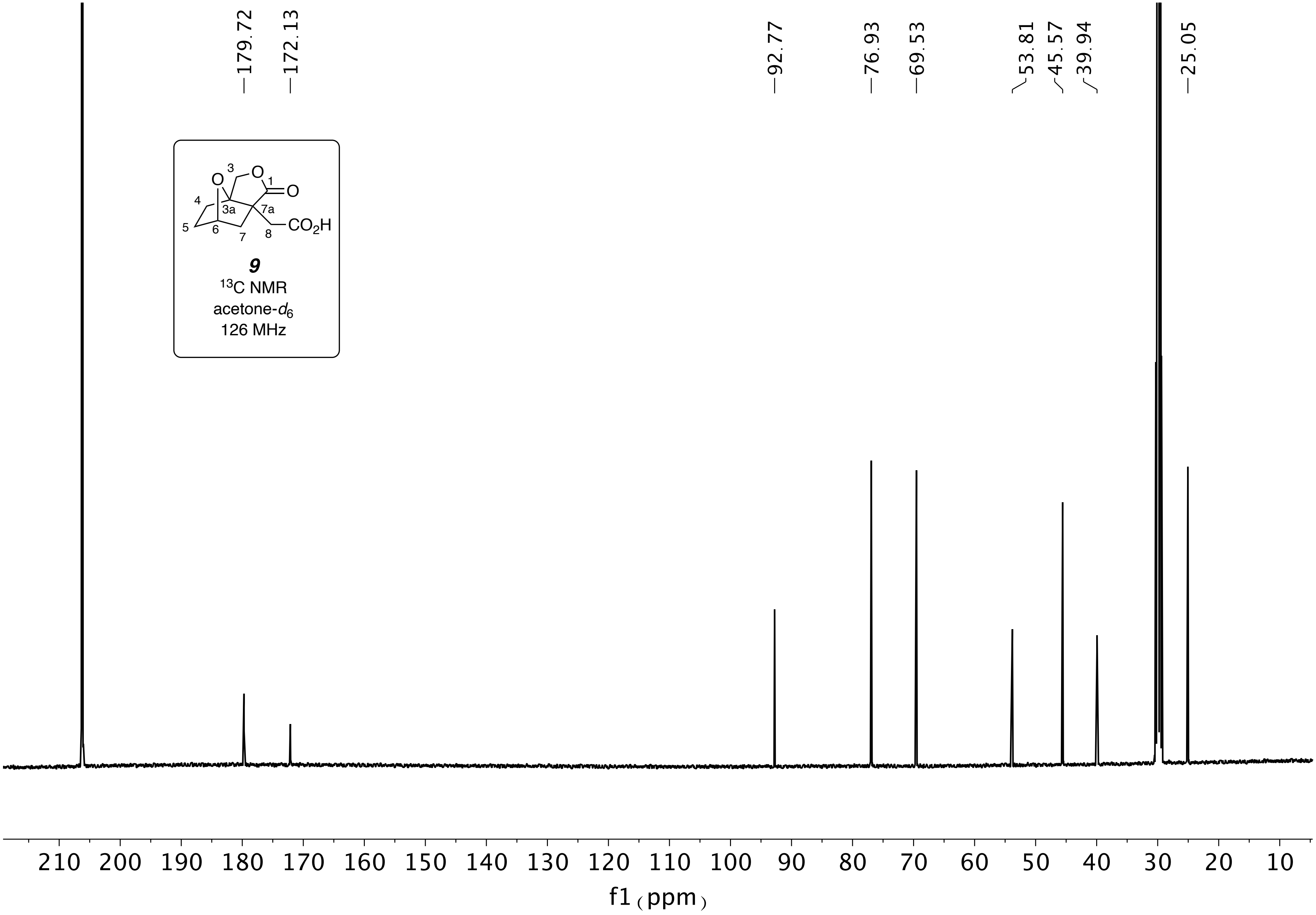
179.72
172.13
92.77
76.93
69.53
53.81
45.57
39.94
25.05
9
^{13}C NMR
acetone-d_6
126 MHz
210 200 190 180 170 160 150 140 130 120 110 100 90 80 70 60 50 40 30 20 10
f1 (ppm)

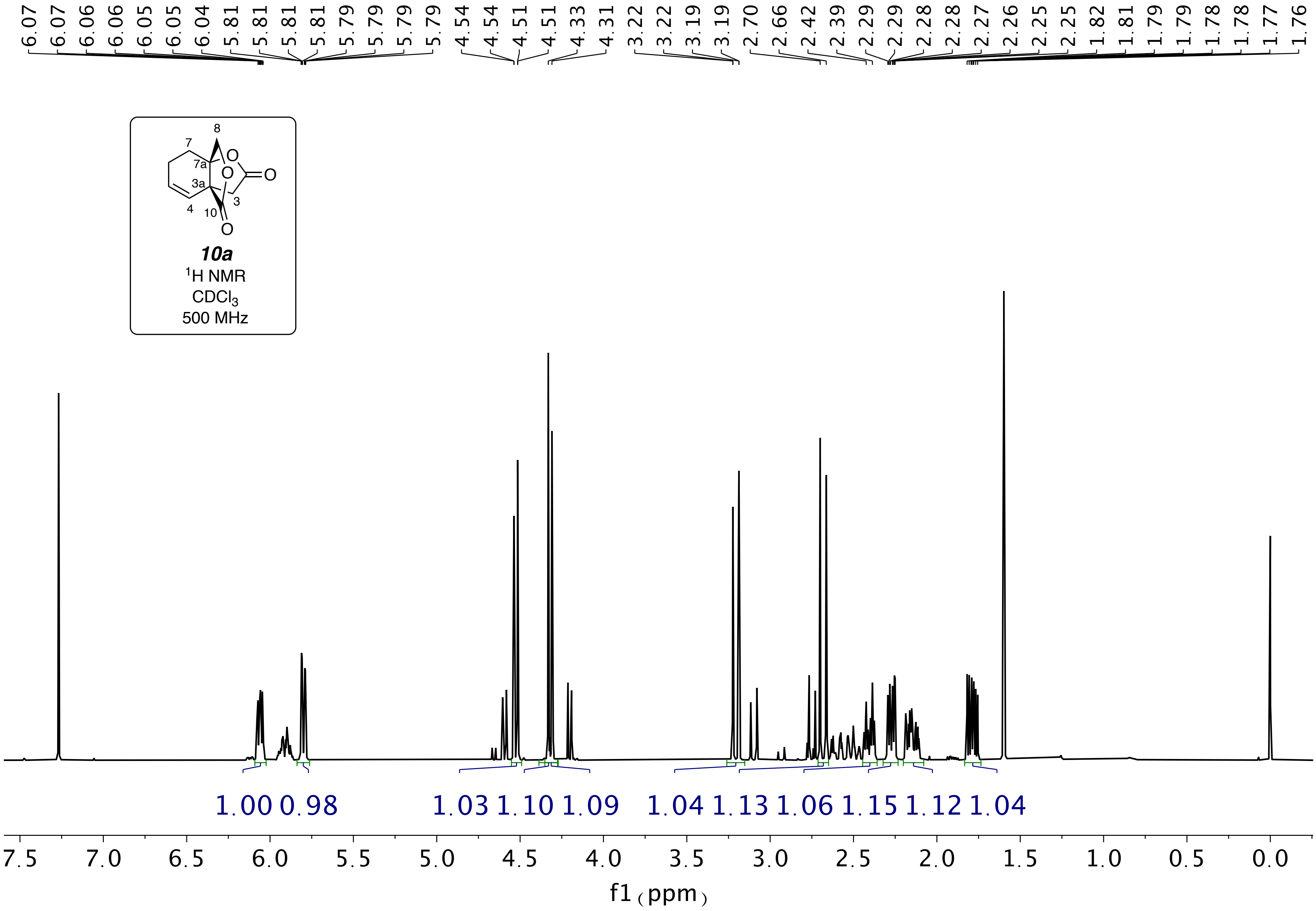
6.07
6.07
6.06
6.06
6.05
6.05
6.04
5.81
5.81
5.81
5.81
5.79
5.79
5.79
5.79
4.54
4.54
4.51
4.51
4.33
4.31
3.22
3.22
3.19
3.19
2.70
2.66
2.42
2.39
2.29
2.29
2.28
2.28
2.27
2.26
2.25
2.25
1.82
1.81
1.79
1.79
1.78
1.78
1.77
1.76
8
7
7a
3a
3
4
10
O
10a
^{1}H NMR
$CDCl_3$
500 MHz
1.00
0.98
1.03
1.10
1.09
1.04
1.13
1.06
1.15
1.12
1.04
7.5
7.0
6.5
6.0
5.5
5.0
4.5
4.0
3.5
3.0
2.5
2.0
1.5
1.0
0.5
0.0
f1 (ppm)

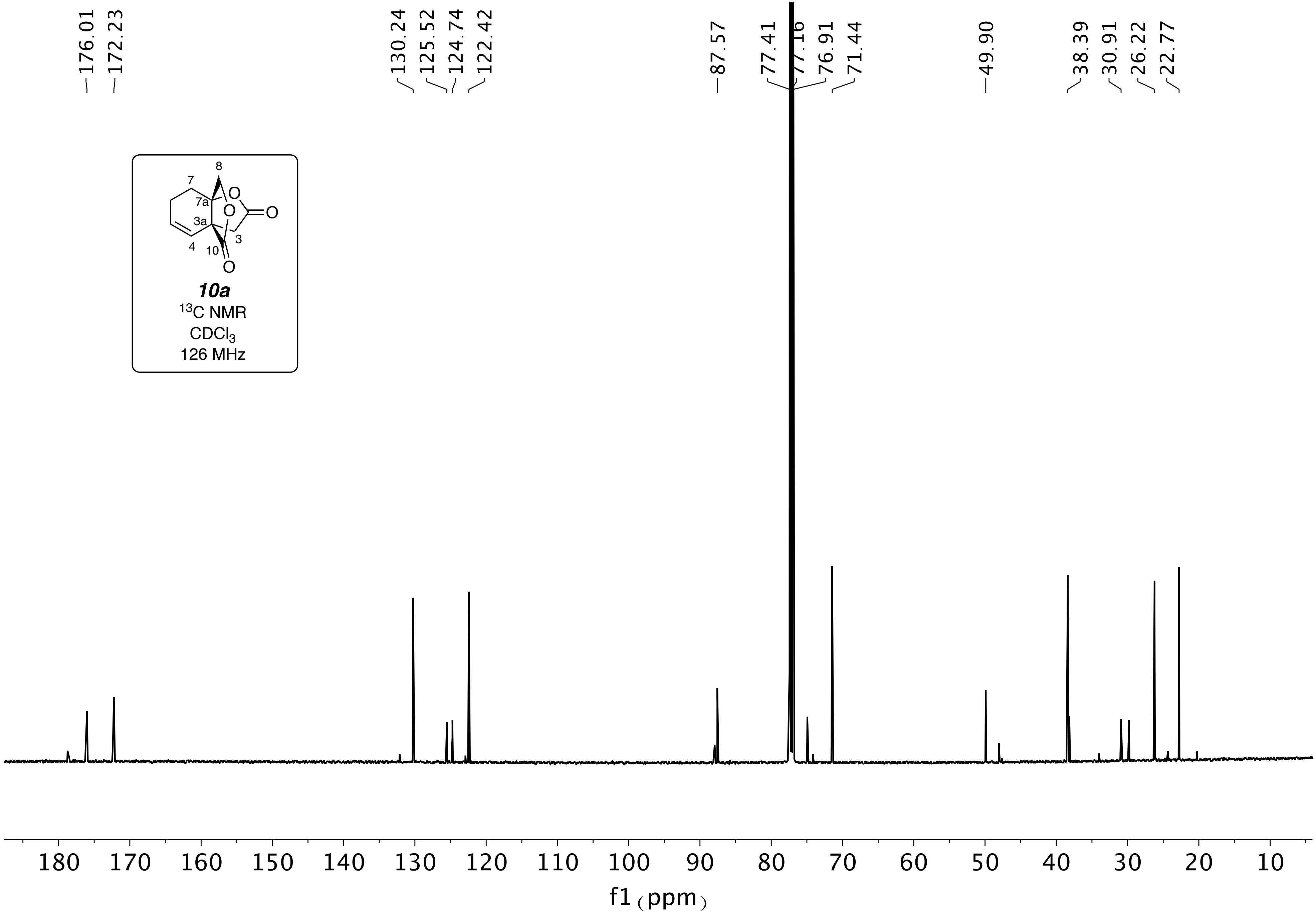
176.01
172.23
130.24
125.52
124.74
122.42
87.57
77.41
77.16
76.91
71.44
49.90
38.39
30.91
26.22
22.77
10a
13C NMR
CDCl3
126 MHz
180 170 160 150 140 130 120 110 100 90 80 70 60 50 40 30 20 10
f1 (ppm)

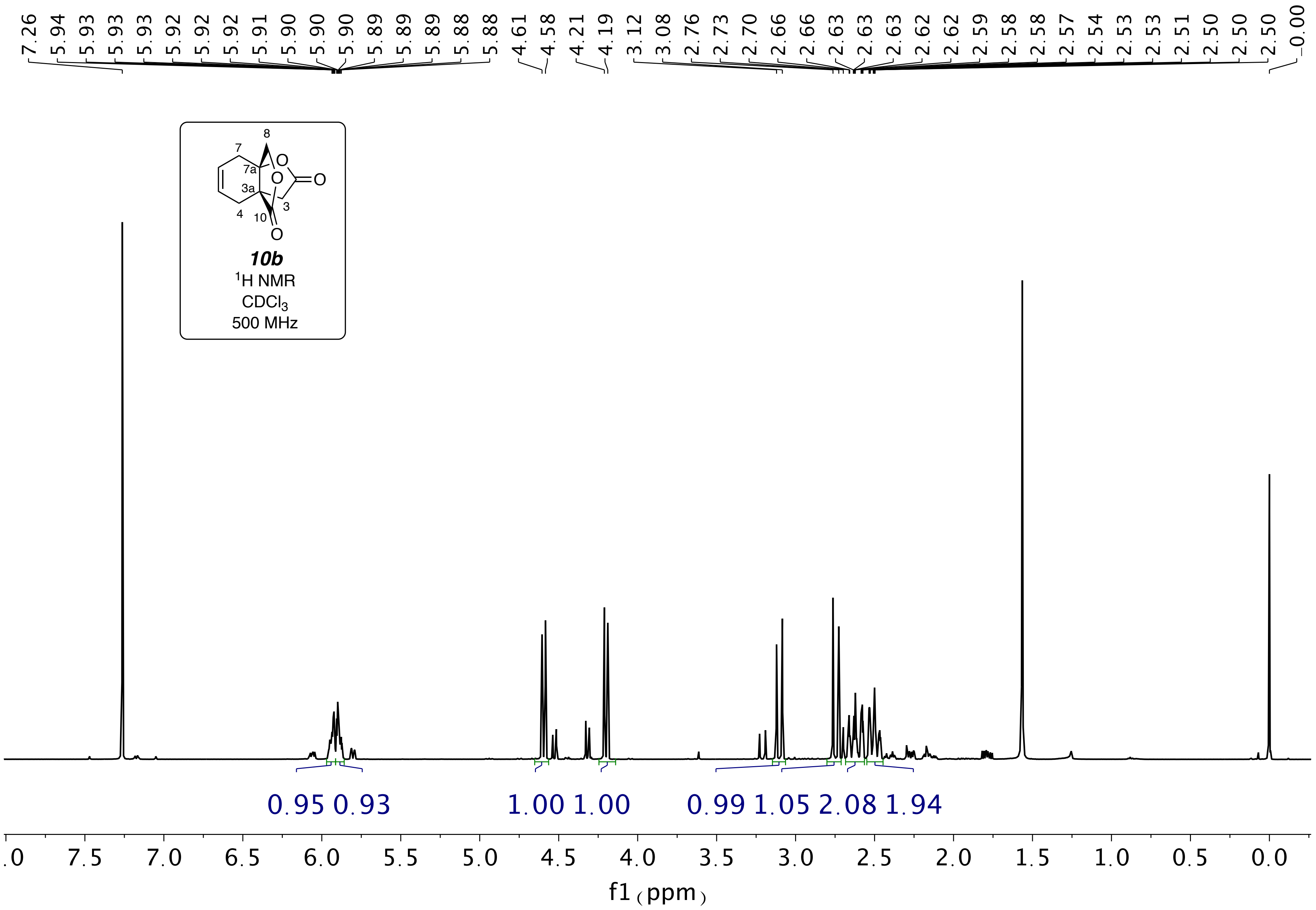
7.26
5.94
5.93
5.93
5.93
5.92
5.92
5.92
5.91
5.90
5.90
5.90
5.89
5.89
5.89
5.88
5.88
4.61
4.58
4.21
4.19
3.12
3.08
2.76
2.73
2.70
2.66
2.66
2.63
2.63
2.63
2.62
2.62
2.59
2.58
2.58
2.57
2.54
2.53
2.53
2.51
2.50
2.50
2.50
0.00
8
7
7a
3a
3
4
10
O
O
O
O
10b
^{1}H NMR
$CDCl_3$
500 MHz
0.95 0.93
1.00 1.00
0.99 1.05 2.08 1.94
f1 (ppm)

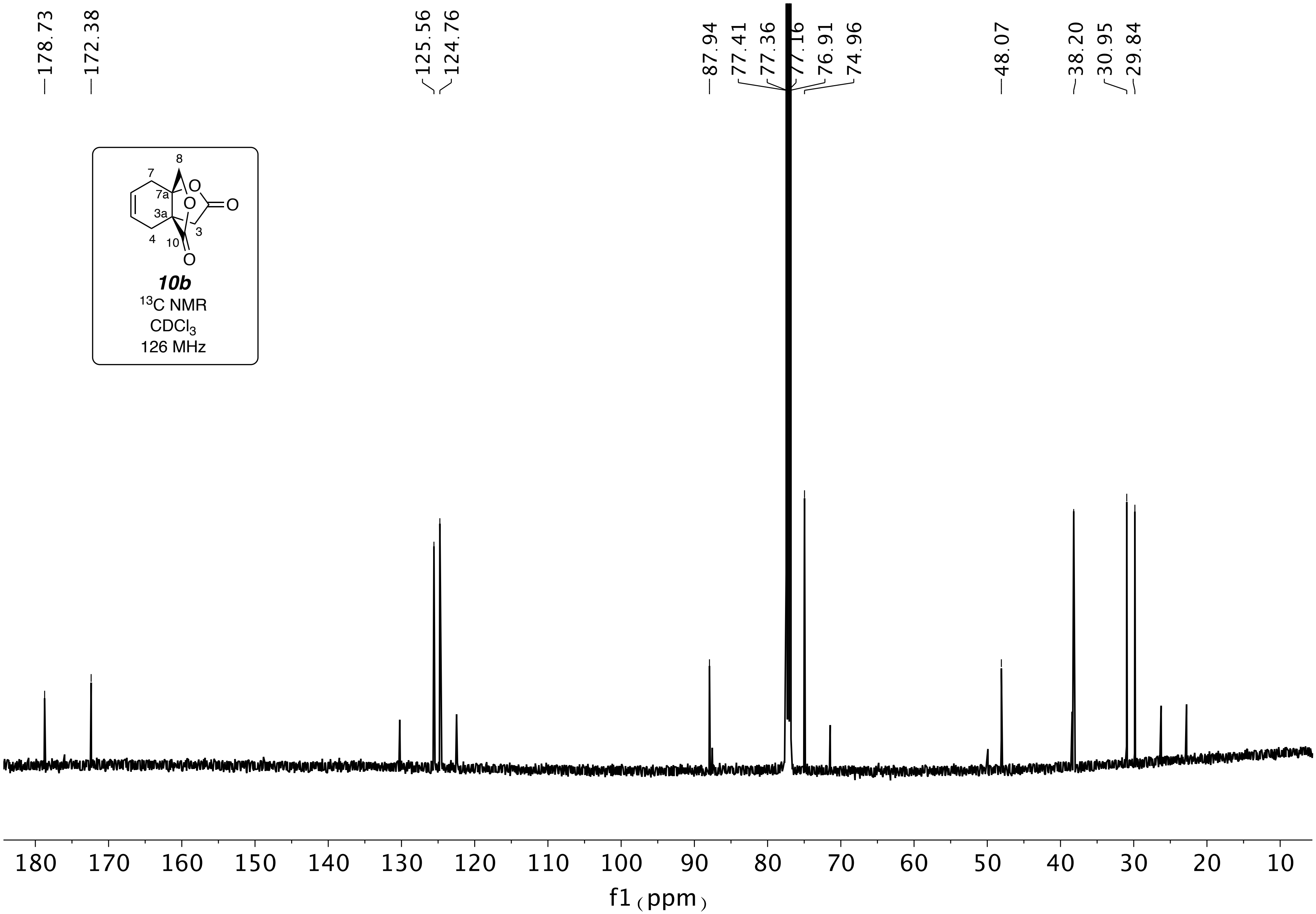
178.73
172.38
125.56
124.76
87.94
77.41
77.36
77.16
76.91
74.96
48.07
38.20
30.95
29.84
8
7
7a
3a
4
10
3
O
10b
^{13}C NMR
$CDCl_3$
126 MHz
180 170 160 150 140 130 120 110 100 90 80 70 60 50 40 30 20 10
f1 (ppm)

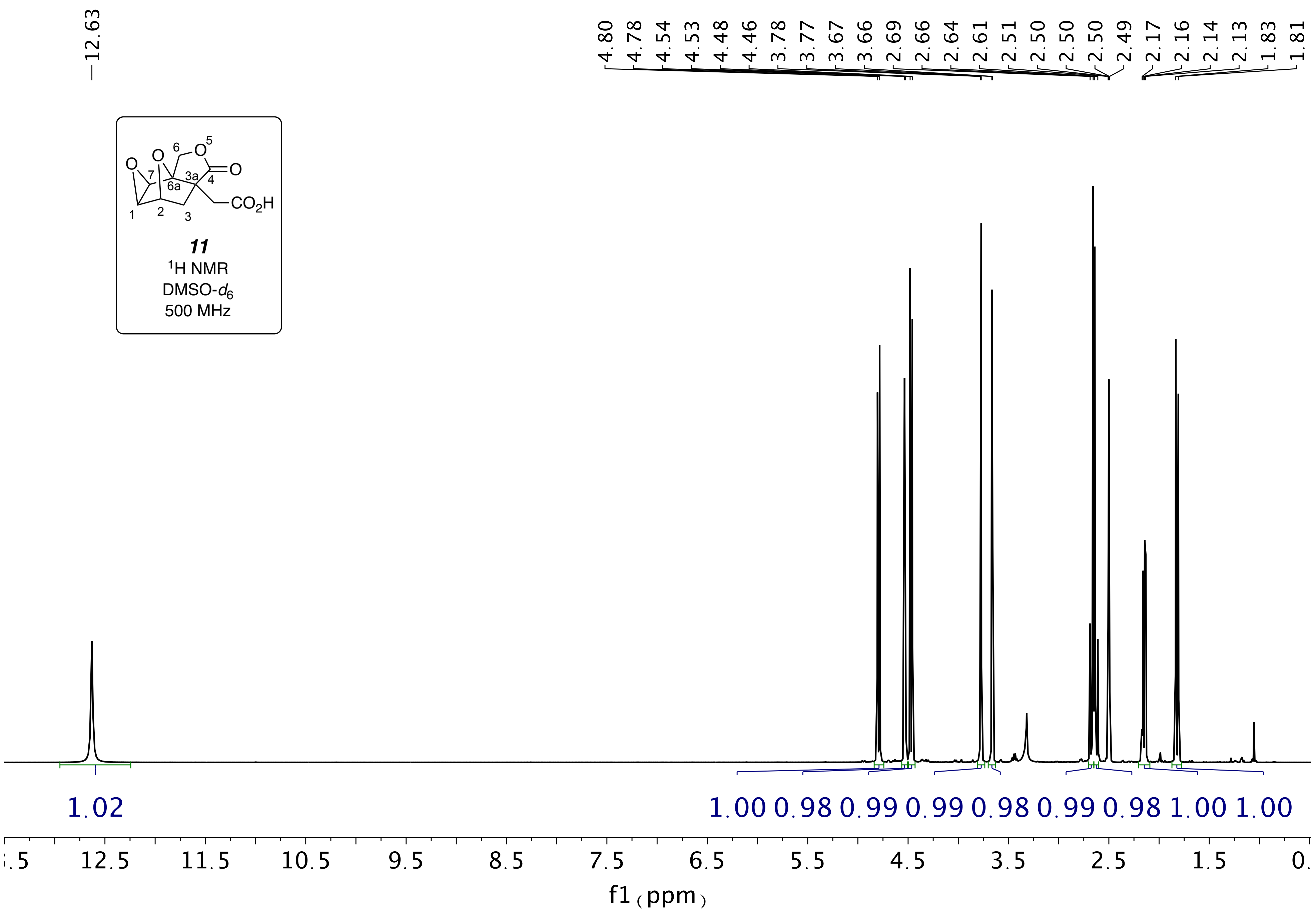

12.63
4.80
4.78
4.54
4.53
4.48
4.46
3.78
3.77
3.67
3.66
2.69
2.66
2.64
2.61
2.51
2.50
2.50
2.50
2.49
2.17
2.16
2.14
2.13
1.83
1.81
11
1H NMR
DMSO-d6
500 MHz
1.02
1.00 0.98 0.99 0.99 0.98 0.99 0.98 1.00 1.00
f1 (ppm)

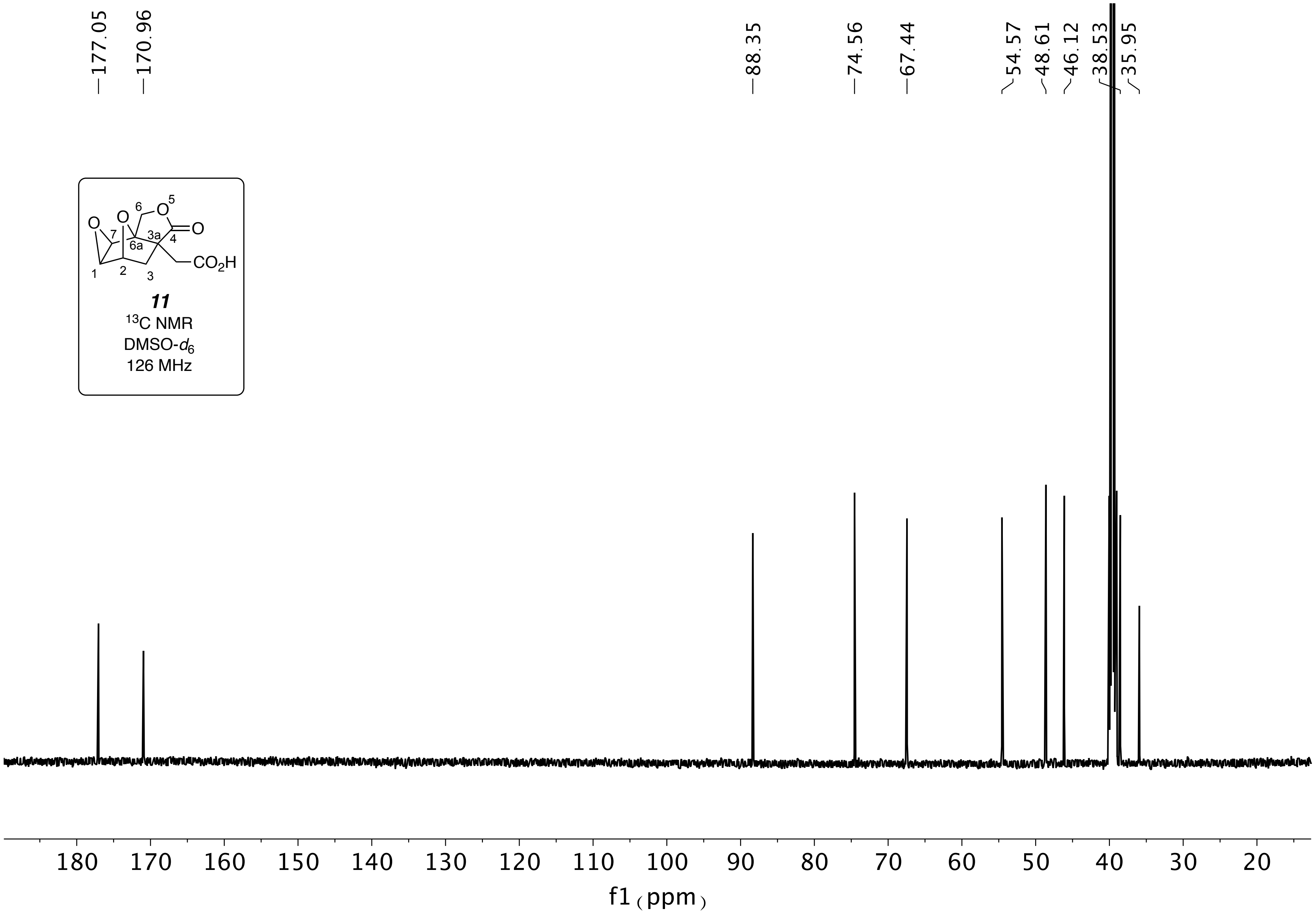
177.05
170.96
88.35
74.56
67.44
54.57
48.61
46.12
38.53
35.95
11
^{13}C NMR
DMSO-d_6
126 MHz
180 170 160 150 140 130 120 110 100 90 80 70 60 50 40 30 20
f1 (ppm)

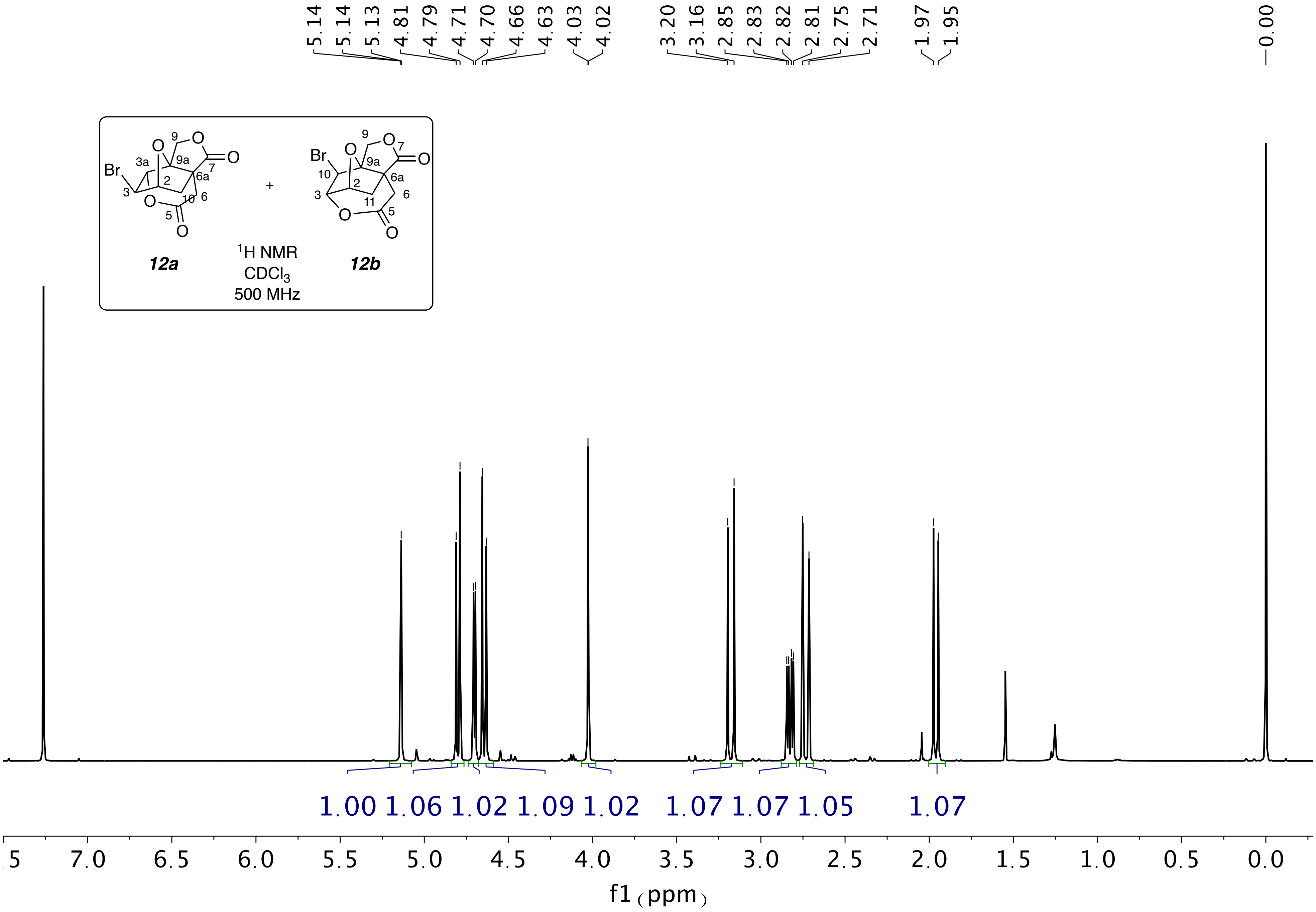
5.14
5.14
5.13
4.81
4.79
4.71
4.70
4.66
4.63
4.03
4.02
3.20
3.16
2.85
2.83
2.82
2.81
2.75
2.71
1.97
1.95
0.00
Br
O
9
O
3a
9a
7
O
2
6a
3
O
10
6
5
O
12a
+
Br
9
O
O
7
9a
O
10
2
6a
3
11
6
O
5
O
12b
^{1}H NMR
$CDCl_3$
500 MHz
1.00
1.06
1.02
1.09
1.02
1.07
1.07
1.05
1.07
.5
7.0
6.5
6.0
5.5
5.0
4.5
4.0
3.5
3.0
2.5
2.0
1.5
1.0
0.5
0.0
f1 (ppm)

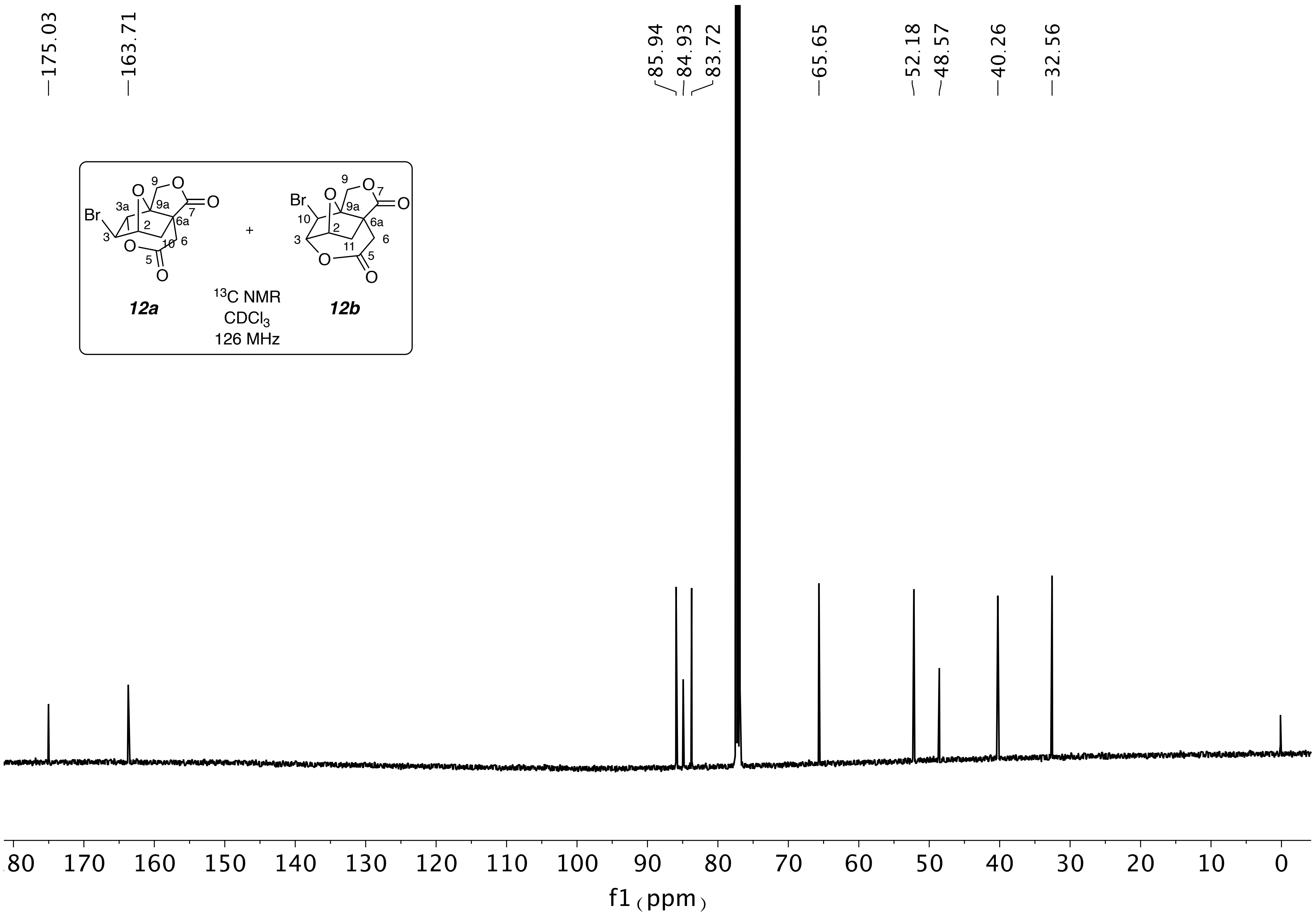
175.03
163.71
85.94
84.93
83.72
65.65
52.18
48.57
40.26
32.56
Br
O
O
O
O
O
9
3a
9a
7
2
6a
3
10
6
5
12a
13C NMR
CDCl3
126 MHz
12b
10
11
f1 (ppm)
180
170
160
150
140
130
120
110
100
90
80
70
60
50
40
30
20
10
0

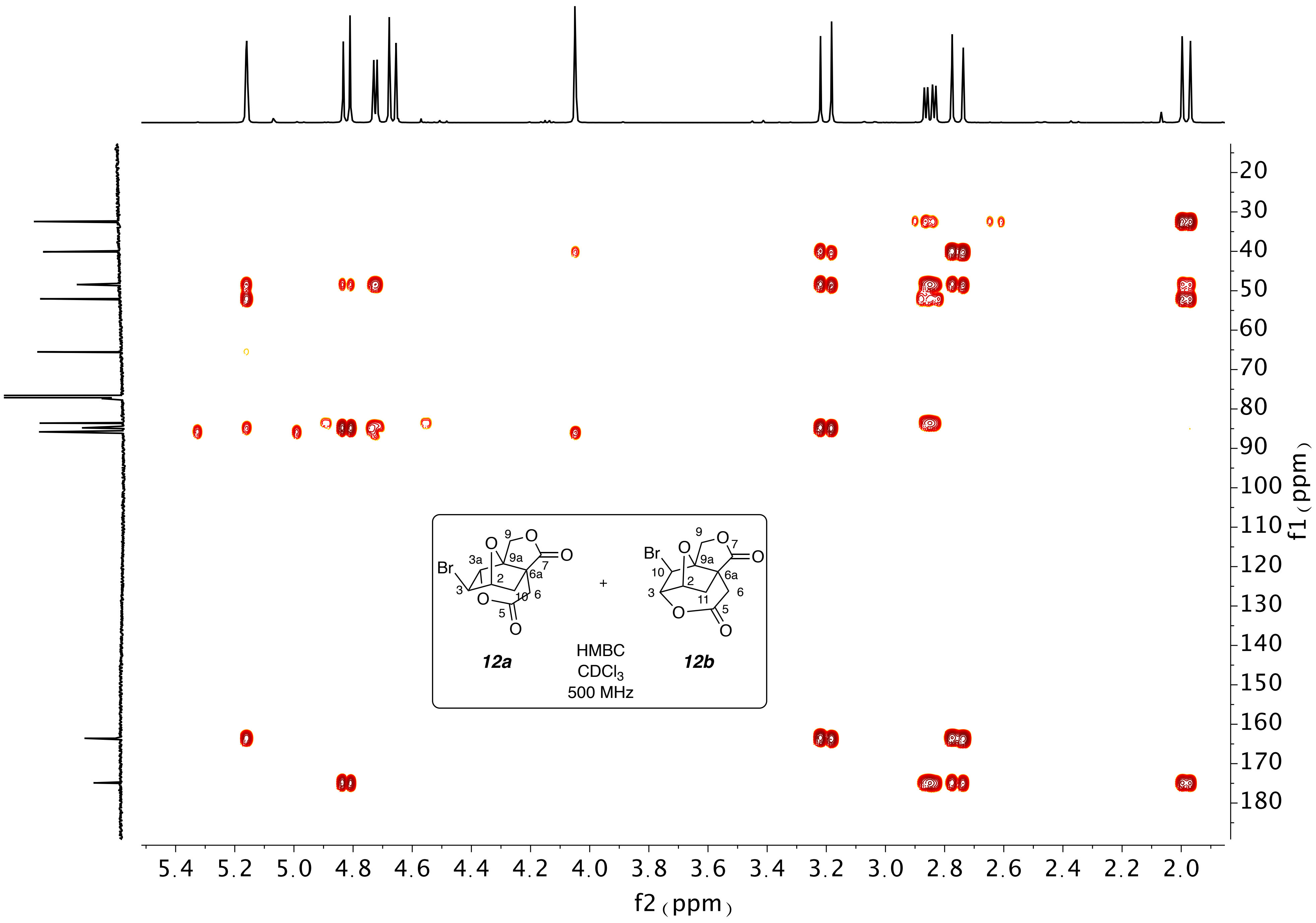
12a
12b
HMBC
$CDCl_3$
500 MHz
f2 (ppm)
f1 (ppm)

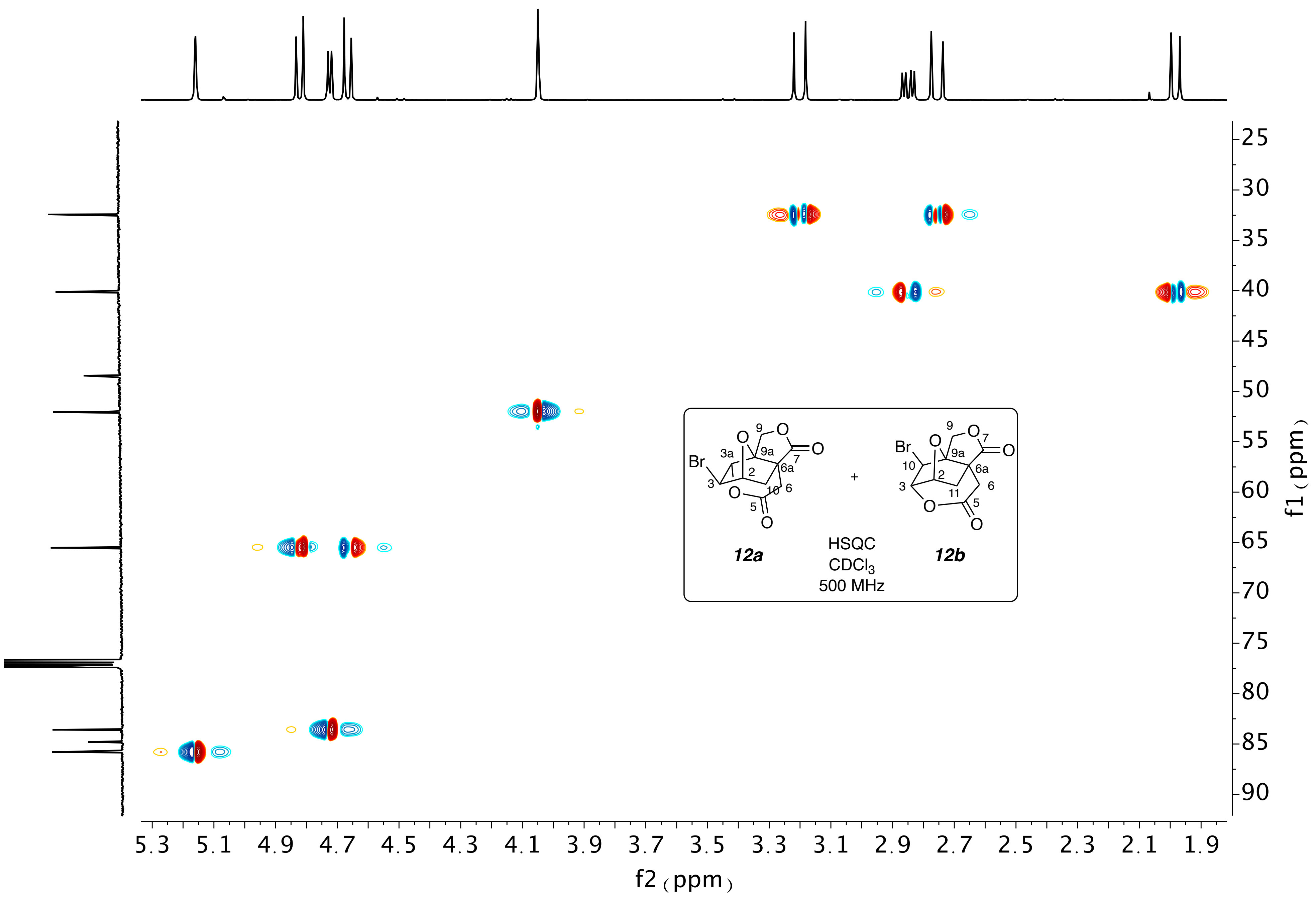
12a
12b
HSQC
$CDCl_3$
500 MHz
f2 (ppm)
f1 (ppm)

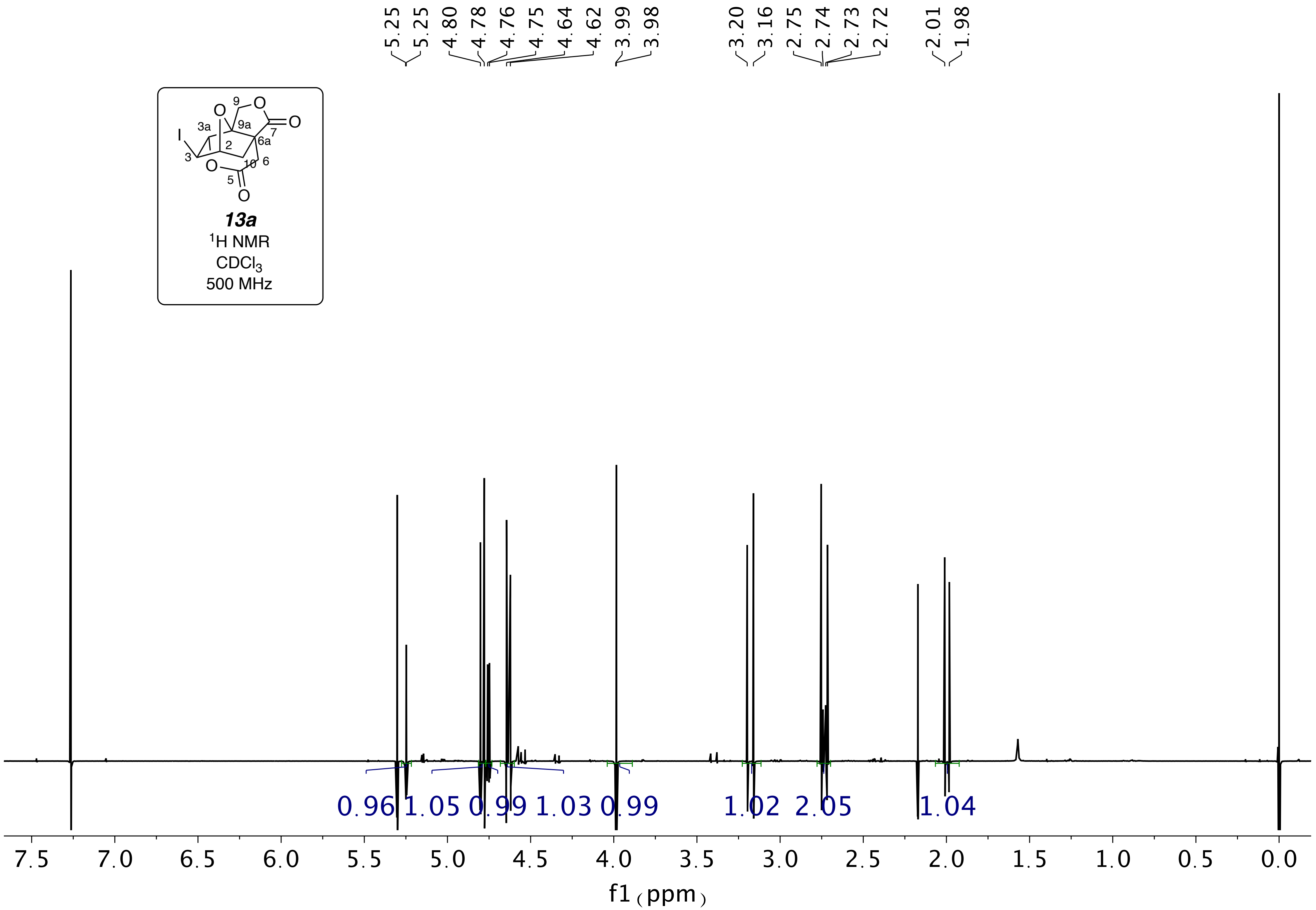
13a
^{1}H NMR
$CDCl_3$
500 MHz
5.25
5.25
4.80
4.78
4.76
4.75
4.64
4.62
3.99
3.98
3.20
3.16
2.75
2.74
2.73
2.72
2.01
1.98
0.96
1.05
0.99
1.03
0.99
1.02
2.05
1.04
f1 (ppm)

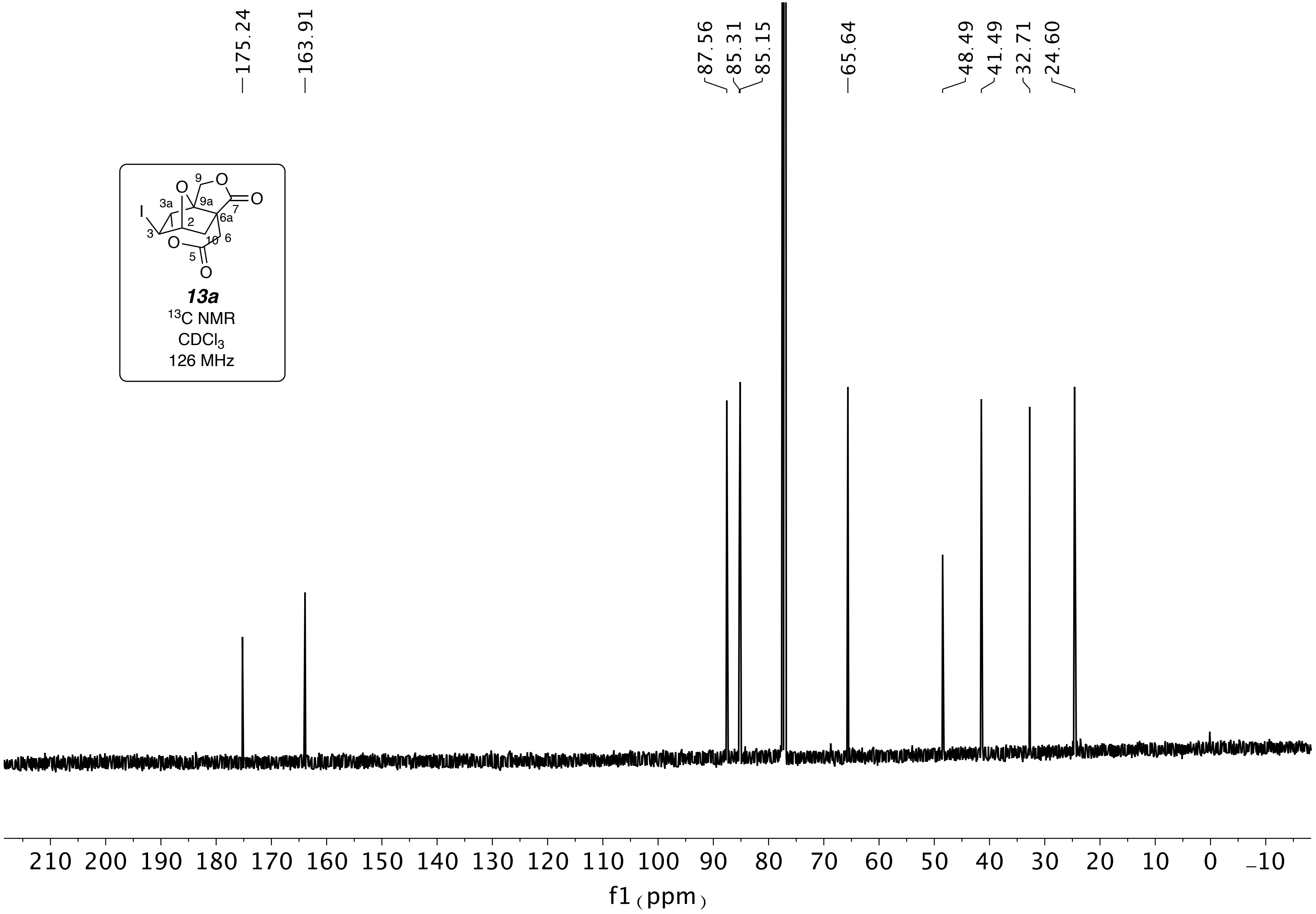
175.24
163.91
87.56
85.31
85.15
65.64
48.49
41.49
32.71
24.60
13a
^{13}C NMR
$CDCl_3$
126 MHz
f1 (ppm)

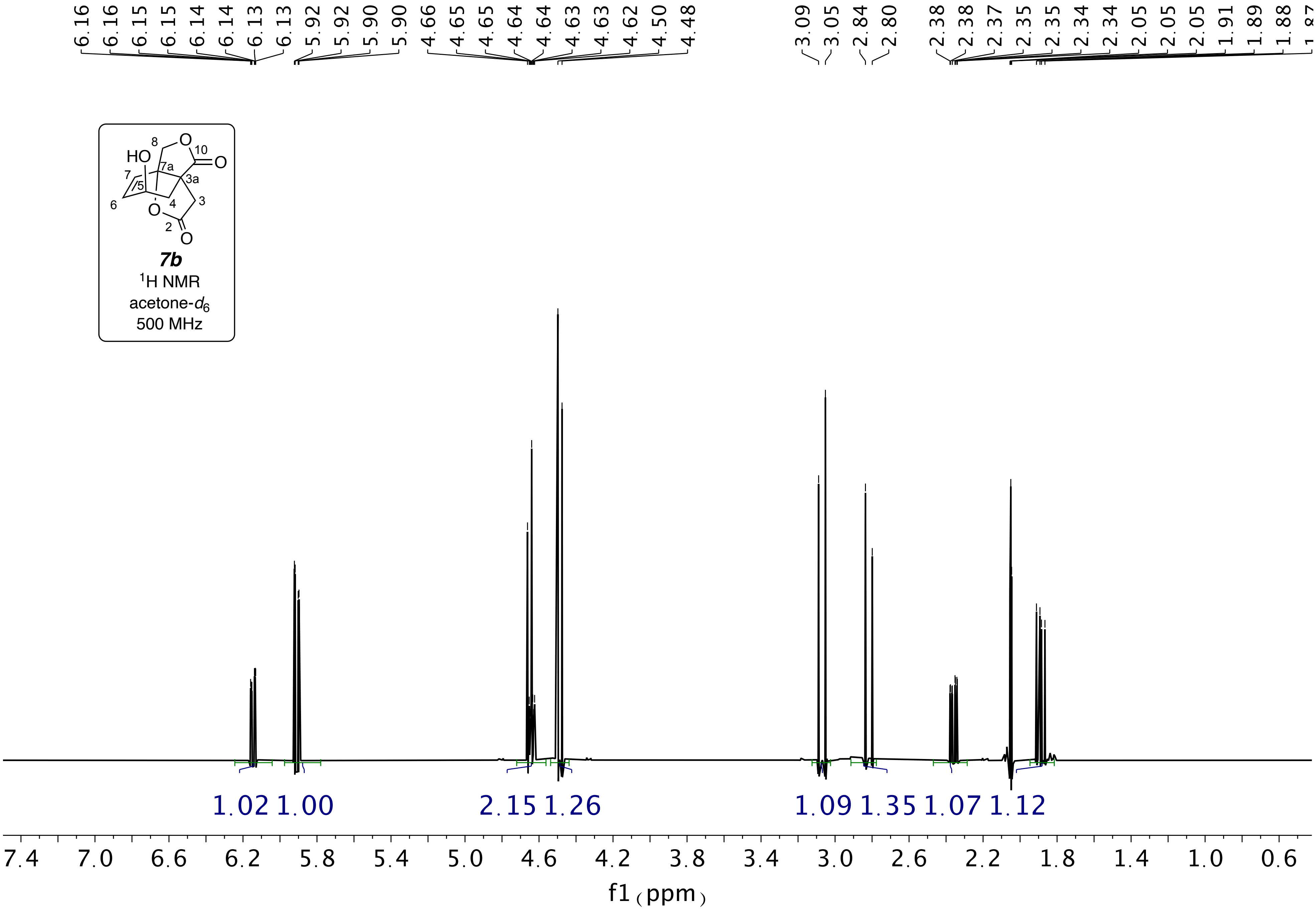
6.16 6.16 6.15 6.15 6.14 6.14 6.13 6.13 5.92 5.92 5.90 5.90 4.66 4.65 4.65 4.64 4.64 4.63 4.63 4.62 4.50 4.48
3.09 3.05 2.84 2.80
2.38 2.38 2.37 2.35 2.35 2.34 2.34 2.05 2.05 2.05 1.91 1.89 1.88
7b
^{1}H NMR
acetone-d_6
500 MHz
1.02 1.00 2.15 1.26 1.09 1.35 1.07 1.12
7.4 7.0 6.6 6.2 5.8 5.4 5.0 4.6 4.2 3.8 3.4 3.0 2.6 2.2 1.8 1.4 1.0 0.6
f1 (ppm)

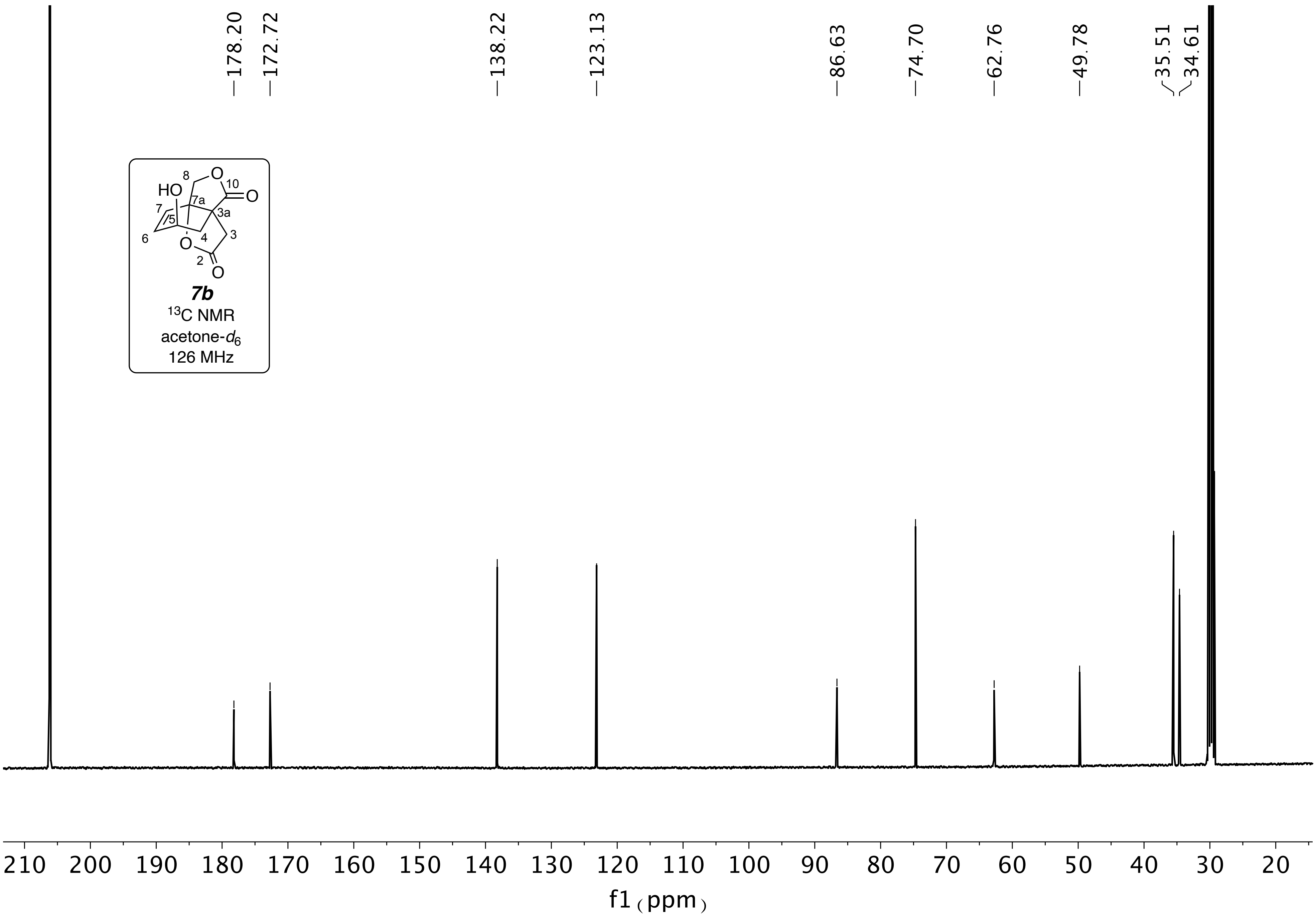

178.20
172.72
138.22
123.13
86.63
74.70
62.76
49.78
35.51
34.61
HO
O
O
O
O
O
8
10
7
7a
3a
5
6
4
3
2
7b
^{13}C NMR
acetone-d_6
126 MHz
210 200 190 180 170 160 150 140 130 120 110 100 90 80 70 60 50 40 30 20
f1 (ppm)

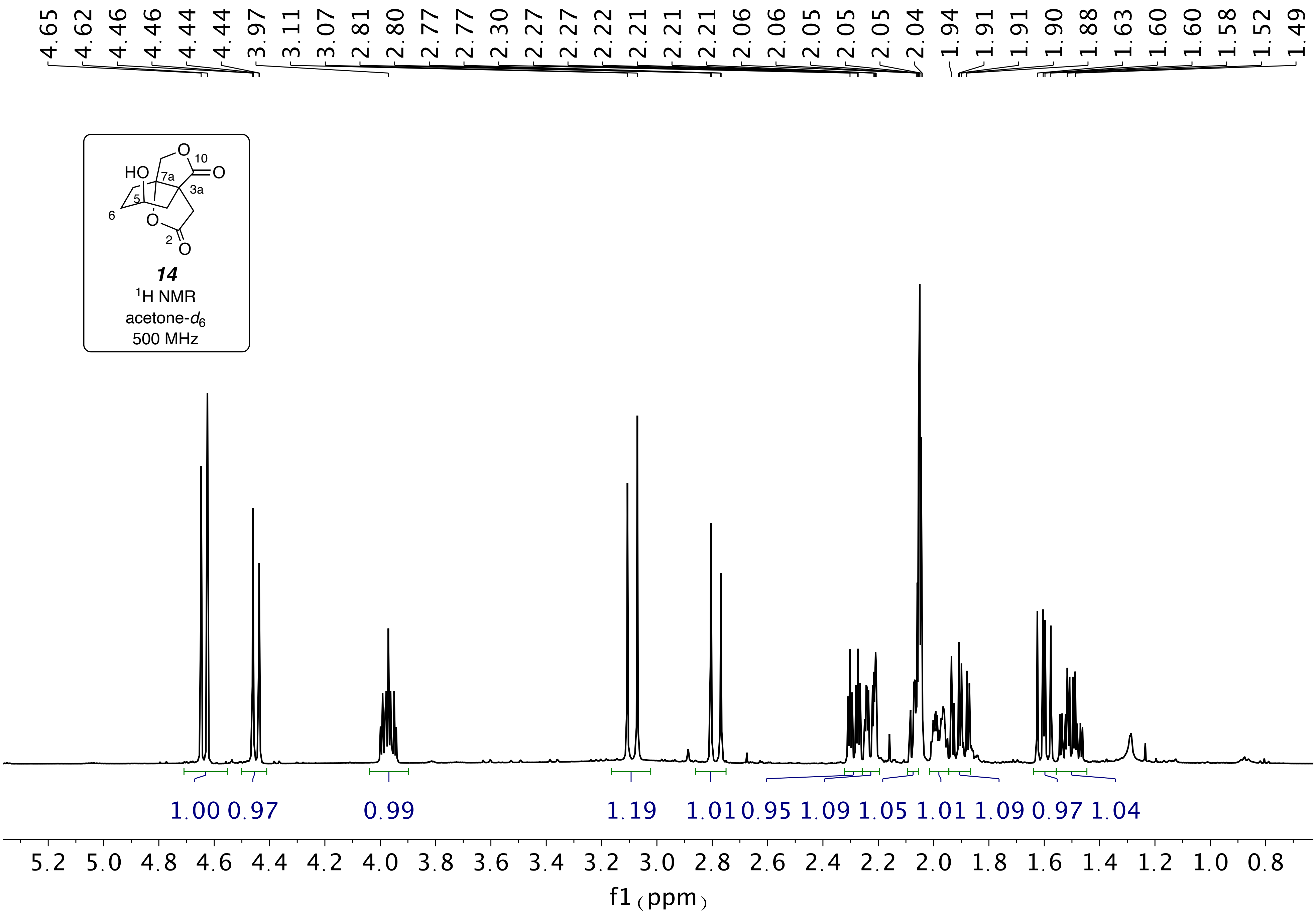

4.65 4.62 4.46 4.46 4.44 4.44 3.97 3.11 3.07 2.81 2.80 2.77 2.77 2.30 2.27 2.27 2.22 2.21 2.21 2.21 2.06 2.06 2.05 2.05 2.05 2.04 1.94 1.91 1.91 1.90 1.88 1.63 1.60 1.60 1.58 1.52 1.49
HO
7a
10
O
O
3a
5
6
O
2
O
14
¹H NMR
acetone-d6
500 MHz
1.00 0.97 0.99 1.19 1.01 0.95 1.09 1.05 1.01 1.09 0.97 1.04
5.2 5.0 4.8 4.6 4.4 4.2 4.0 3.8 3.6 3.4 3.2 3.0 2.8 2.6 2.4 2.2 2.0 1.8 1.6 1.4 1.2 1.0 0.8
f1 (ppm)

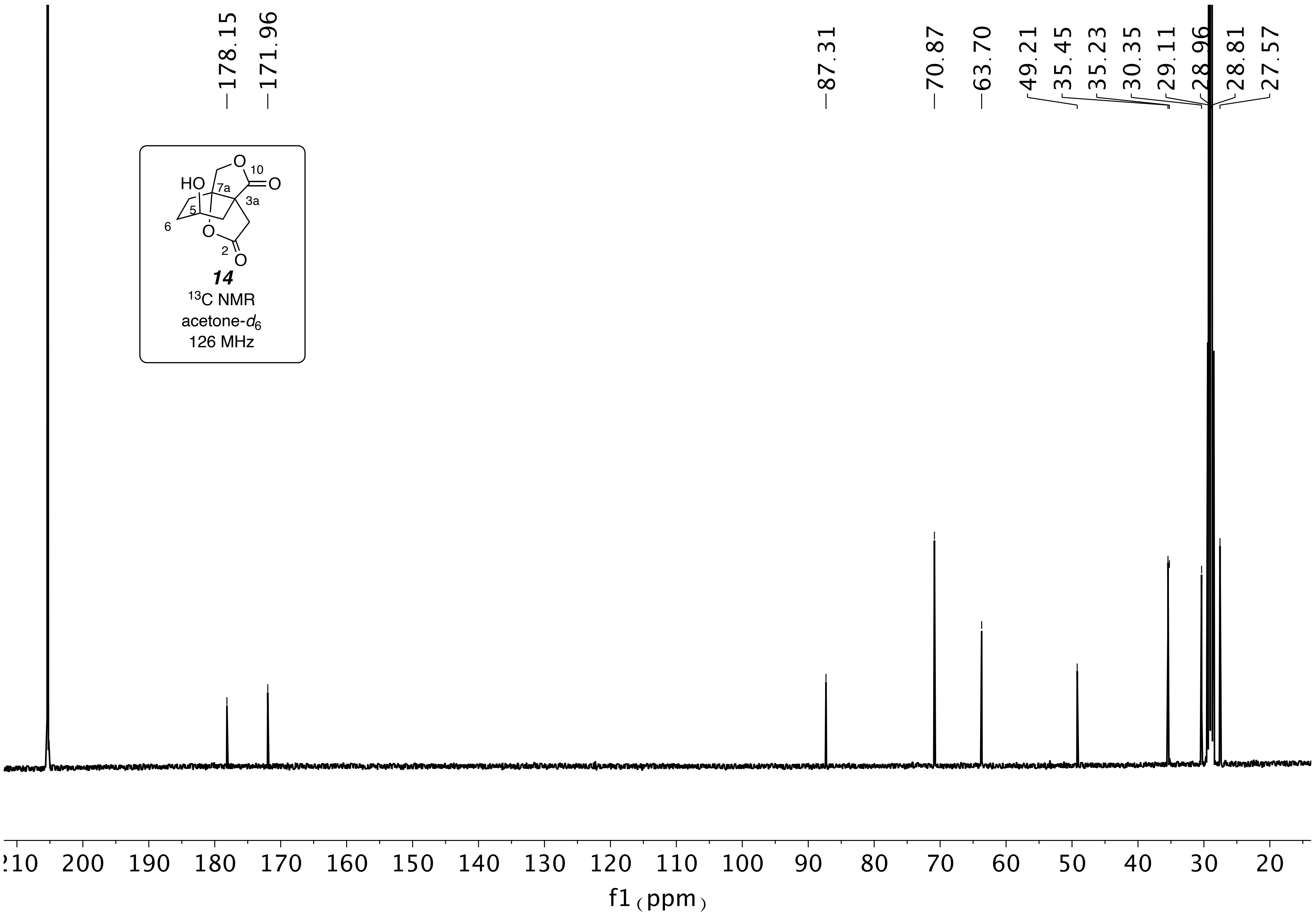
178.15
171.96
87.31
70.87
63.70
49.21
35.45
35.23
30.35
29.11
28.96
28.81
27.57
HO
O
10
O
7a
3a
5
6
O
2
O
14
13C NMR
acetone-d6
126 MHz
210
200
190
180
170
160
150
140
130
120
110
100
90
80
70
60
50
40
30
20
f1 (ppm)

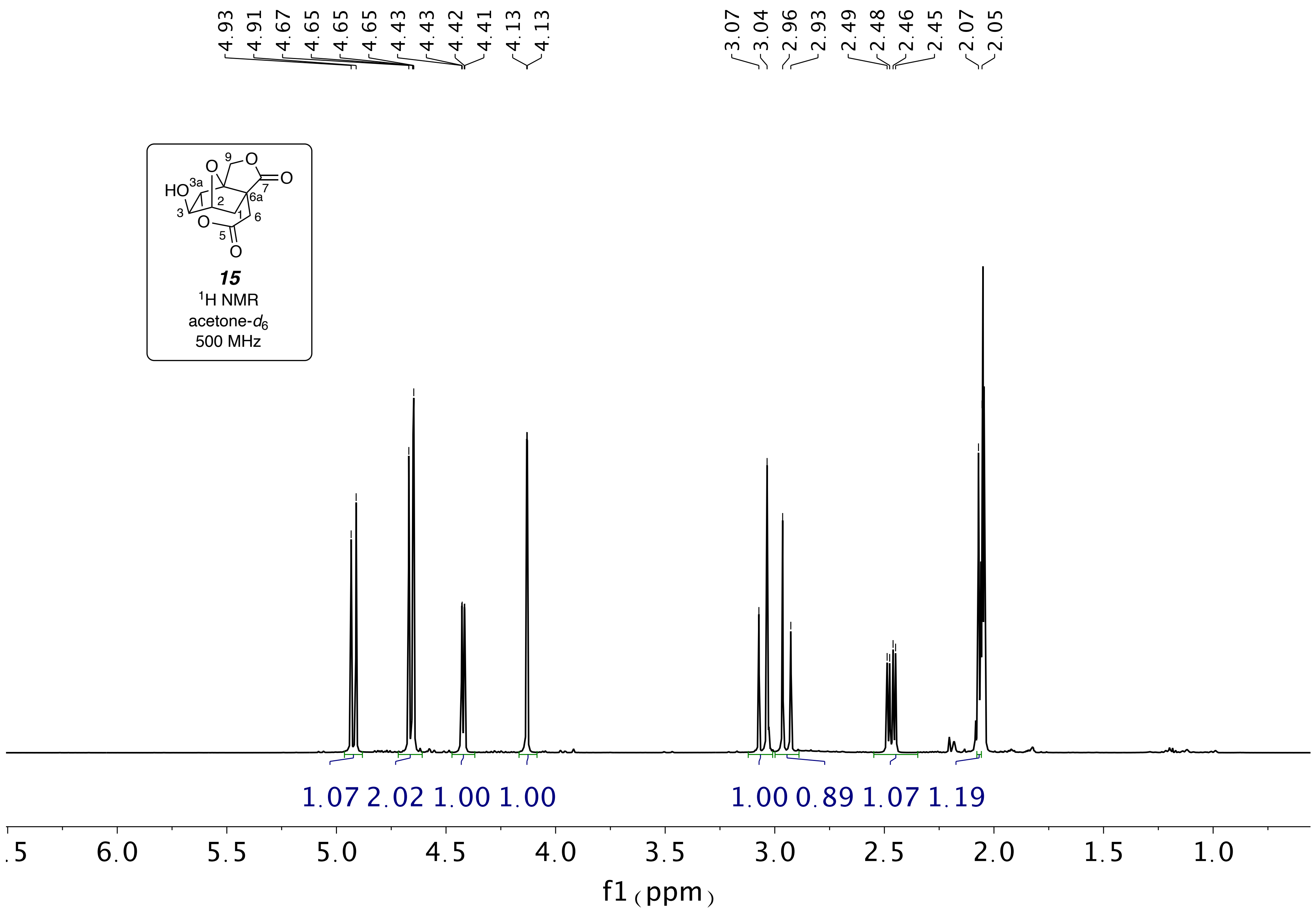
4.93
4.91
4.67
4.65
4.65
4.65
4.43
4.43
4.42
4.41
4.13
4.13
3.07
3.04
2.96
2.93
2.49
2.48
2.46
2.45
2.07
2.05
HO
3a
9
O
O
O
7
6a
2
1
6
3
O
5
O
15
1H NMR
acetone-d6
500 MHz
1.07
2.02
1.00
1.00
1.00
0.89
1.07
1.19
.5
6.0
5.5
5.0
4.5
4.0
3.5
3.0
2.5
2.0
1.5
1.0
f1 (ppm)

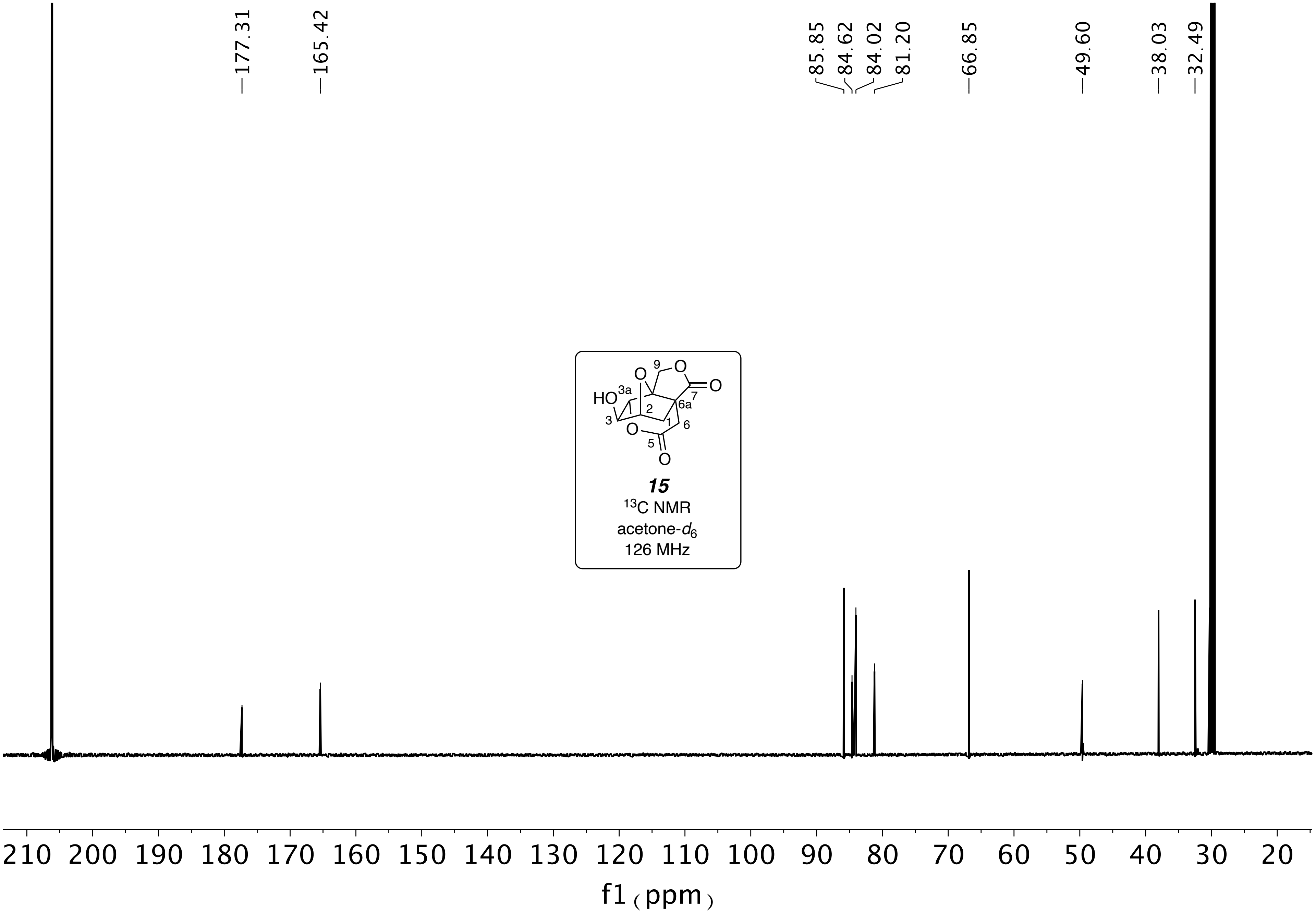
177.31
165.42
85.85
84.62
84.02
81.20
66.85
49.60
38.03
32.49
HO
O
O
O
O
O
3a
9
7
6a
2
1
3
6
5
15
13C NMR
acetone-d6
126 MHz
210 200 190 180 170 160 150 140 130 120 110 100 90 80 70 60 50 40 30 20
f1 (ppm)

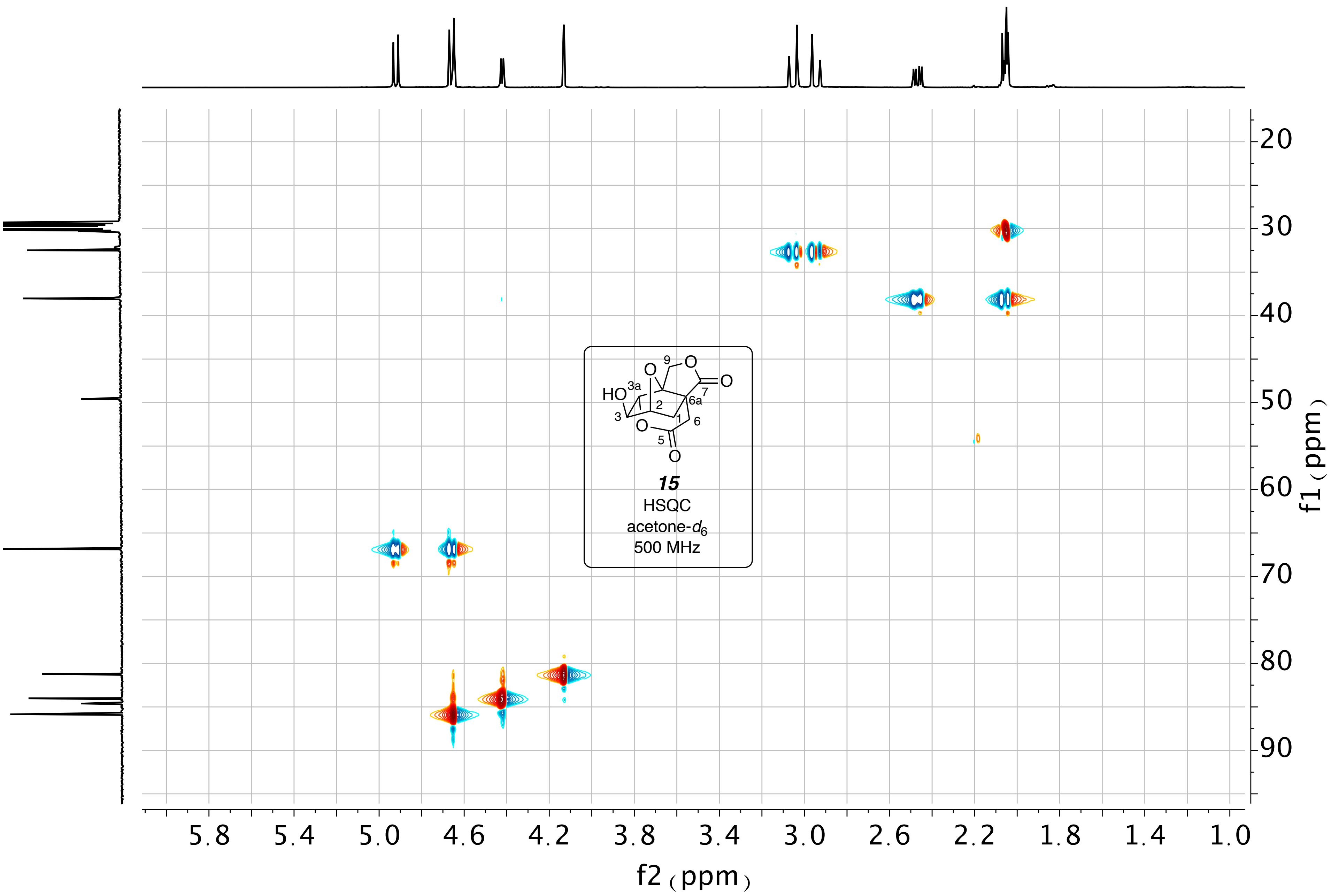

9
O
O
O
HO
3a
7
6a
2
3
1
6
O
5
O
15
HSQC
acetone-d_6
500 MHz
20
30
40
50
60
70
80
90
f1 (ppm)
5.8
5.4
5.0
4.6
4.2
3.8
3.4
3.0
2.6
2.2
1.8
1.4
1.0
f2 (ppm)

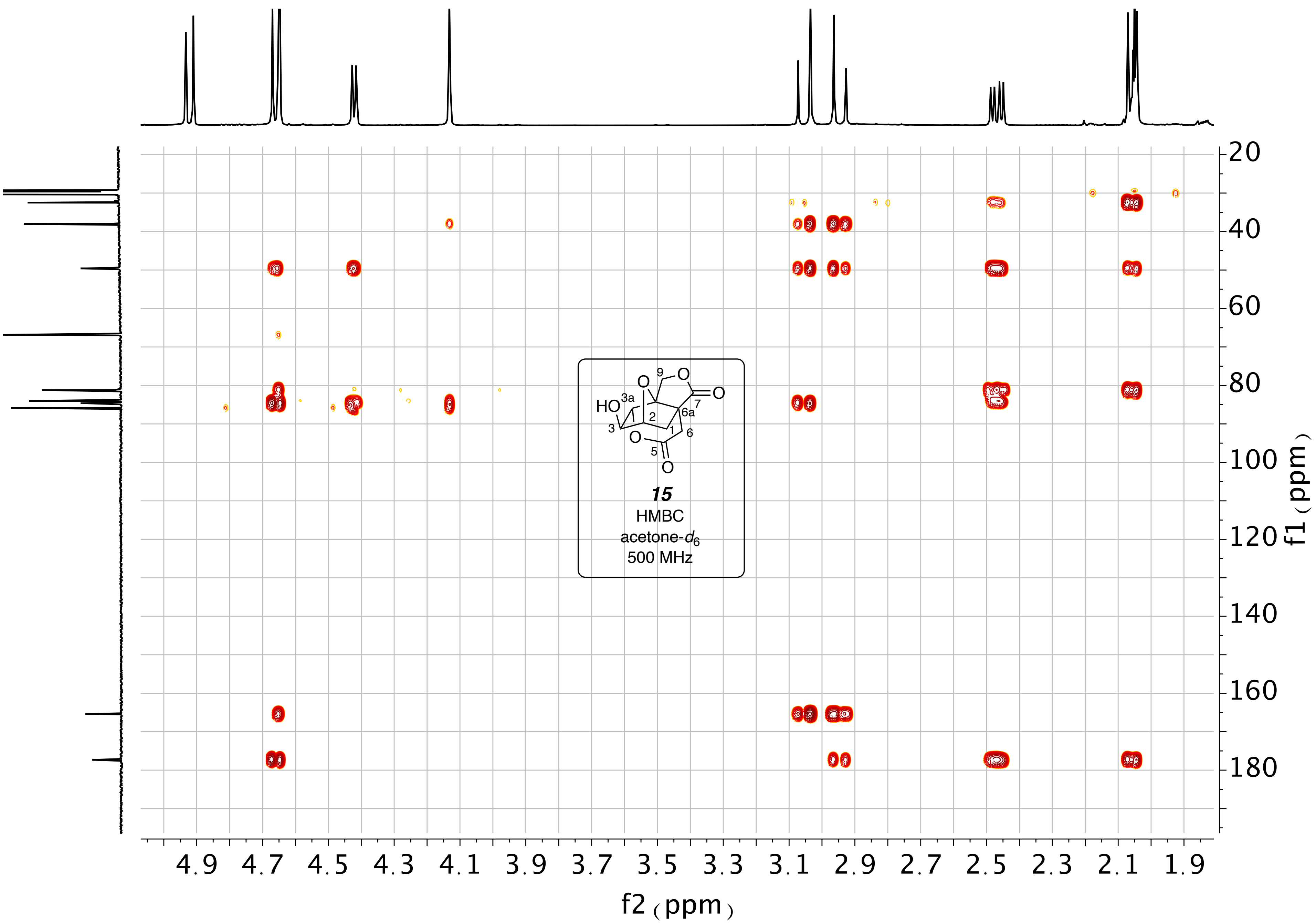
15
HMBC
acetone-d_6
500 MHz
f2 (ppm)
f1 (ppm)

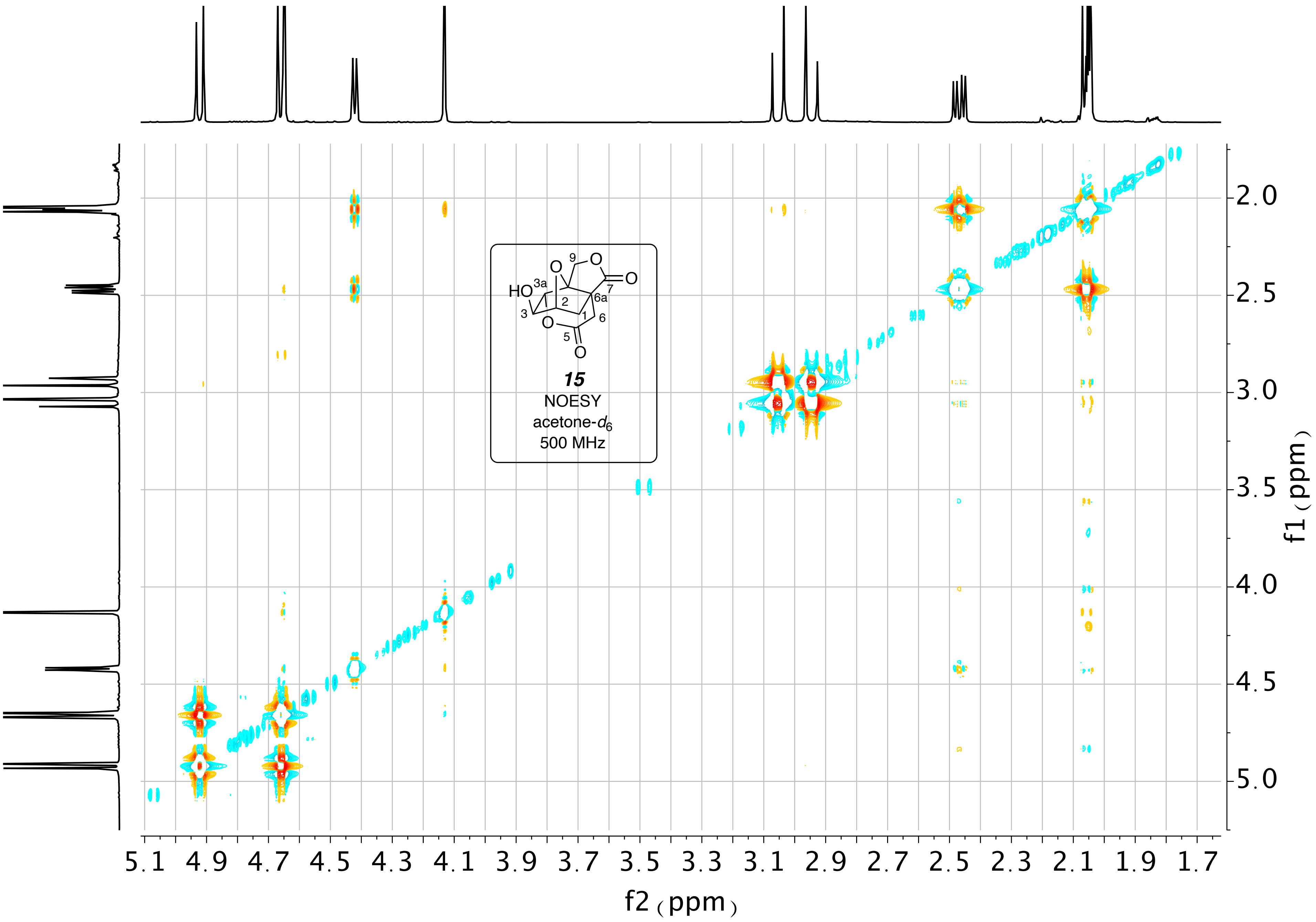

15
NOESY
acetone-d_6
500 MHz
f2 (ppm)
f1 (ppm)

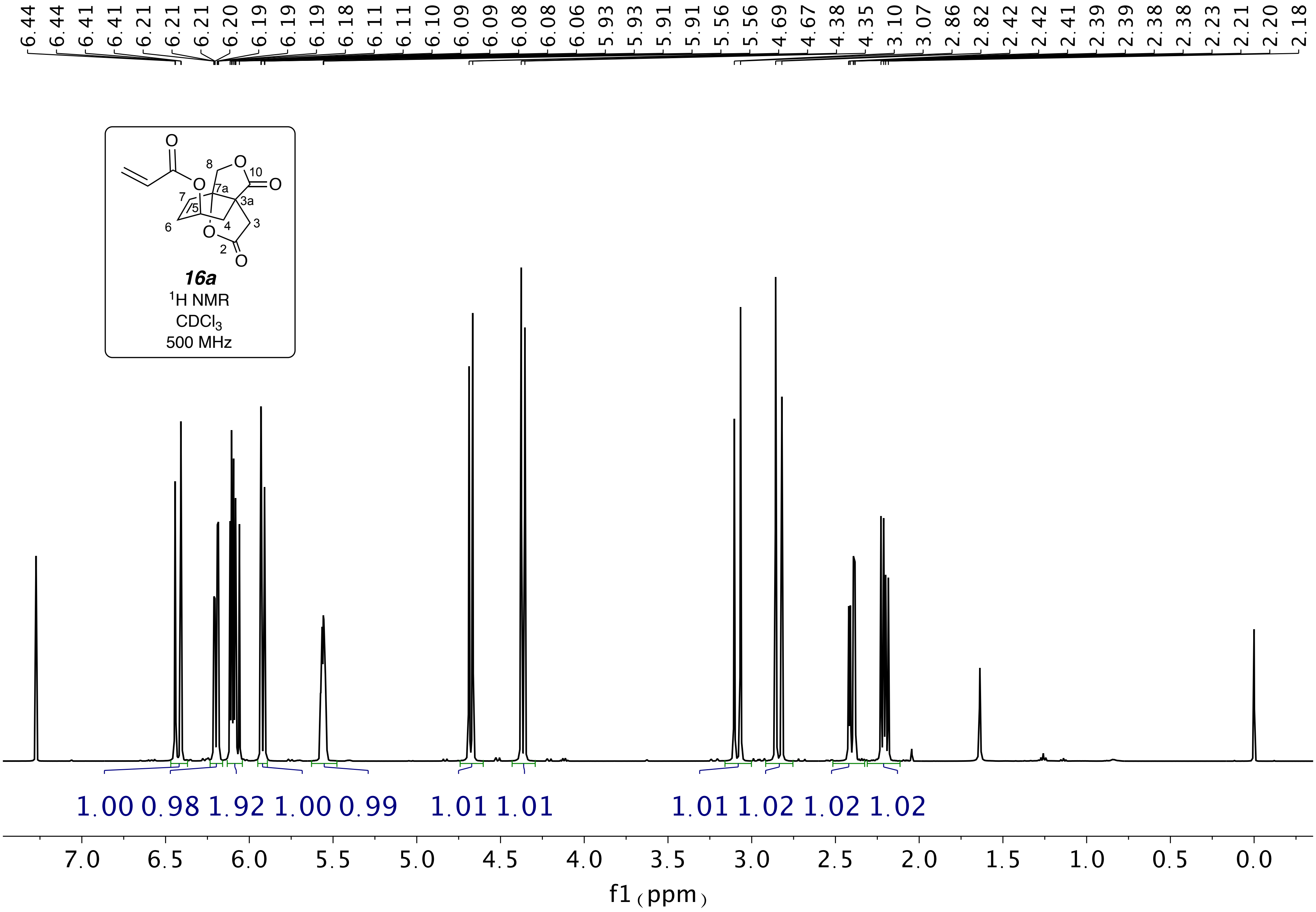
6.44
6.44
6.41
6.41
6.21
6.21
6.21
6.20
6.19
6.19
6.19
6.18
6.11
6.11
6.10
6.09
6.09
6.08
6.08
6.06
5.93
5.93
5.91
5.91
5.56
5.56
4.69
4.67
4.38
4.35
3.10
3.07
2.86
2.82
2.42
2.42
2.41
2.39
2.39
2.38
2.38
2.23
2.21
2.20
2.18
O
8
O
10
O
O
7a
7
3a
5
6
4
3
O
2
O
16a
1H NMR
CDCl3
500 MHz
1.00
0.98
1.92
1.00
0.99
1.01
1.01
1.01
1.02
1.02
1.02
7.0
6.5
6.0
5.5
5.0
4.5
4.0
3.5
3.0
2.5
2.0
1.5
1.0
0.5
0.0
f1 (ppm)

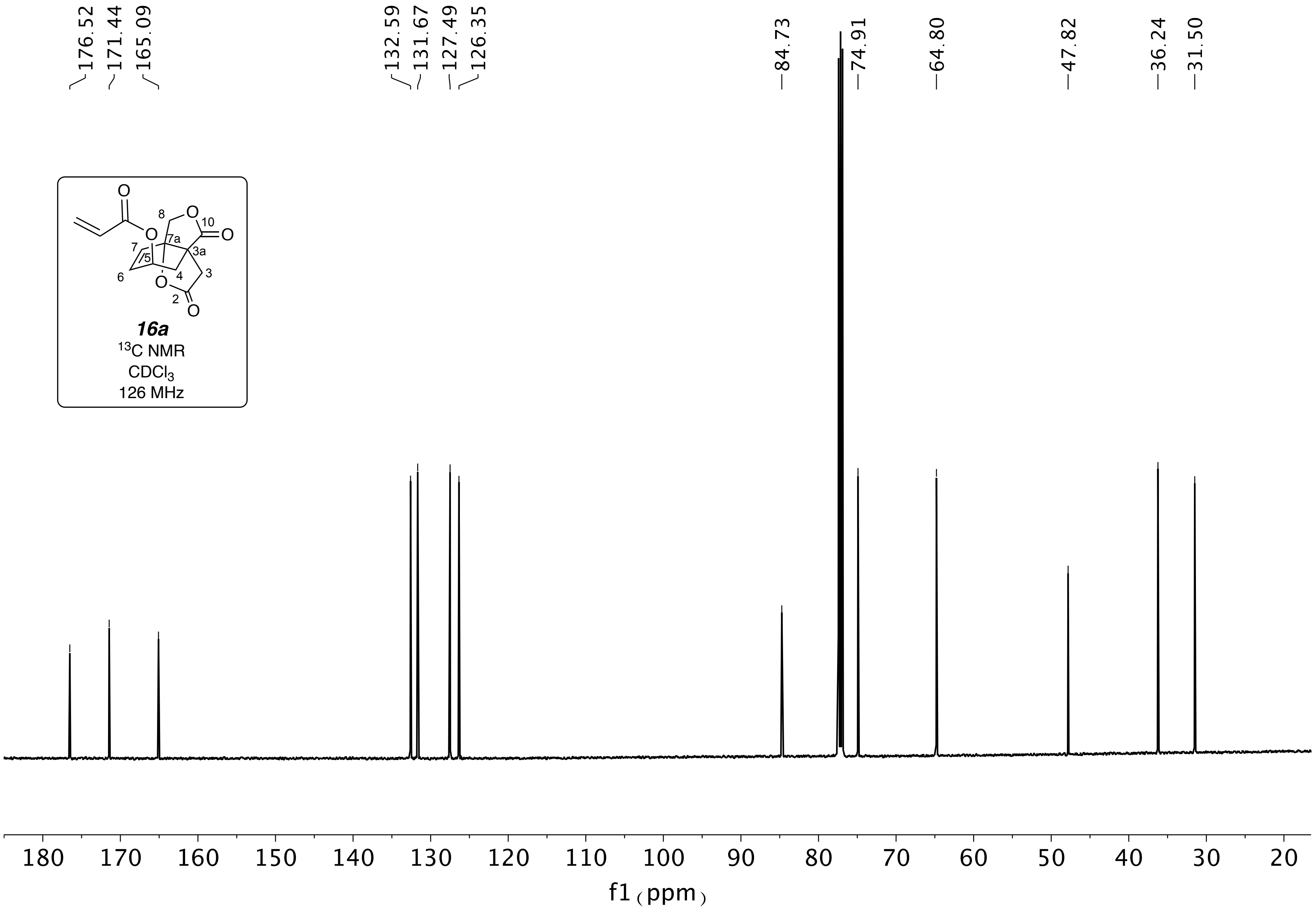
176.52
171.44
165.09
132.59
131.67
127.49
126.35
84.73
74.91
64.80
47.82
36.24
31.50
16a
13C NMR
CDCl3
126 MHz
180 170 160 150 140 130 120 110 100 90 80 70 60 50 40 30 20
f1 (ppm)

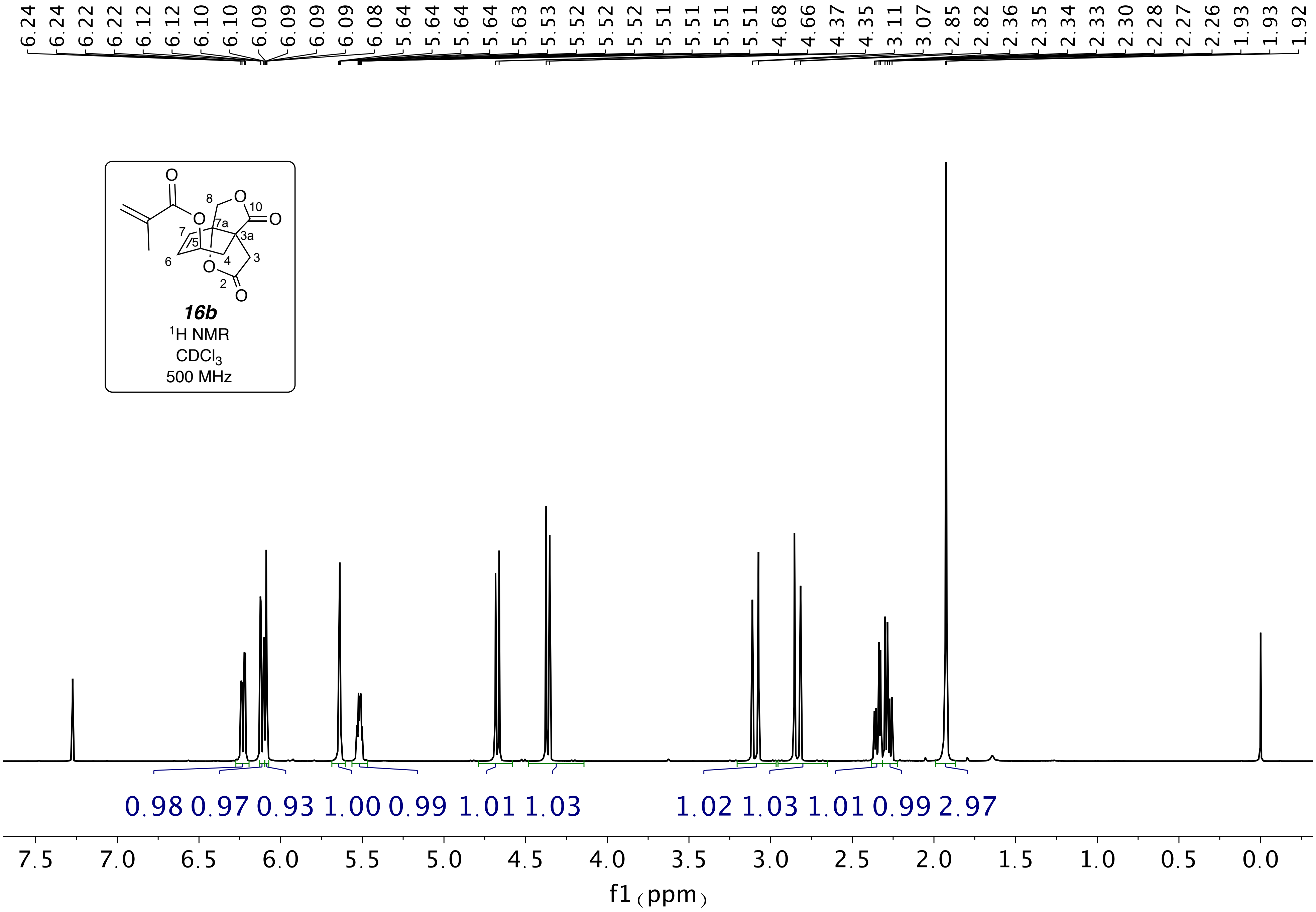
6.24 6.24 6.22 6.22 6.12 6.12 6.10 6.10 6.09 6.09 6.09 6.09 6.08 5.64 5.64 5.64 5.64 5.63 5.53 5.52 5.52 5.52 5.51 5.51 5.51 5.51 4.68 4.66 4.37 4.35 3.11 3.07 2.85 2.82 2.36 2.35 2.34 2.33 2.30 2.28 2.27 2.26 1.93 1.93 1.92
16b
1H NMR
CDCl3
500 MHz
0.98 0.97 0.93 1.00 0.99 1.01 1.03 1.02 1.03 1.01 0.99 2.97
f1 (ppm)

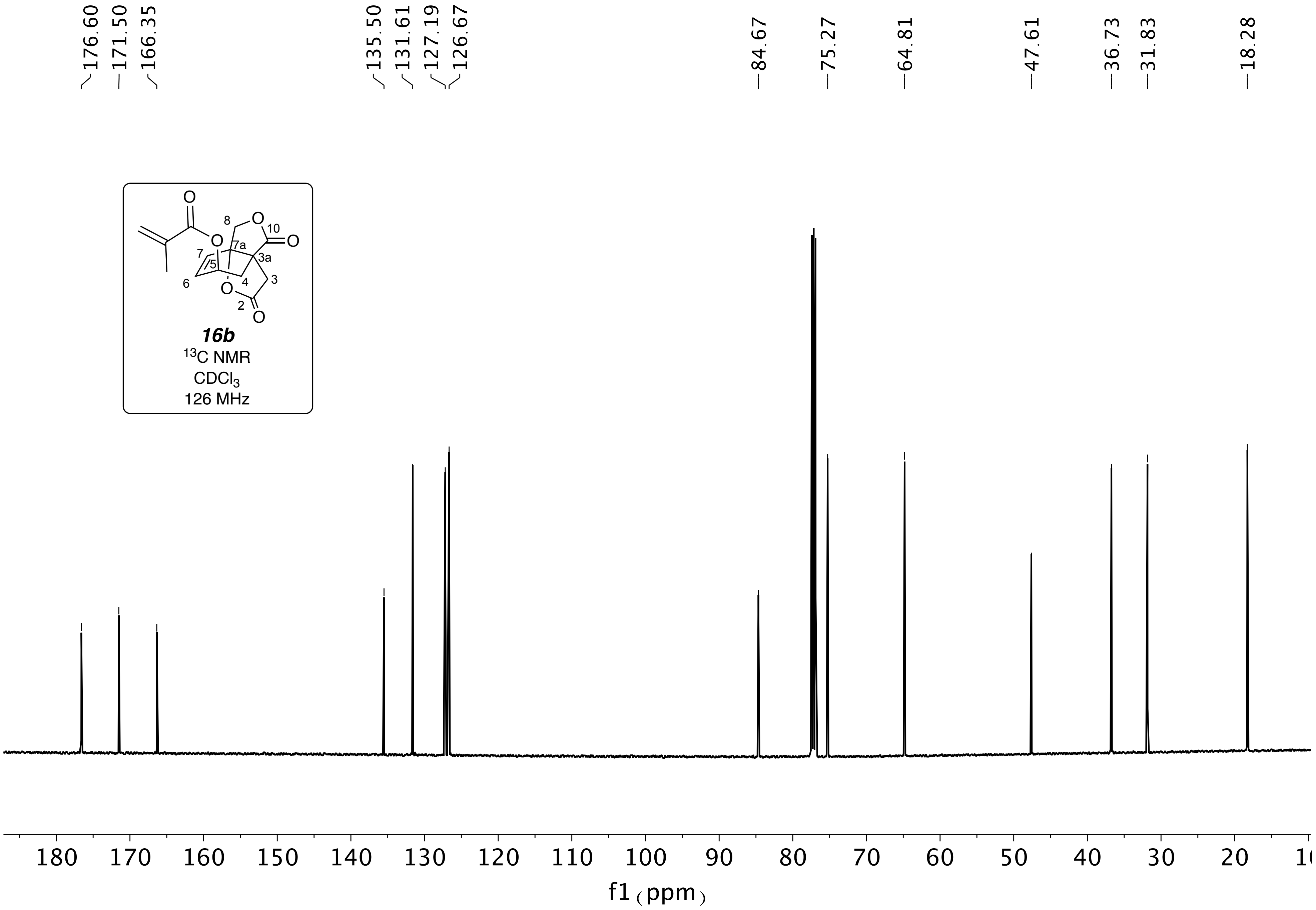
176.60
171.50
166.35
135.50
131.61
127.19
126.67
84.67
75.27
64.81
47.61
36.73
31.83
18.28
16b
13C NMR
CDCl3
126 MHz
180 170 160 150 140 130 120 110 100 90 80 70 60 50 40 30 20
f1 (ppm)

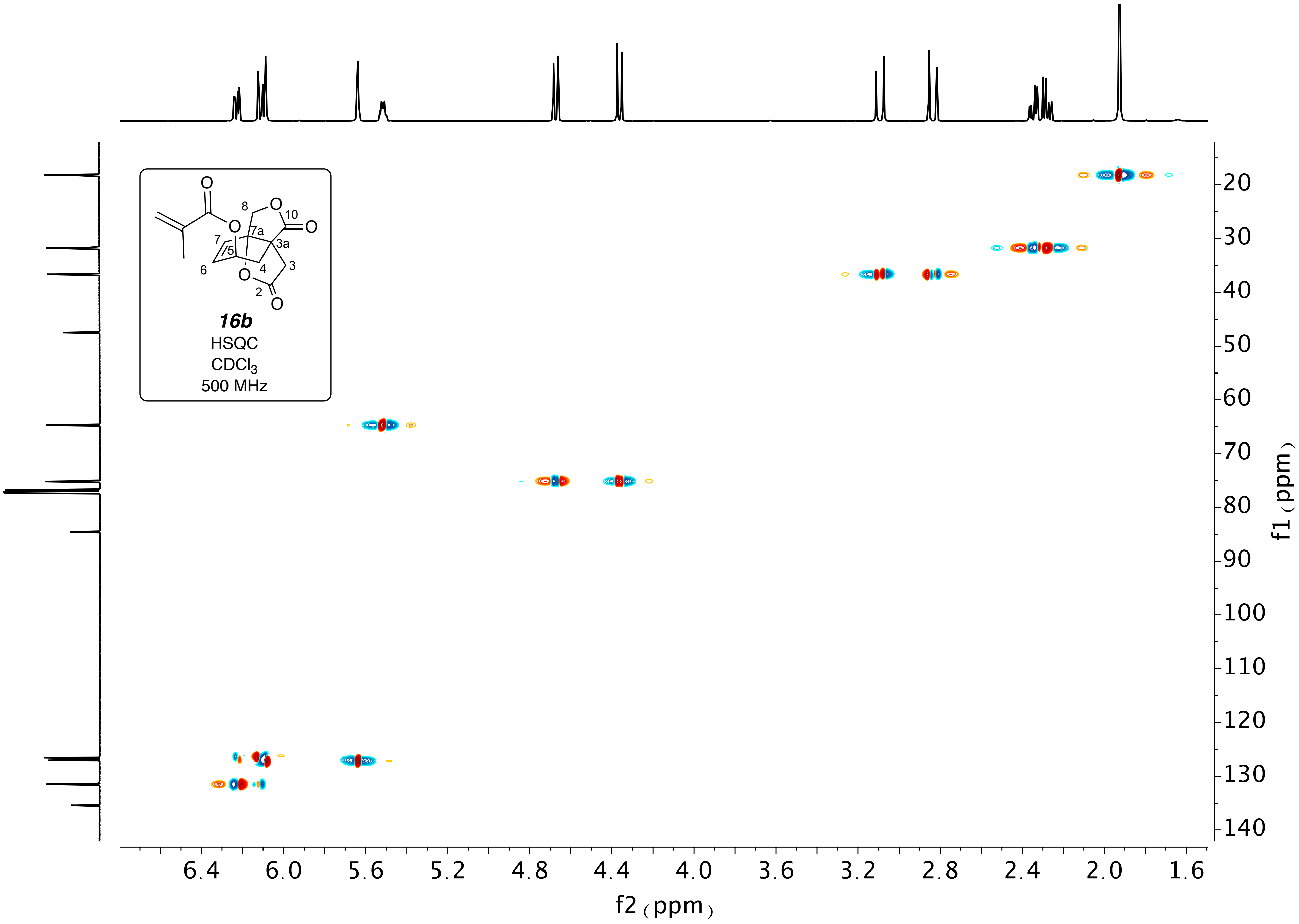
16b
HSQC
$CDCl_3$
500 MHz
6.4
6.0
5.6
5.2
4.8
4.4
4.0
3.6
3.2
2.8
2.4
2.0
1.6
f2 (ppm)
20
30
40
50
60
70
80
90
100
110
120
130
140
f1 (ppm)

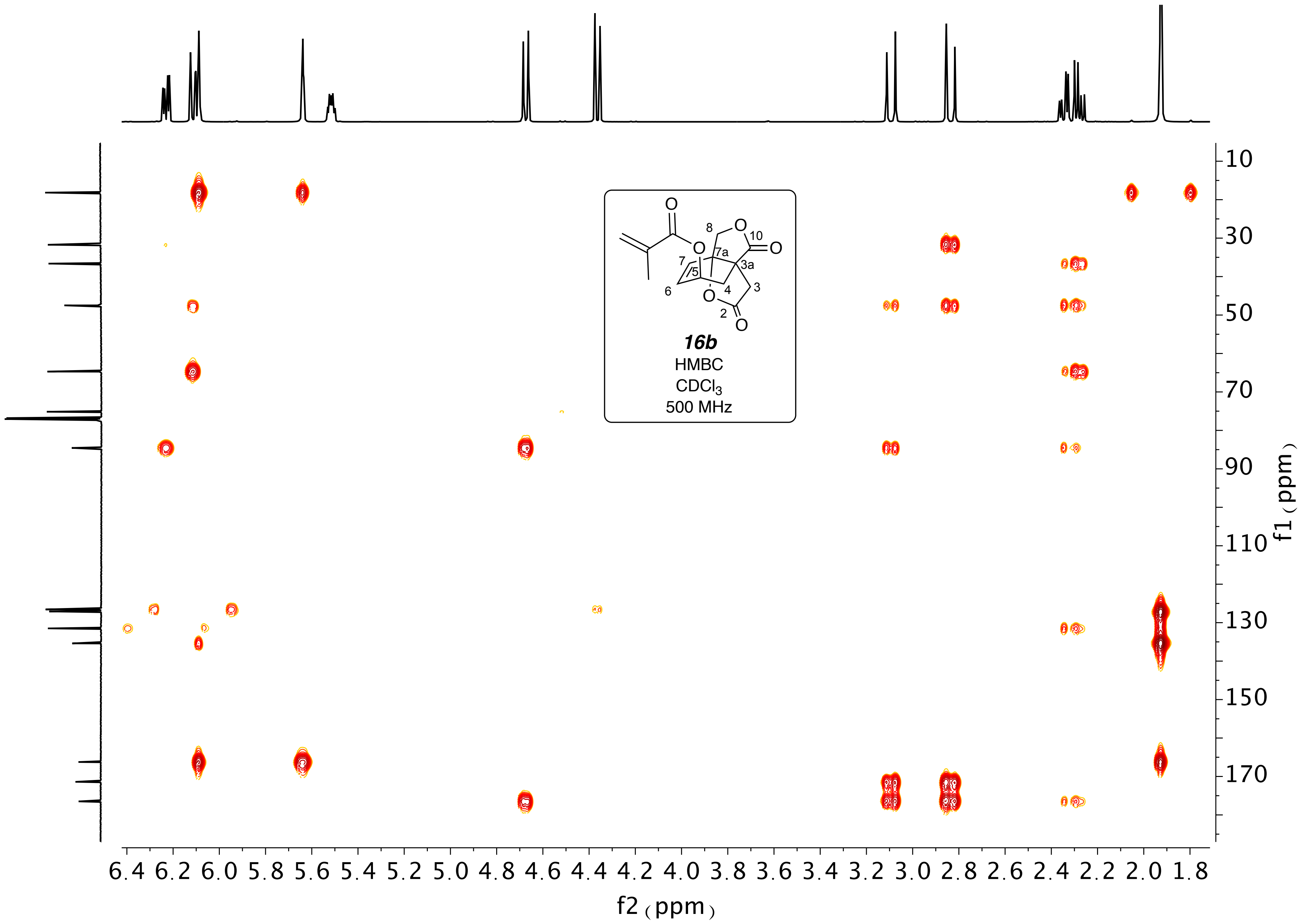

O
8
O
10
O
O
7a
7
5
3a
6
4
3
O
2
O
16b
HMBC
CDCl3
500 MHz
f2 (ppm)
f1 (ppm)

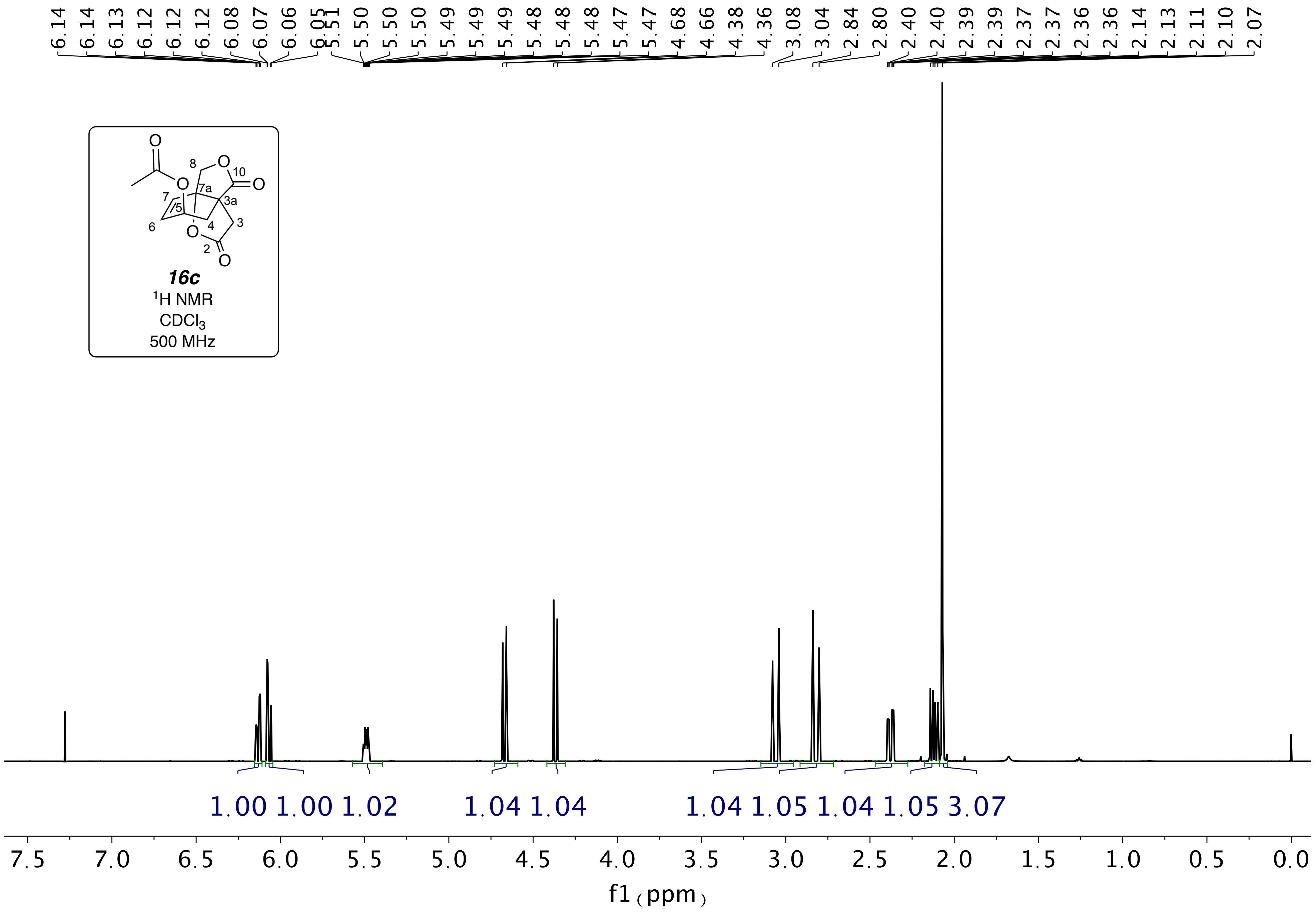
6.14 6.14 6.13 6.12 6.12 6.12 6.08 6.07 6.06 6.05 5.51 5.50 5.50 5.50 5.49 5.49 5.49 5.48 5.48 5.48 5.47 5.47 4.68 4.66 4.38 4.36 3.08 3.04 2.84 2.80 2.40 2.40 2.39 2.39 2.37 2.37 2.36 2.36 2.14 2.13 2.11 2.10 2.07
16c
^{1}H NMR
$CDCl_3$
500 MHz
1.00 1.00 1.02 1.04 1.04 1.04 1.05 1.04 1.05 3.07
7.5 7.0 6.5 6.0 5.5 5.0 4.5 4.0 3.5 3.0 2.5 2.0 1.5 1.0 0.5 0.0
f1 (ppm)

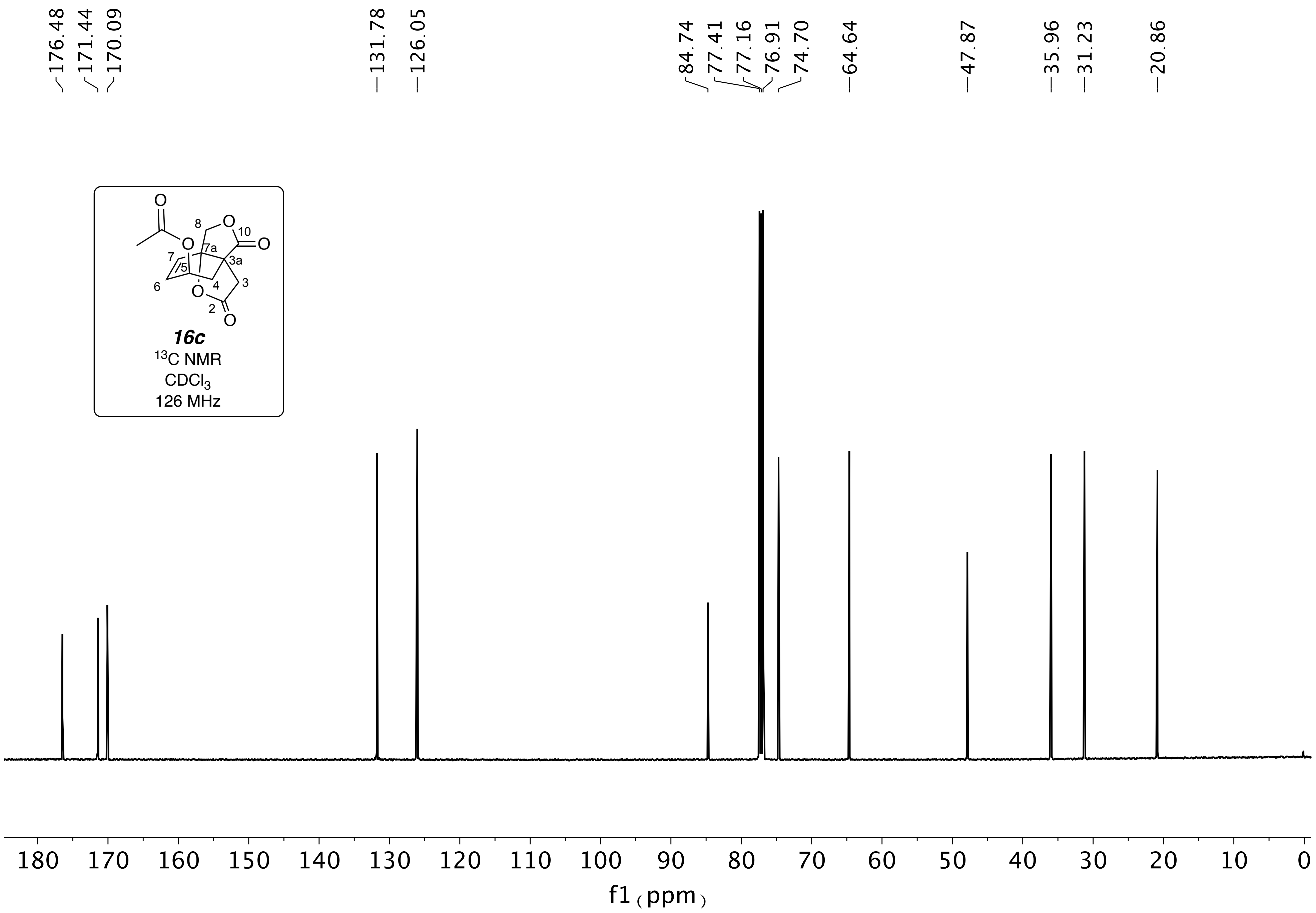

176.48
171.44
170.09
131.78
126.05
84.74
77.41
77.16
76.91
74.70
64.64
47.87
35.96
31.23
20.86
O
8
O
10
O
7
7a
5
3a
6
4
3
O
2
O
16c
13C NMR
CDCl3
126 MHz
180 170 160 150 140 130 120 110 100 90 80 70 60 50 40 30 20 10 0
f1 (ppm)

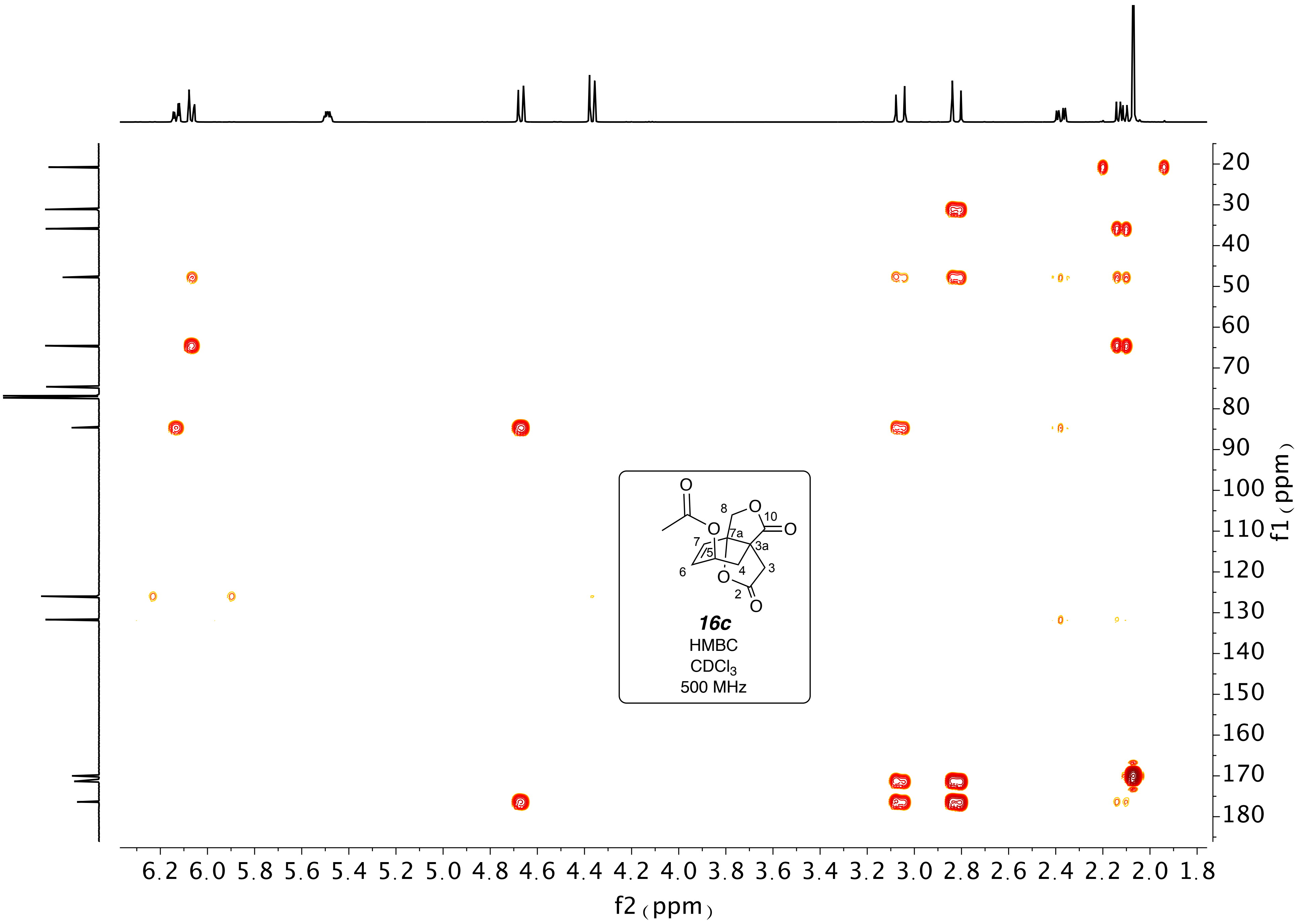
16c
HMBC
$CDCl_3$
500 MHz
f2 (ppm)
f1 (ppm)
6.2 6.0 5.8 5.6 5.4 5.2 5.0 4.8 4.6 4.4 4.2 4.0 3.8 3.6 3.4 3.2 3.0 2.8 2.6 2.4 2.2 2.0 1.8
20 30 40 50 60 70 80 90 100 110 120 130 140 150 160 170 180

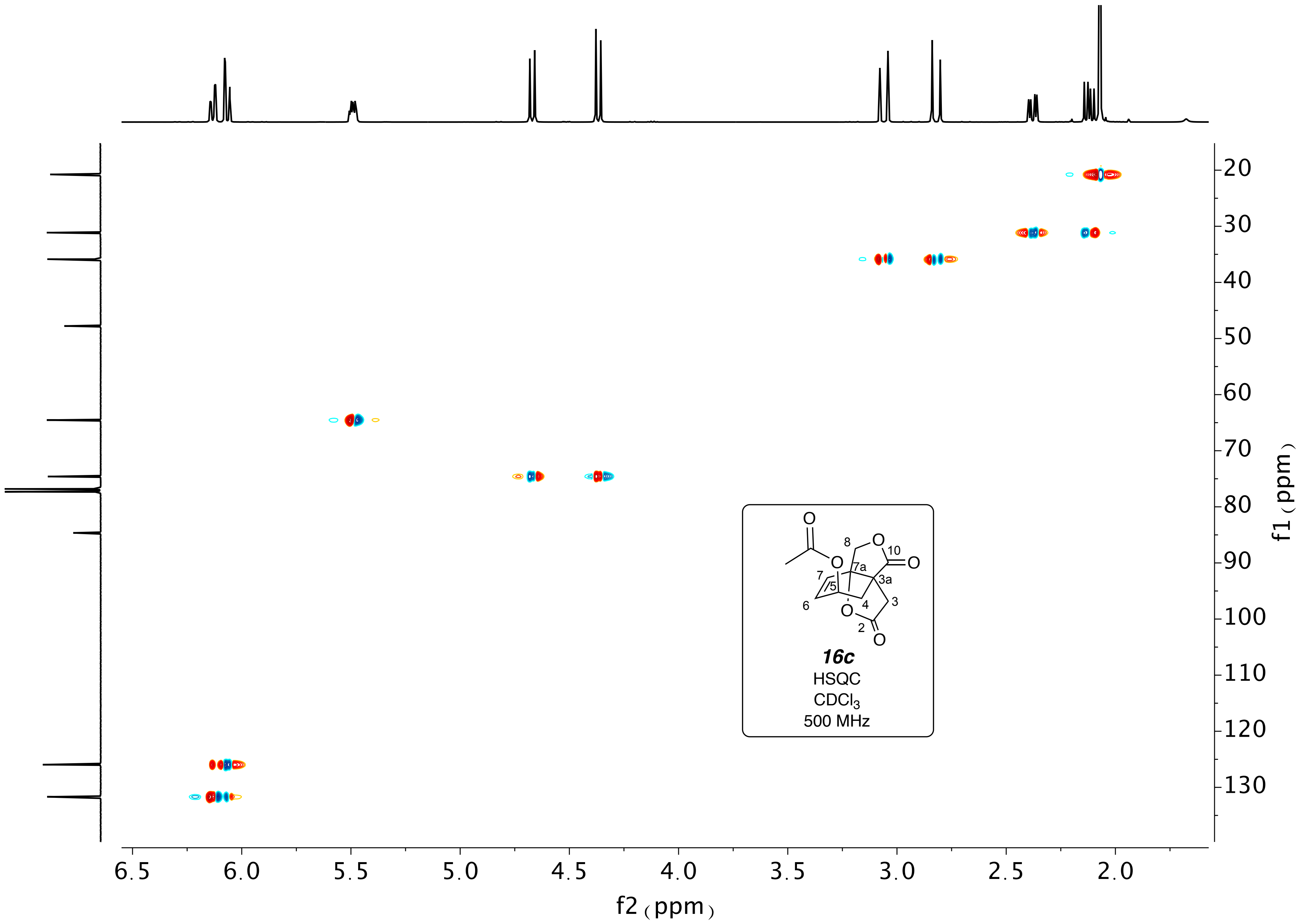
16c
HSQC
$CDCl_3$
500 MHz
8
10
7a
3a
7
5
6
4
3
2
O
f2 (ppm)
f1 (ppm)
6.5
6.0
5.5
5.0
4.5
4.0
3.5
3.0
2.5
2.0
20
30
40
50
60
70
80
90
100
110
120
130

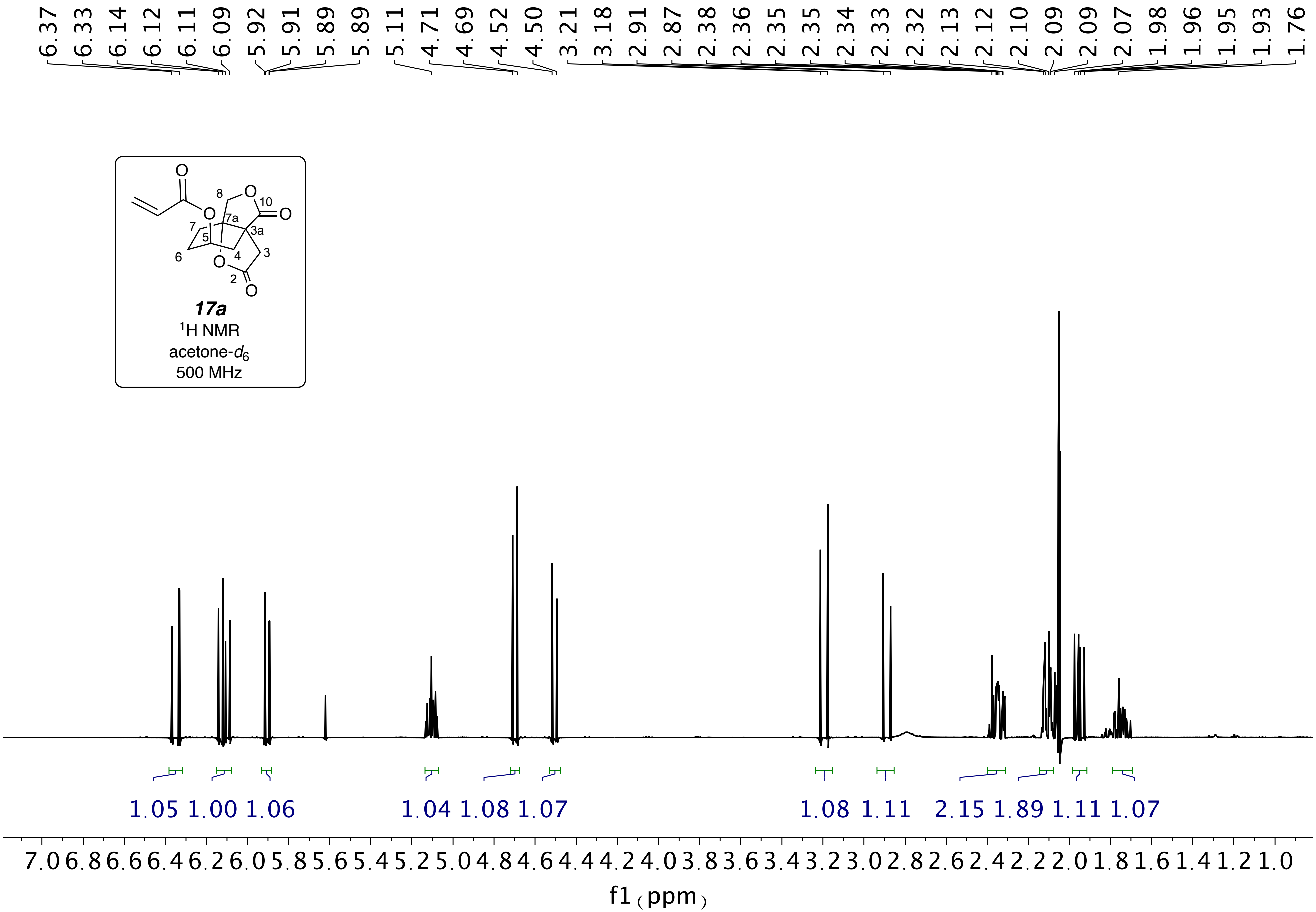
6.37 6.33 6.14 6.12 6.11 6.09 5.92 5.91 5.89 5.89 5.11 4.71 4.69 4.52 4.50 3.21 3.18 2.91 2.87 2.38 2.36 2.35 2.35 2.34 2.33 2.32 2.13 2.12 2.10 2.09 2.09 2.07 1.98 1.96 1.95 1.93 1.76
17a
^{1}H NMR
acetone-d_6
500 MHz
1.05 1.00 1.06 1.04 1.08 1.07 1.08 1.11 2.15 1.89 1.11 1.07
7.0 6.8 6.6 6.4 6.2 6.0 5.8 5.6 5.4 5.2 5.0 4.8 4.6 4.4 4.2 4.0 3.8 3.6 3.4 3.2 3.0 2.8 2.6 2.4 2.2 2.0 1.8 1.6 1.4 1.2 1.0
f1 (ppm)

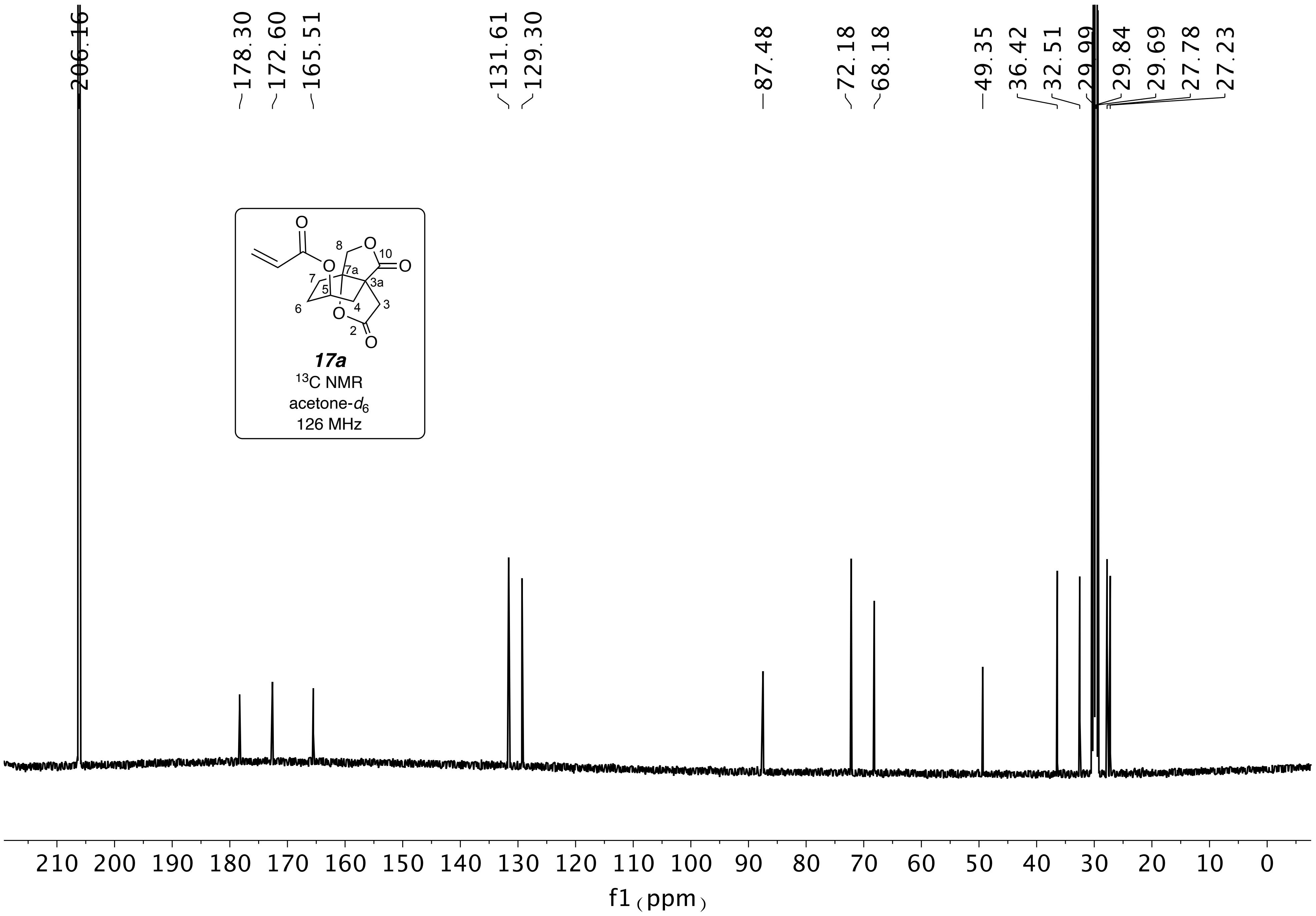
206.16
178.30
172.60
165.51
131.61
129.30
87.48
72.18
68.18
49.35
36.42
32.51
29.99
29.84
29.69
27.78
27.23
17a
^{13}C NMR
acetone-d_6
126 MHz
f1 (ppm)

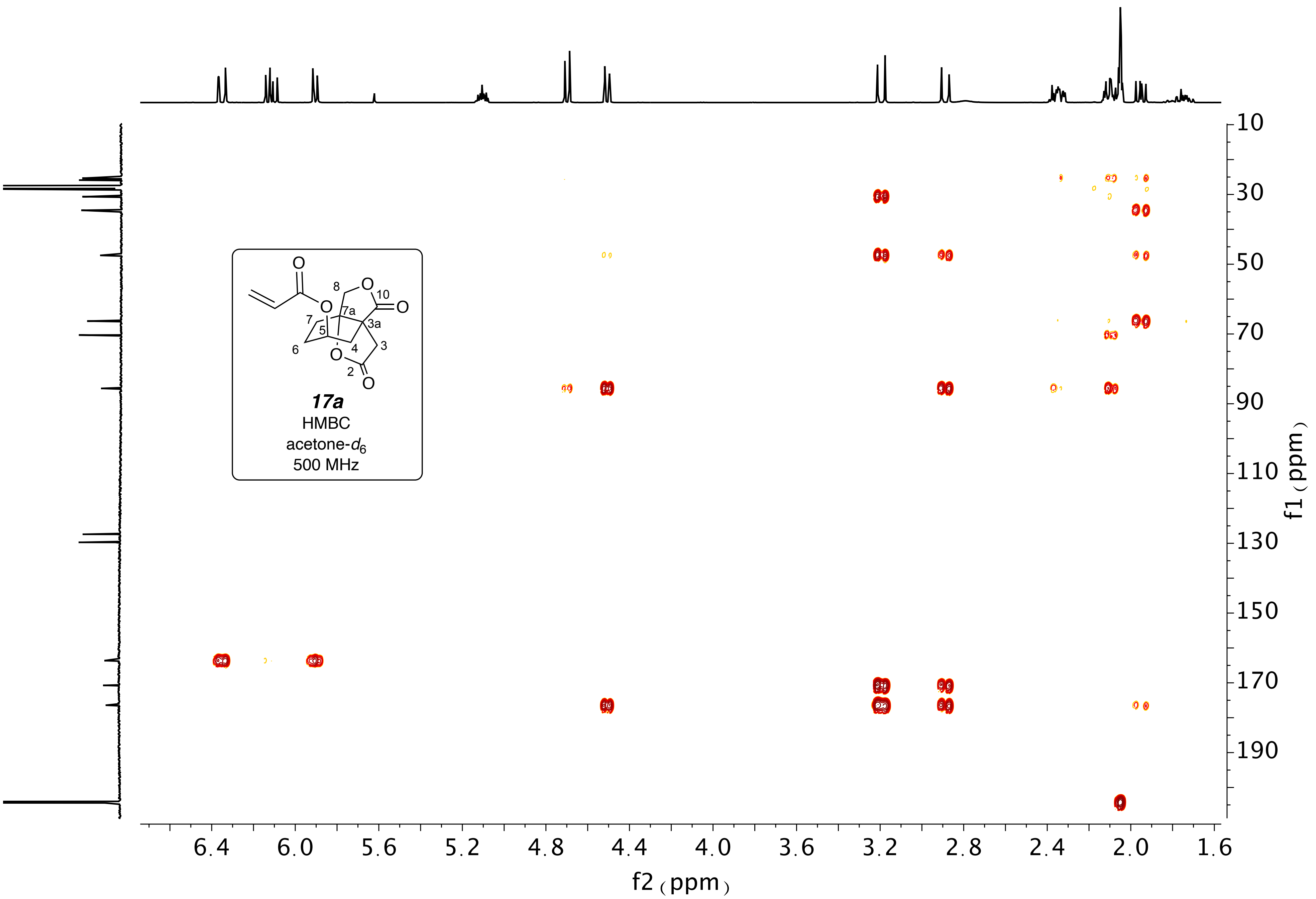
17a
HMBC
acetone-d_6
500 MHz
f2 (ppm)
f1 (ppm)

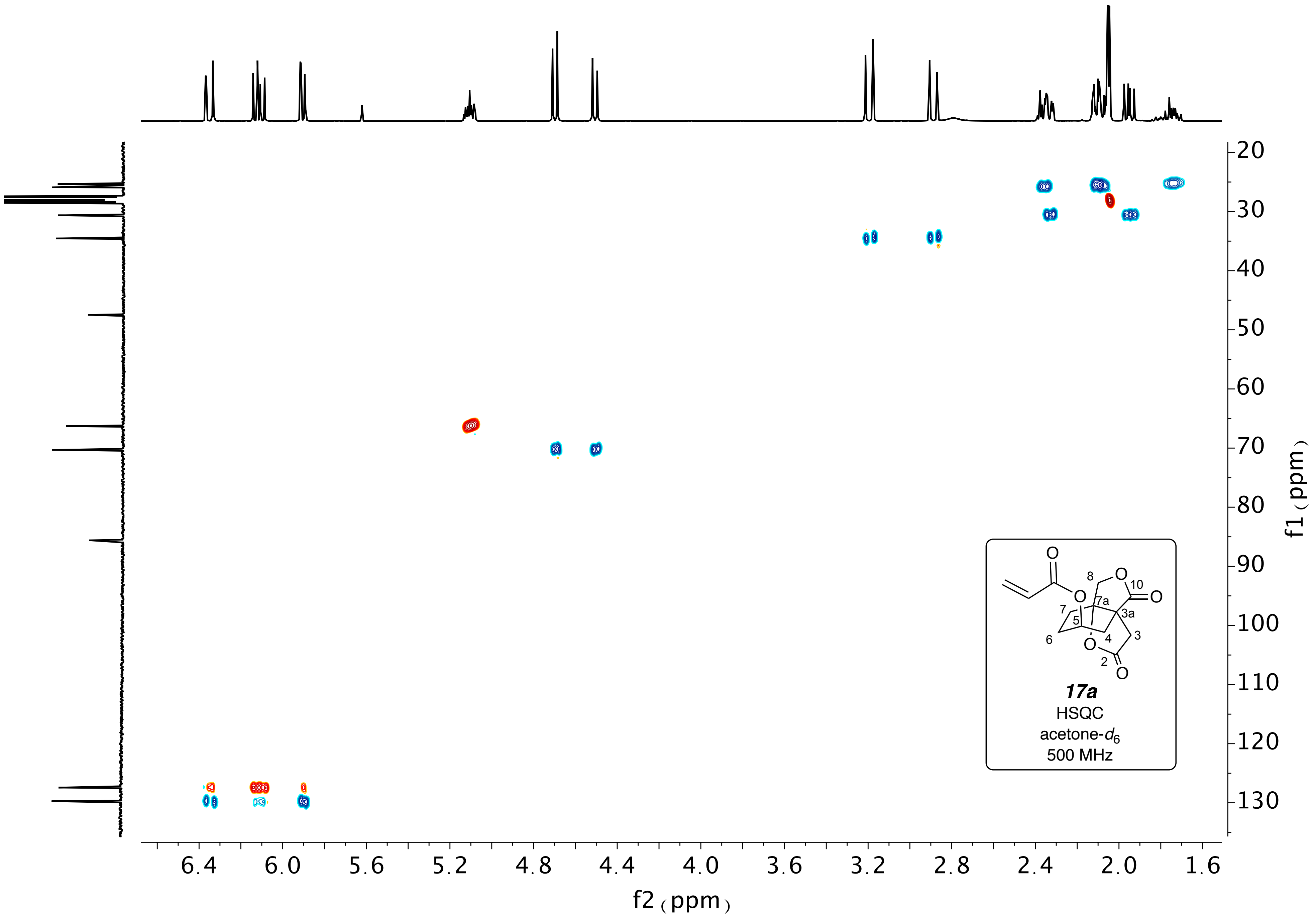

f2 (ppm)
f1 (ppm)
17a
HSQC
acetone-d_6
500 MHz

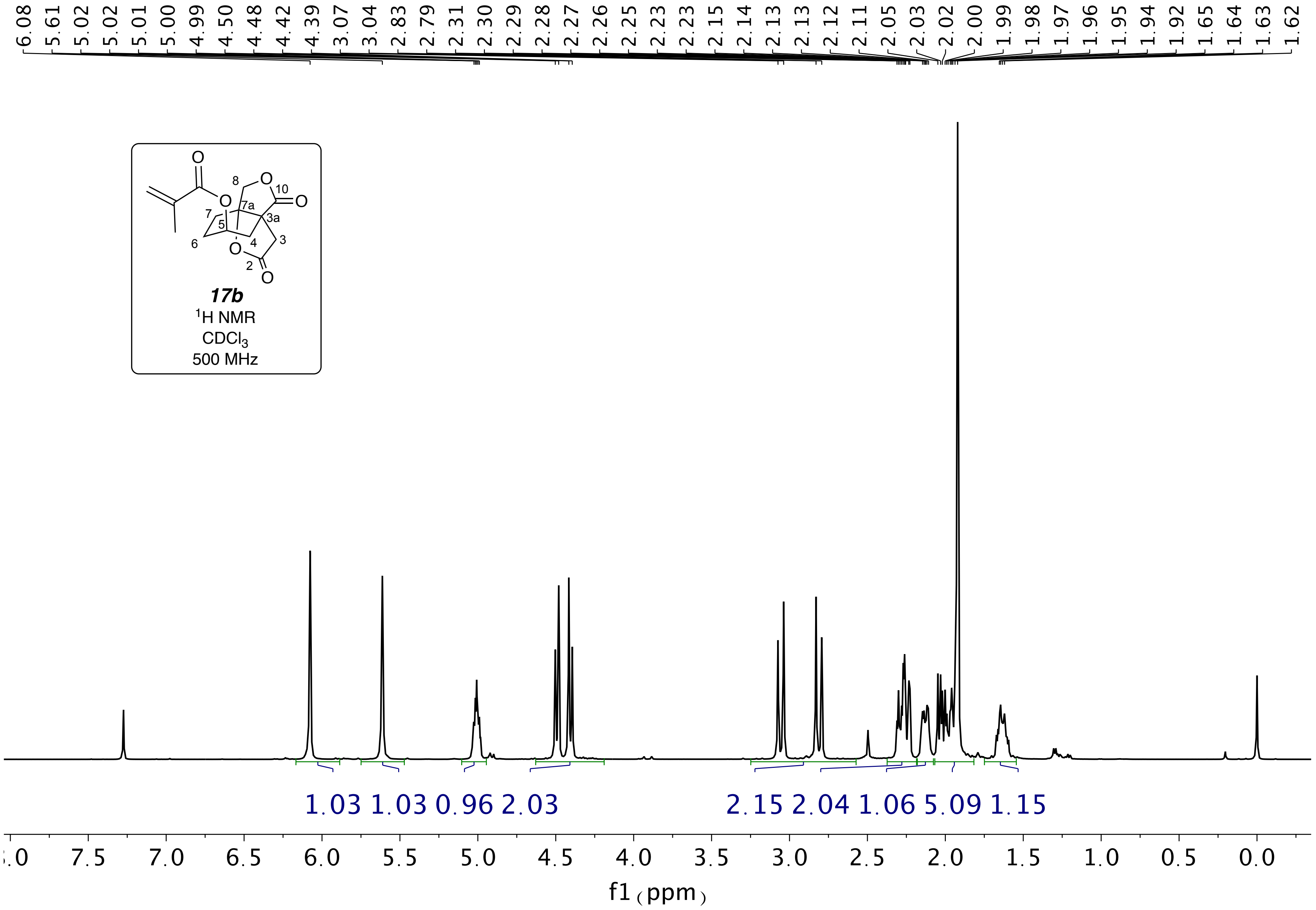
6.08 5.61 5.02 5.02 5.01 5.00 4.99 4.50 4.48 4.42 4.39 3.07 3.04 2.83 2.79 2.31 2.30 2.29 2.28 2.27 2.26 2.25 2.23 2.23 2.15 2.14 2.13 2.13 2.12 2.11 2.05 2.03 2.02 2.00 1.99 1.98 1.97 1.96 1.95 1.94 1.92 1.65 1.64 1.63 1.62
O
8
O
10
O
O
7a
7
5
3a
6
O
4
3
2
O
17b
¹H NMR
CDCl₃
500 MHz
1.03 1.03 0.96 2.03
2.15 2.04 1.06 5.09 1.15
7.5 7.0 6.5 6.0 5.5 5.0 4.5 4.0 3.5 3.0 2.5 2.0 1.5 1.0 0.5 0.0
f1 (ppm)

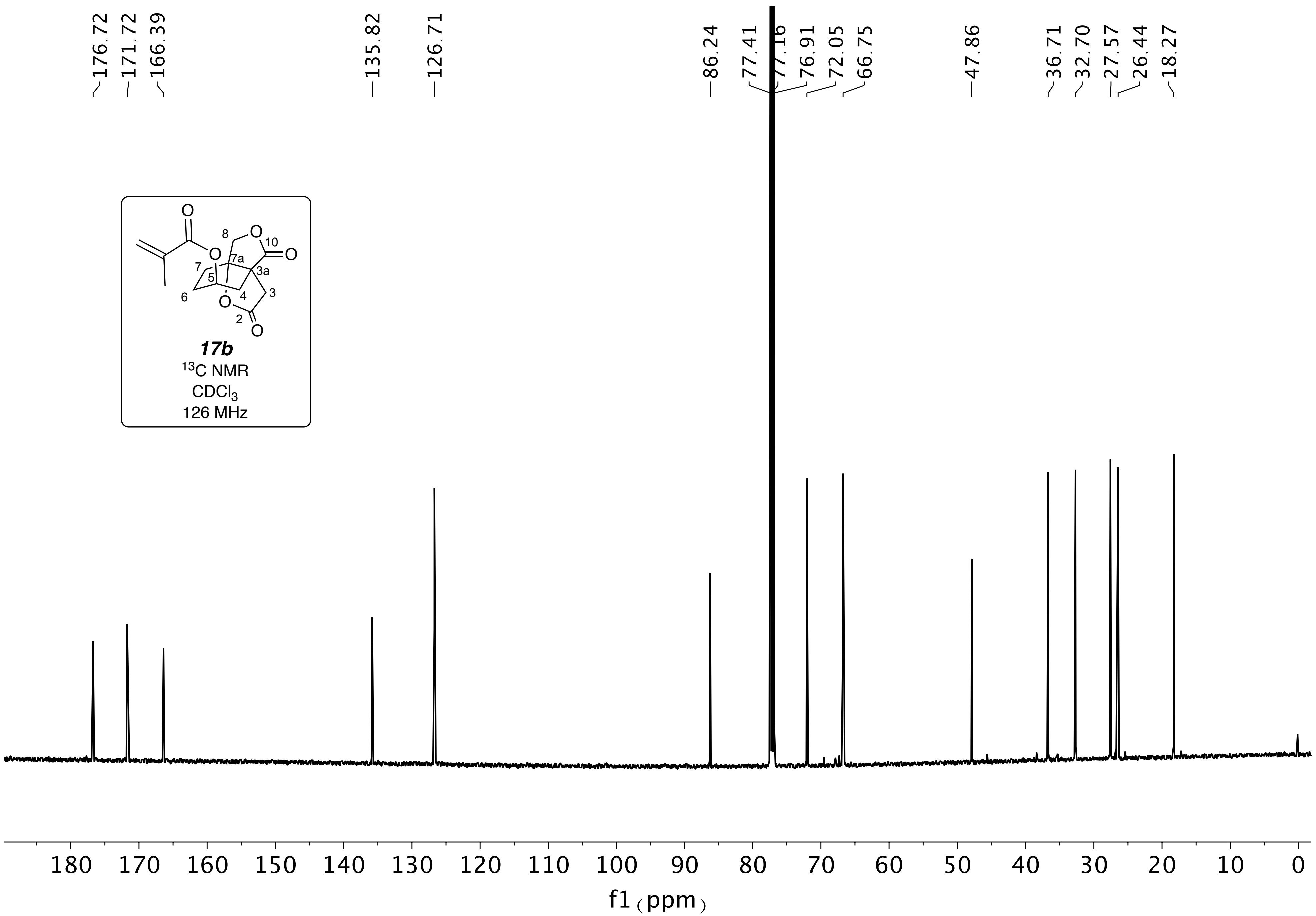
176.72
171.72
166.39
135.82
126.71
86.24
77.41
77.16
76.91
72.05
66.75
47.86
36.71
32.70
27.57
26.44
18.27
17b
13C NMR
CDCl3
126 MHz
180 170 160 150 140 130 120 110 100 90 80 70 60 50 40 30 20 10 0
f1 (ppm)

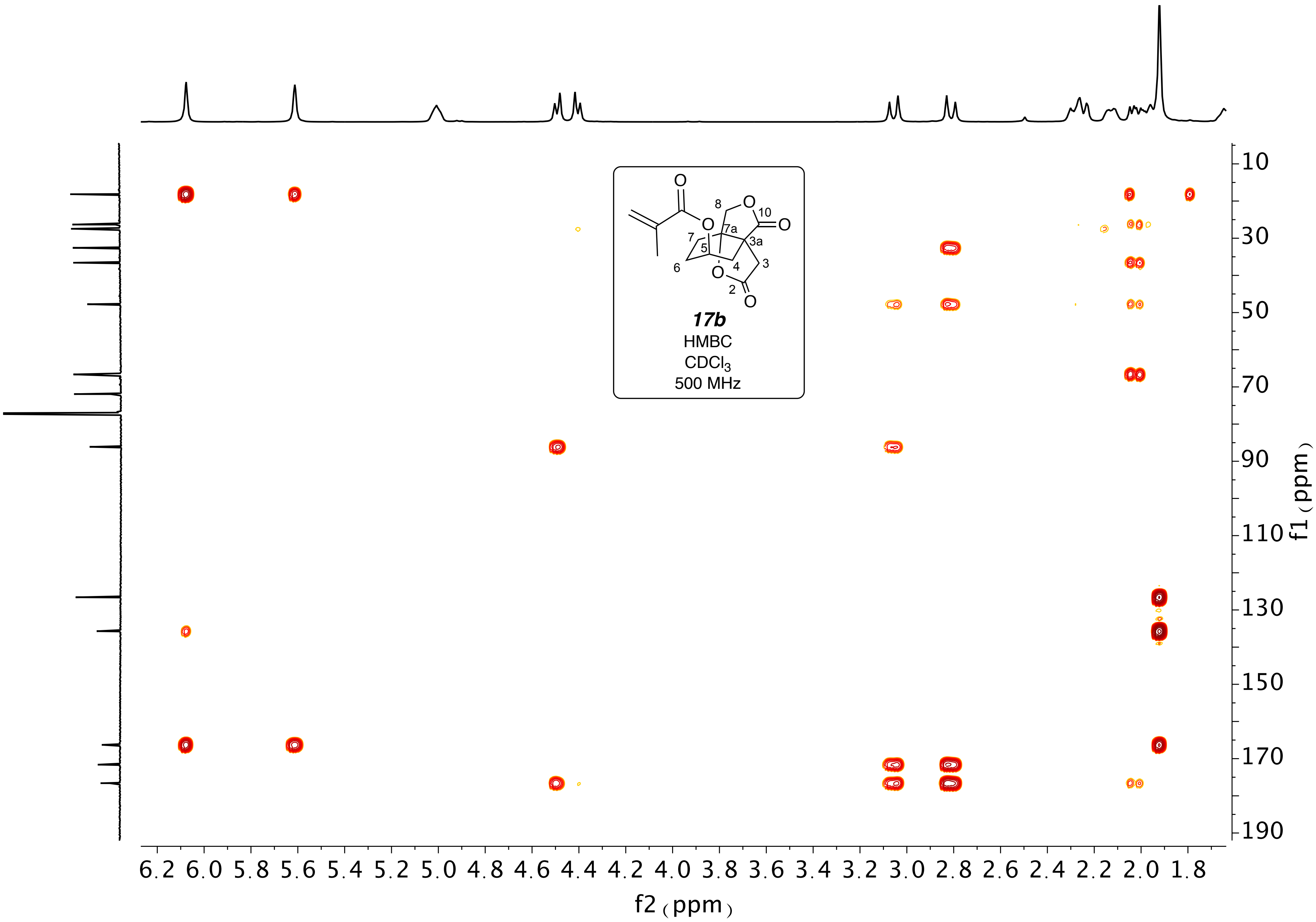
17b
HMBC
$CDCl_3$
500 MHz
f2 (ppm)
f1 (ppm)

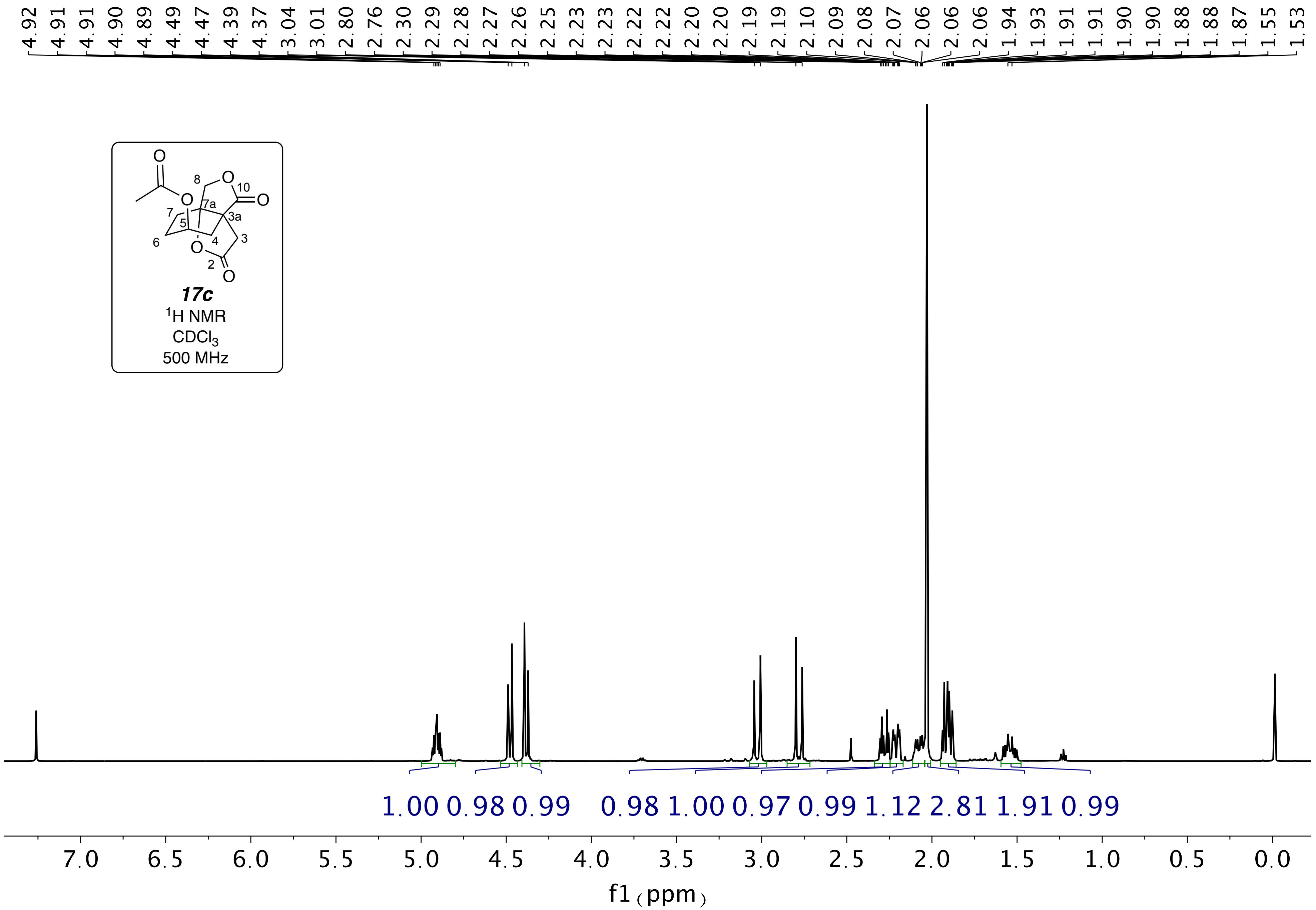
4.92
4.91
4.91
4.90
4.89
4.49
4.47
4.39
4.37
3.04
3.01
2.80
2.76
2.30
2.29
2.28
2.27
2.26
2.25
2.23
2.23
2.22
2.22
2.20
2.20
2.19
2.19
2.10
2.09
2.08
2.07
2.06
2.06
2.06
1.94
1.93
1.91
1.91
1.90
1.90
1.88
1.88
1.87
1.55
1.53
17c
1H NMR
CDCl3
500 MHz
1.00 0.98 0.99 0.98 1.00 0.97 0.99 1.12 2.81 1.91 0.99
7.0 6.5 6.0 5.5 5.0 4.5 4.0 3.5 3.0 2.5 2.0 1.5 1.0 0.5 0.0
f1 (ppm)

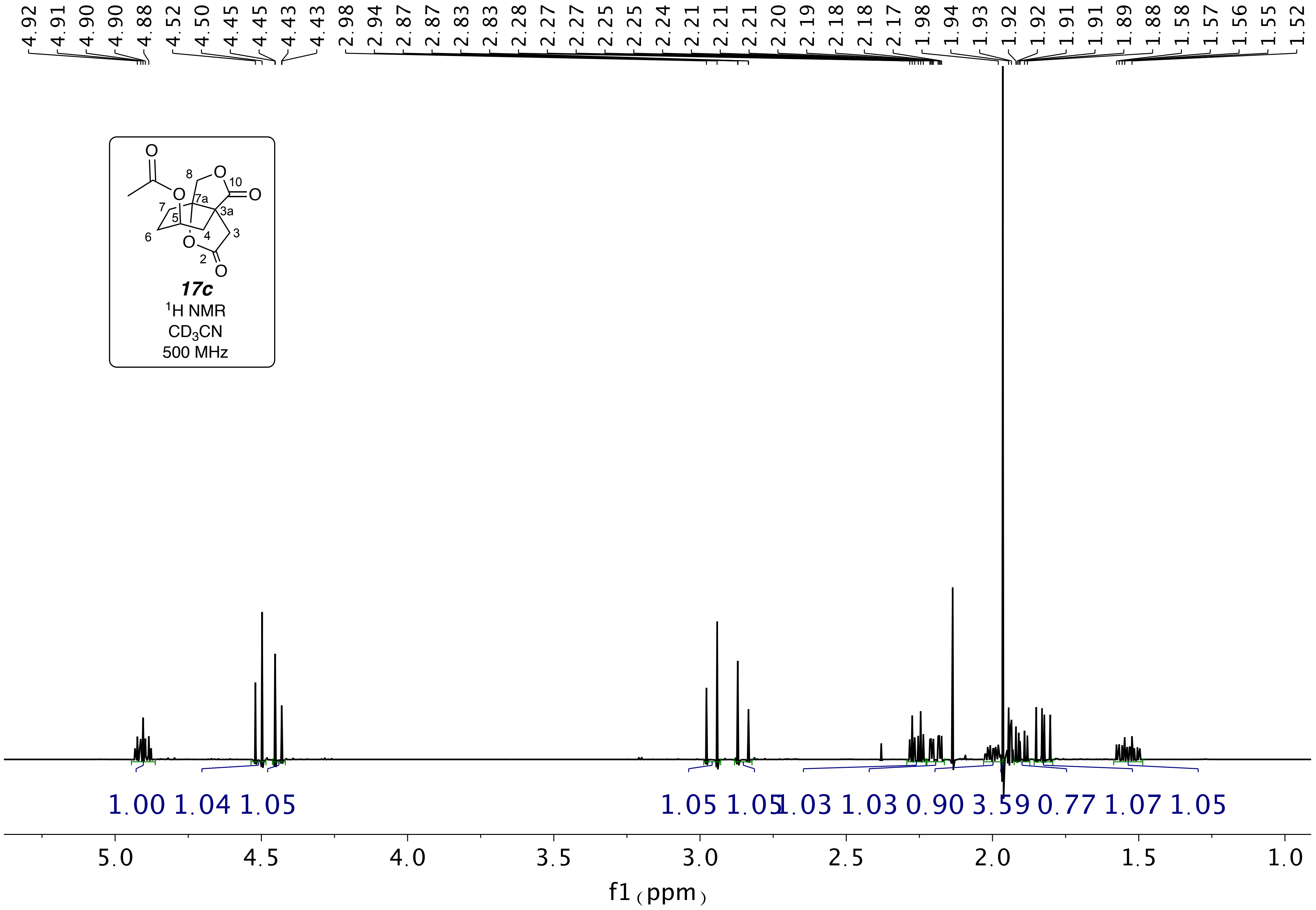
4.92 4.91 4.90 4.90 4.88 4.52 4.50 4.45 4.45 4.43 4.43 2.98 2.94 2.87 2.87 2.83 2.83 2.28 2.27 2.27 2.25 2.25 2.24 2.21 2.21 2.21 2.20 2.19 2.18 2.18 2.17 1.98 1.94 1.93 1.92 1.92 1.91 1.91 1.89 1.88 1.58 1.57 1.56 1.55 1.52
17c
1H NMR
CD3CN
500 MHz
1.00 1.04 1.05 1.05 1.05 1.03 1.03 0.90 3.59 0.77 1.07 1.05
5.0 4.5 4.0 3.5 3.0 2.5 2.0 1.5 1.0
f1 (ppm)

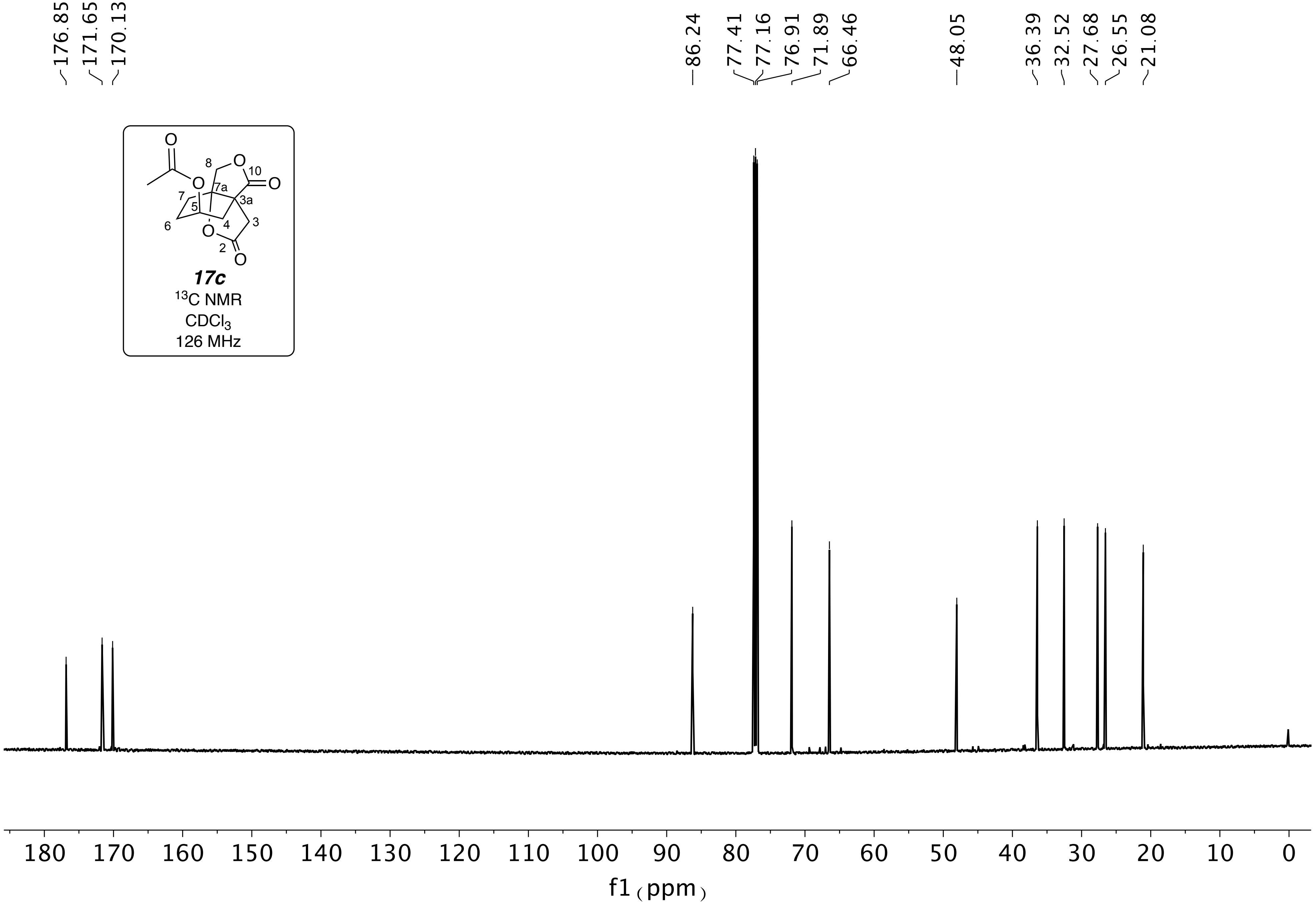
176.85
171.65
170.13
86.24
77.41
77.16
76.91
71.89
66.46
48.05
36.39
32.52
27.68
26.55
21.08
O
8
O
10
O
O
7
7a
3a
5
6
4
3
O
2
O
17c
13C NMR
CDCl3
126 MHz
180 170 160 150 140 130 120 110 100 90 80 70 60 50 40 30 20 10 0
f1 (ppm)

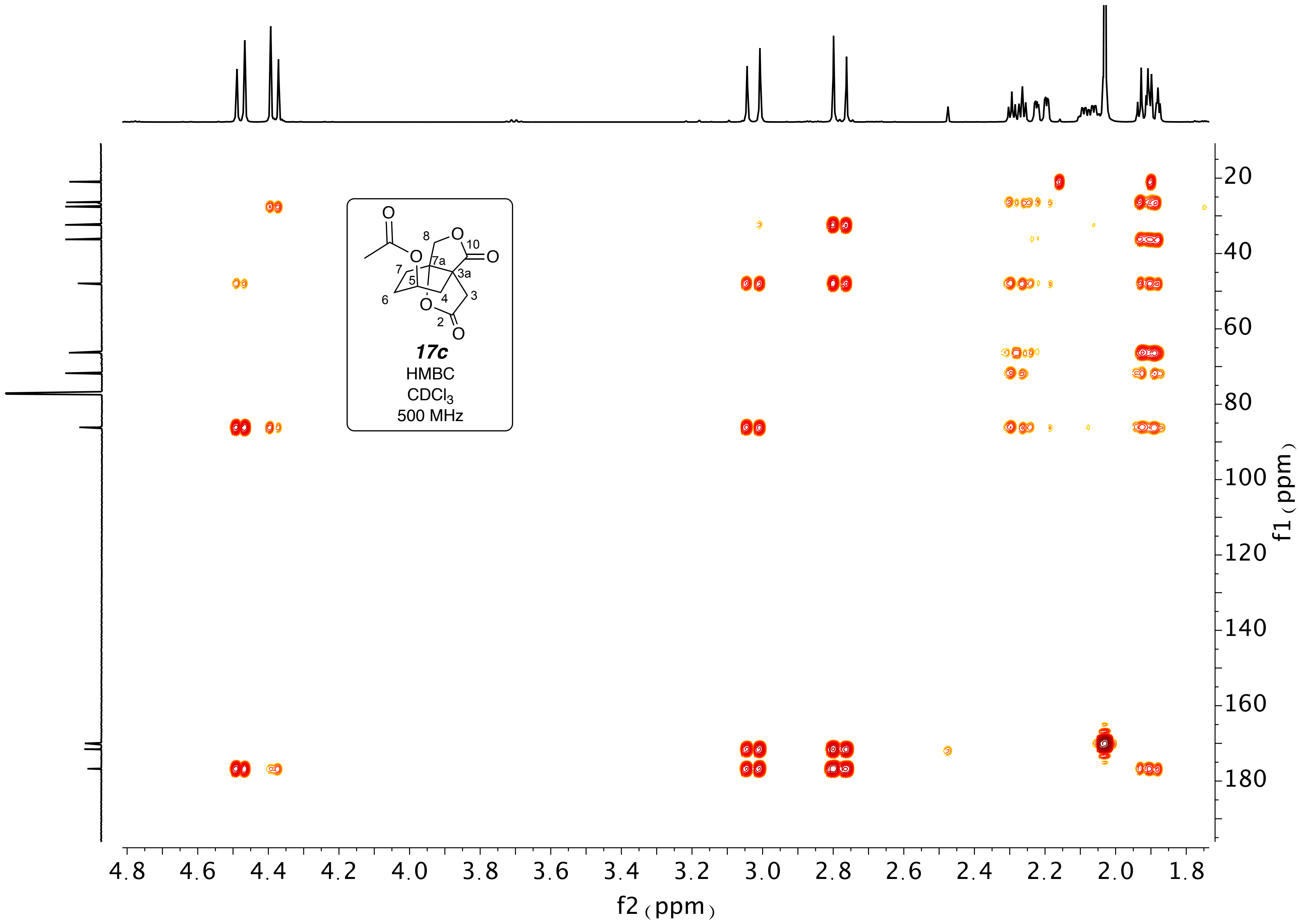
O
8
O
10
O
7a
7
O
3a
5
6
4
3
O
2
O
17c
HMBC
CDCl3
500 MHz
20
40
60
80
100
120
140
160
180
f1 (ppm)
4.8
4.6
4.4
4.2
4.0
3.8
3.6
3.4
3.2
3.0
2.8
2.6
2.4
2.2
2.0
1.8
f2 (ppm)

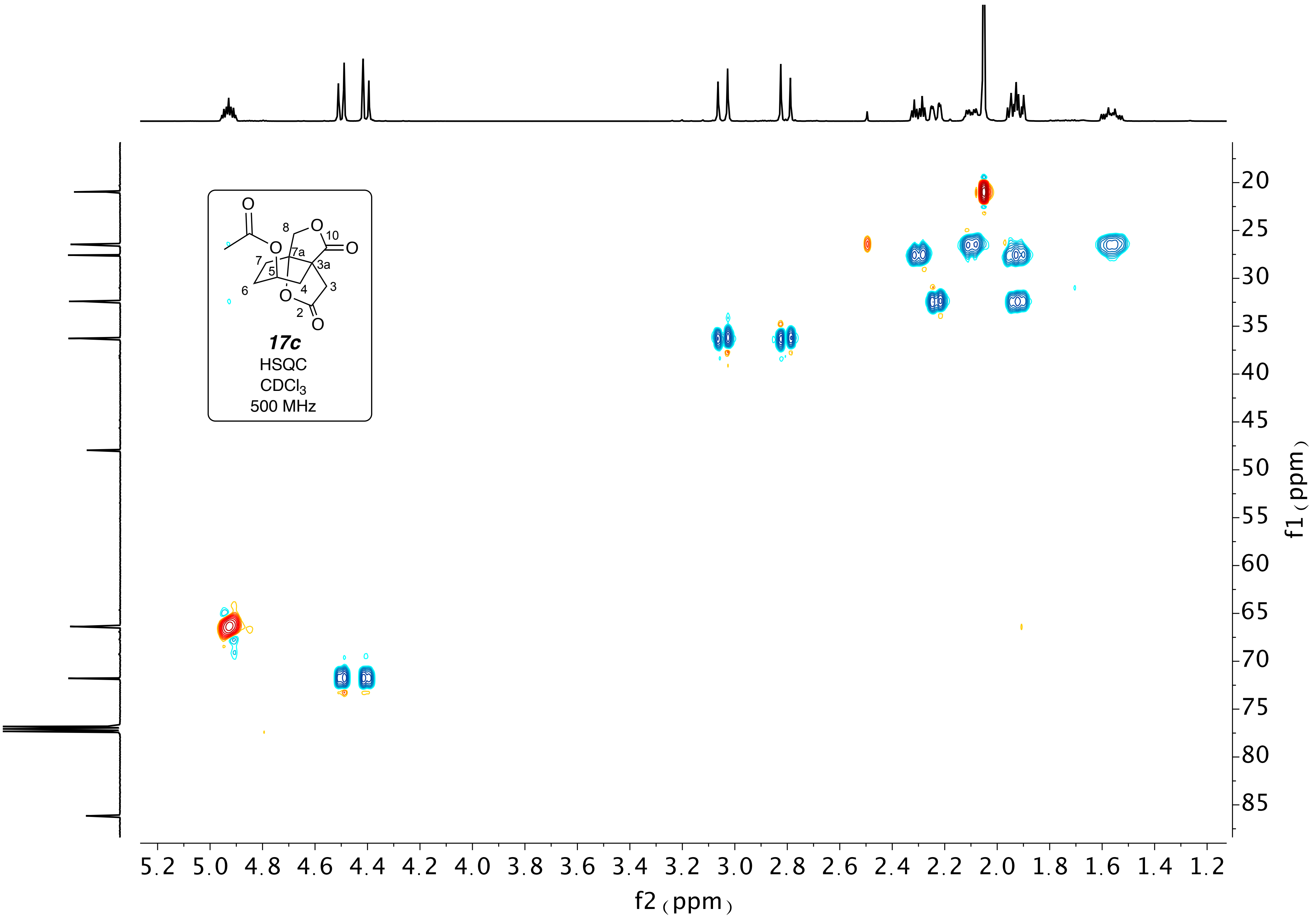

17c
HSQC
CDCl3
500 MHz
f2 (ppm)
f1 (ppm)

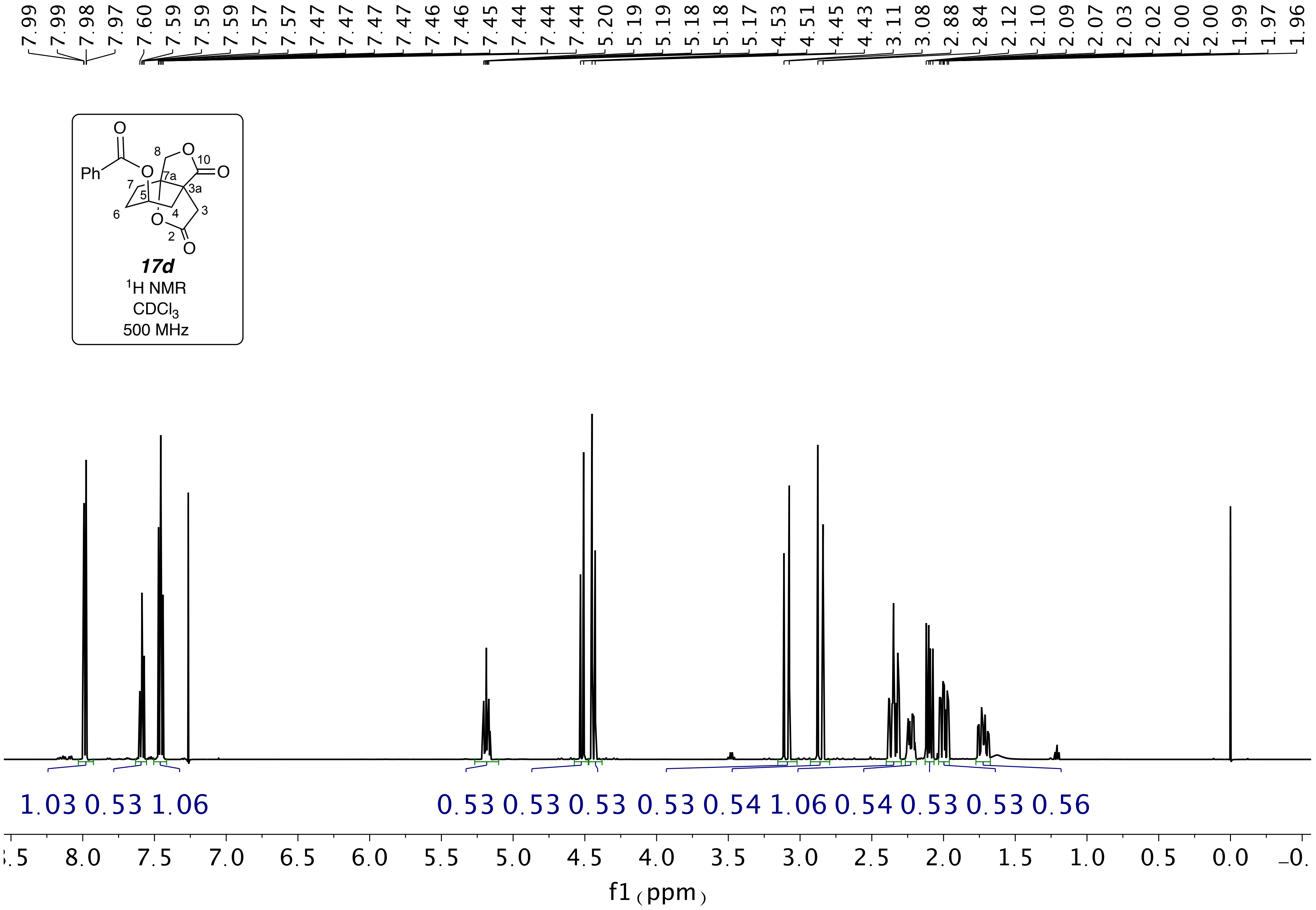
7.99 7.99 7.98 7.97 7.60 7.59 7.59 7.59 7.57 7.57 7.47 7.47 7.47 7.47 7.46 7.46 7.45 7.44 7.44 7.44 5.20 5.19 5.19 5.18 5.18 5.17 4.53 4.51 4.45 4.43 3.11 3.08 2.88 2.84 2.12 2.10 2.09 2.07 2.03 2.02 2.00 2.00 1.99 1.97 1.96
17d
1H NMR
CDCl3
500 MHz
1.03 0.53 1.06 0.53 0.53 0.53 0.53 0.54 1.06 0.54 0.53 0.53 0.56
f1 (ppm)

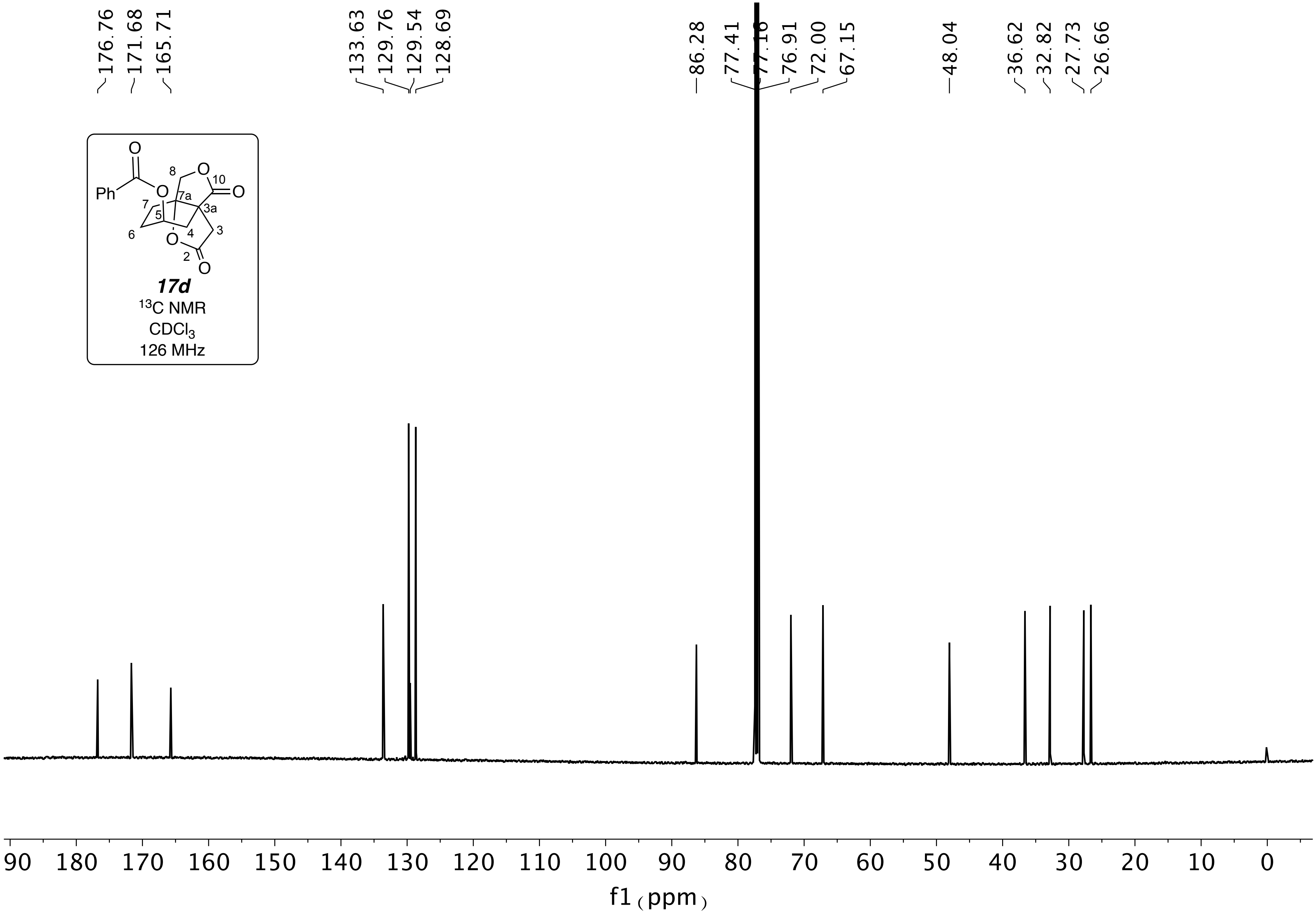

176.76
171.68
165.71
133.63
129.76
129.54
128.69
86.28
77.41
77.16
76.91
72.00
67.15
48.04
36.62
32.82
27.73
26.66
O
Ph
O
8
O
10
O
7
7a
3a
5
6
4
3
O
2
O
17d
^{13}C NMR
$CDCl_3$
126 MHz
190 180 170 160 150 140 130 120 110 100 90 80 70 60 50 40 30 20 10 0
f1 (ppm)

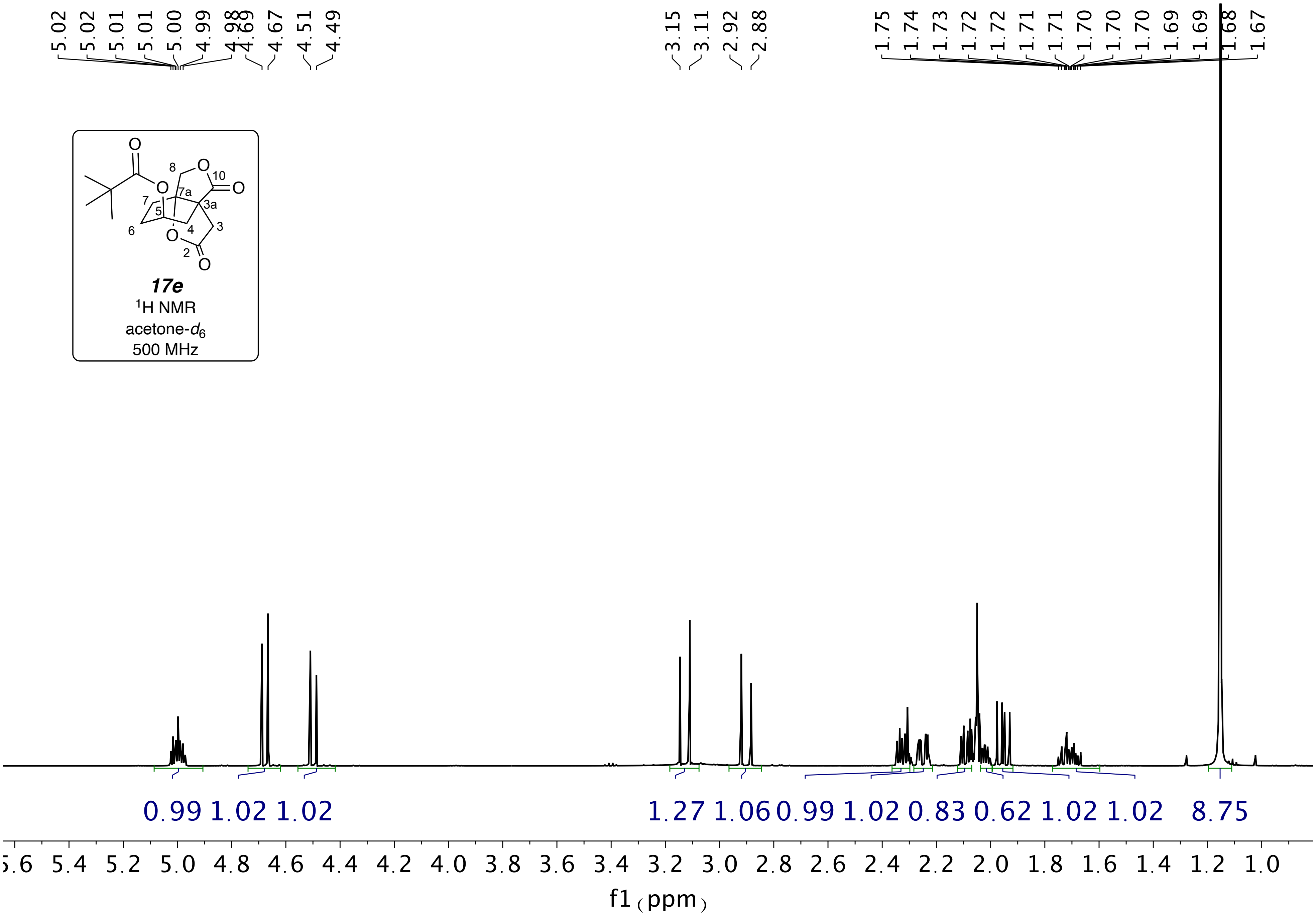
5.02
5.02
5.01
5.01
5.00
4.99
4.98
4.69
4.67
4.51
4.49
3.15
3.11
2.92
2.88
1.75
1.74
1.73
1.72
1.72
1.71
1.71
1.70
1.70
1.70
1.69
1.69
1.68
1.67
O
8
O
10
O
O
7a
7
3a
5
6
4
3
O
2
O
17e
^{1}H NMR
acetone-d_6
500 MHz
0.99
1.02
1.02
1.27
1.06
0.99
1.02
0.83
0.62
1.02
1.02
8.75
5.6
5.4
5.2
5.0
4.8
4.6
4.4
4.2
4.0
3.8
3.6
3.4
3.2
3.0
2.8
2.6
2.4
2.2
2.0
1.8
1.6
1.4
1.2
1.0
f1 (ppm)

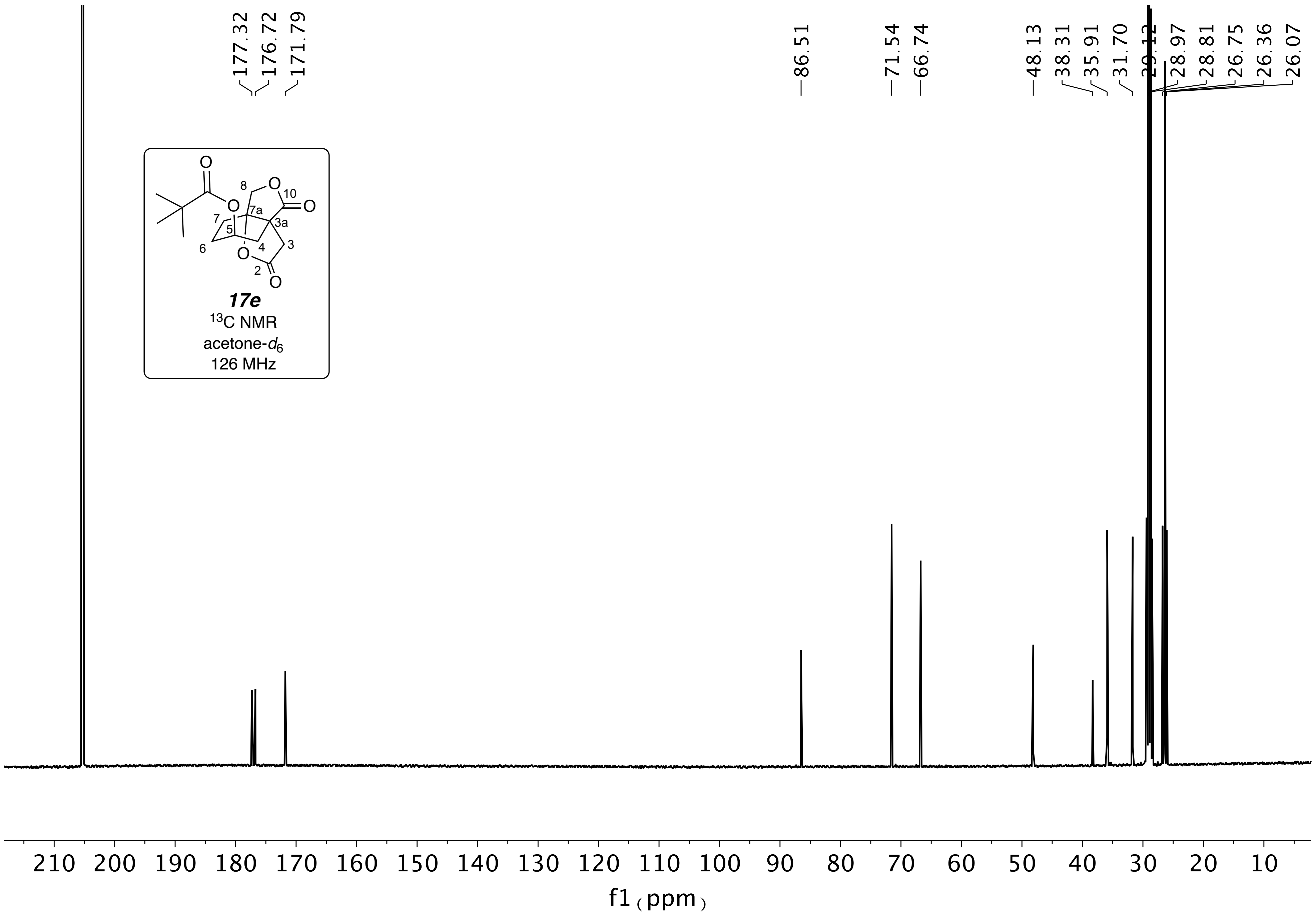
177.32
176.72
171.79
86.51
71.54
66.74
48.13
38.31
35.91
31.70
29.12
28.97
28.81
26.75
26.36
26.07
O
8
O
10
O
O
7a
3a
7
5
6
4
3
O
2
O
17e
13C NMR
acetone-d6
126 MHz
210 200 190 180 170 160 150 140 130 120 110 100 90 80 70 60 50 40 30 20 10
f1 (ppm)

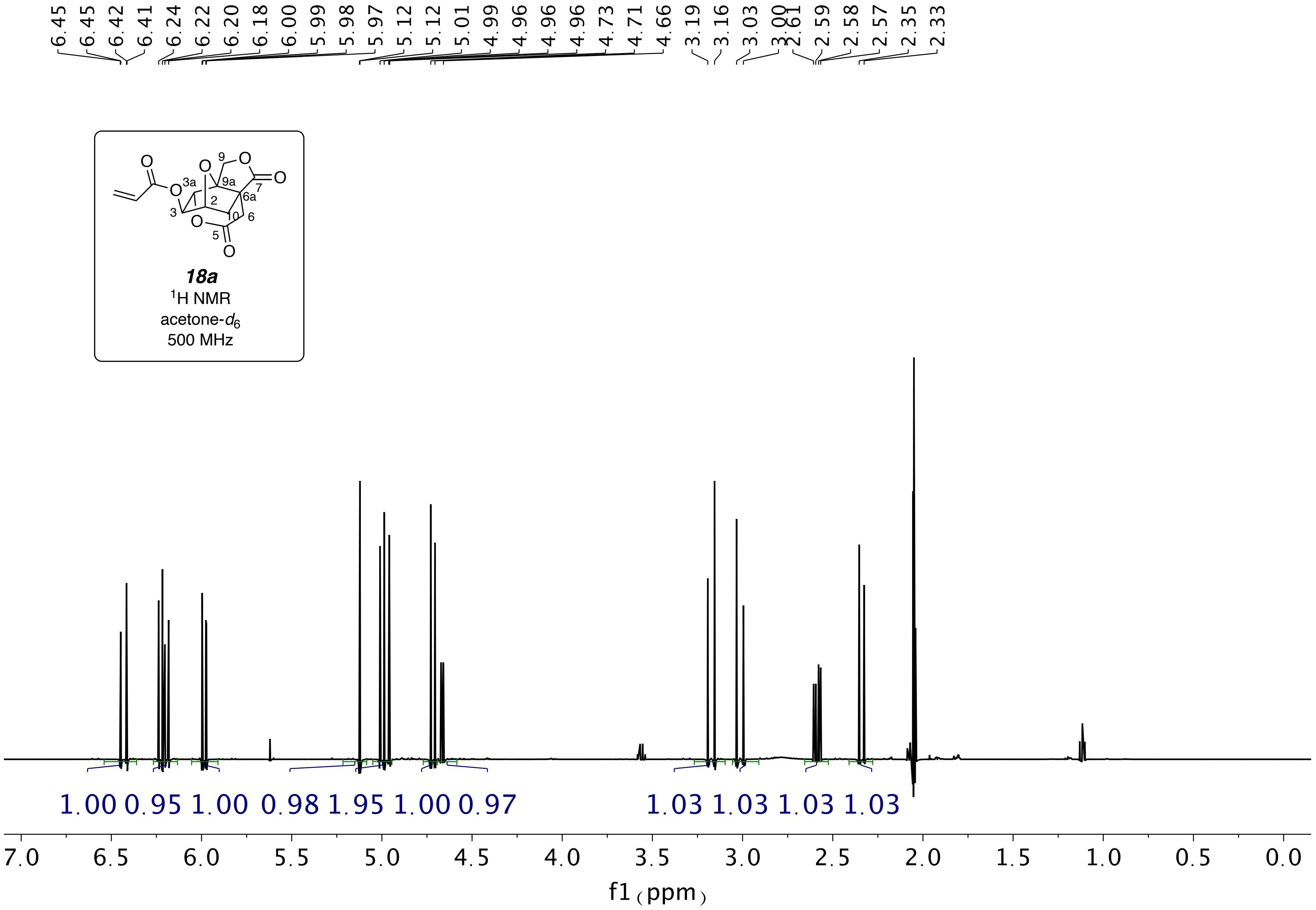
6.45 6.45 6.42 6.41 6.24 6.22 6.20 6.18 6.00 5.99 5.98 5.97 5.12 5.12 5.01 4.99 4.96 4.96 4.96 4.73 4.71 4.66 3.19 3.16 3.03 3.00 2.61 2.59 2.58 2.57 2.35 2.33
18a
^{1}H NMR
acetone-d_6
500 MHz
1.00 0.95 1.00 0.98 1.95 1.00 0.97 1.03 1.03 1.03 1.03
7.0 6.5 6.0 5.5 5.0 4.5 4.0 3.5 3.0 2.5 2.0 1.5 1.0 0.5 0.0
f1 (ppm)

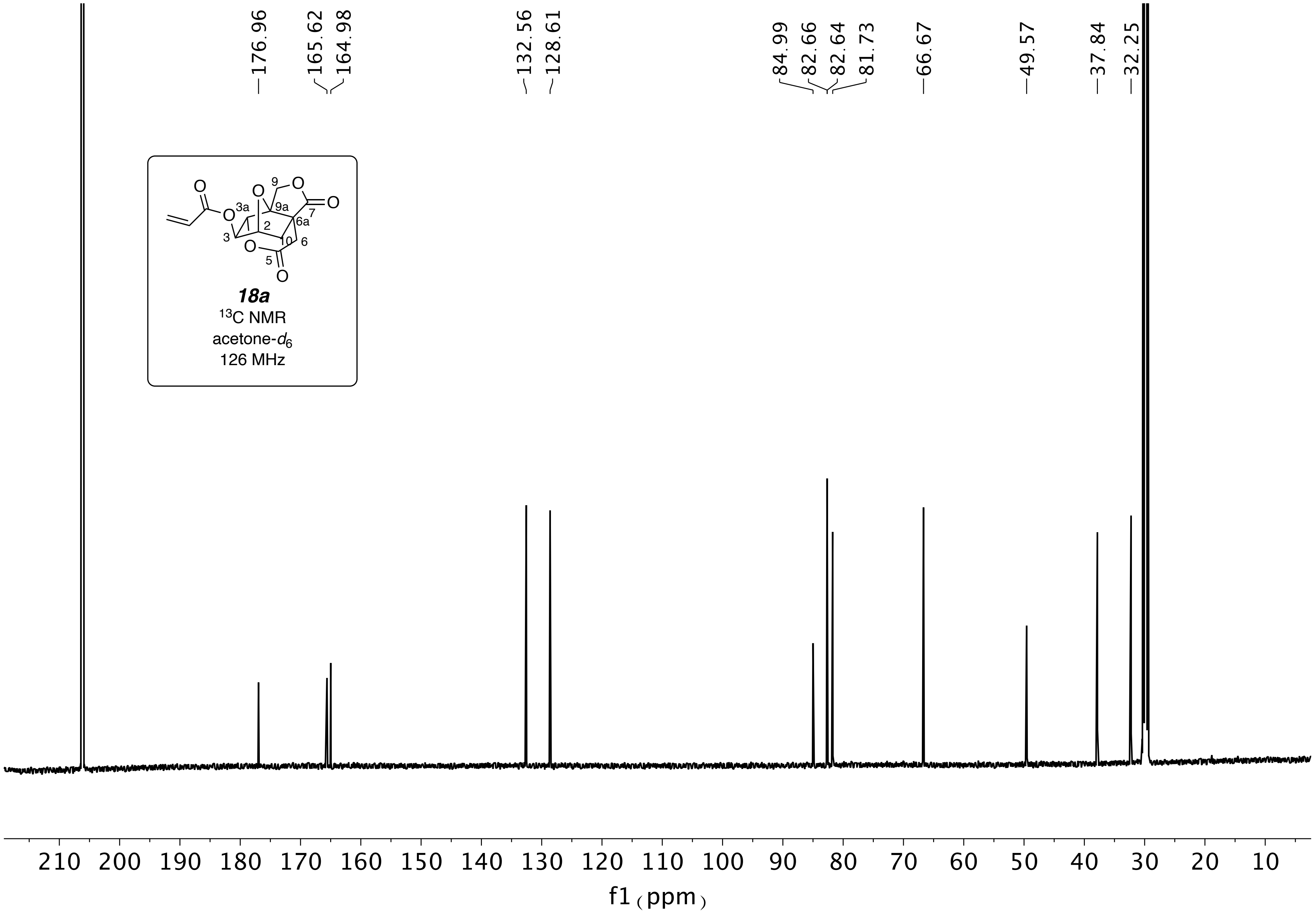
176.96
165.62
164.98
132.56
128.61
84.99
82.66
82.64
81.73
66.67
49.57
37.84
32.25
O
O
9
O
3a
9a
O
7
6a
2
3
10
6
O
5
O
18a
^{13}C NMR
acetone-d_6
126 MHz
210 200 190 180 170 160 150 140 130 120 110 100 90 80 70 60 50 40 30 20 10
f1 (ppm)

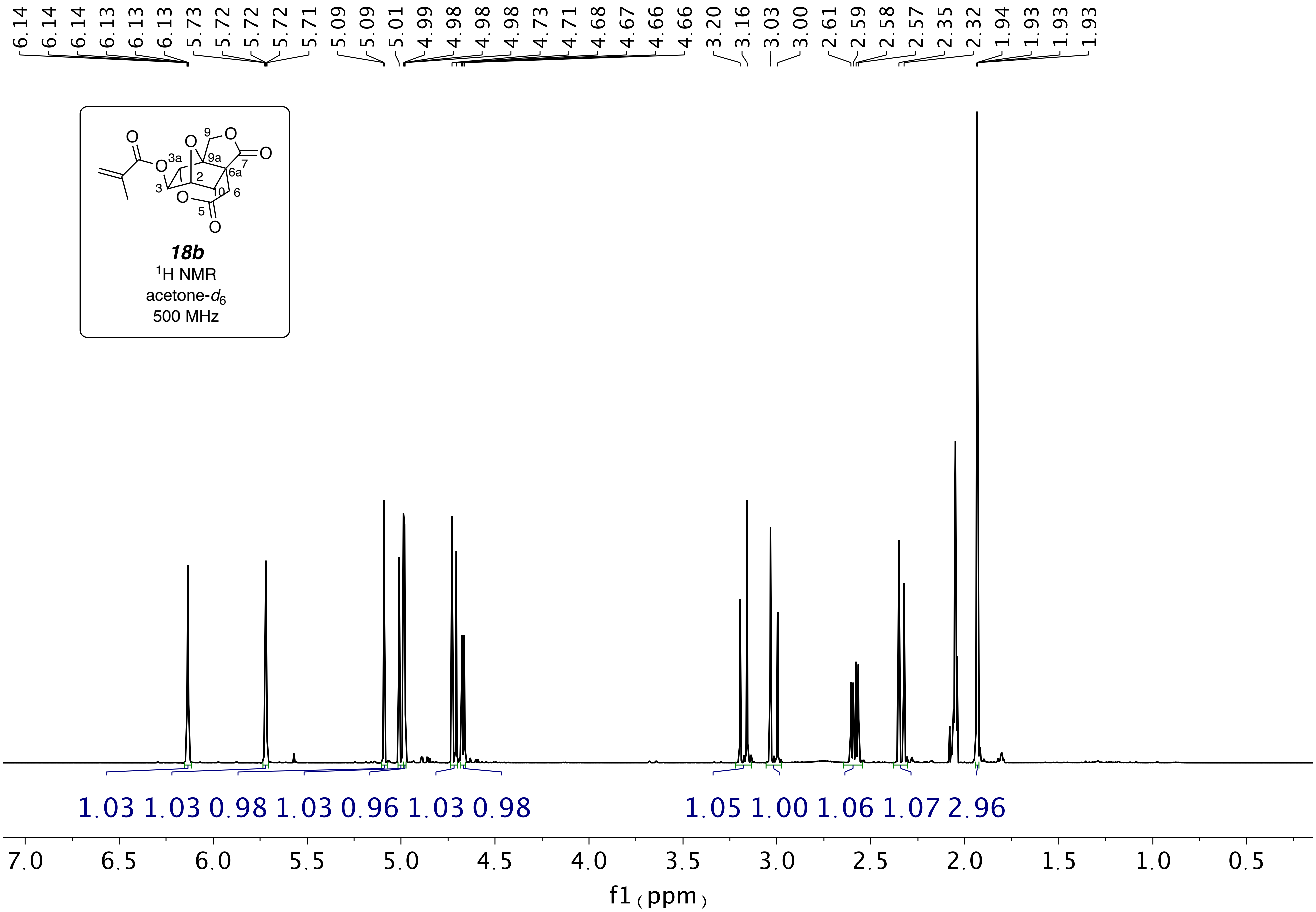
6.14
6.14
6.14
6.13
6.13
6.13
5.73
5.72
5.72
5.72
5.71
5.09
5.09
5.01
4.99
4.98
4.98
4.98
4.73
4.71
4.68
4.67
4.66
4.66
3.20
3.16
3.03
3.00
2.61
2.59
2.58
2.57
2.35
2.32
1.94
1.93
1.93
1.93
18b
1H NMR
acetone-d6
500 MHz
1.03 1.03 0.98 1.03 0.96 1.03 0.98
1.05 1.00 1.06 1.07 2.96
7.0 6.5 6.0 5.5 5.0 4.5 4.0 3.5 3.0 2.5 2.0 1.5 1.0 0.5
f1 (ppm)

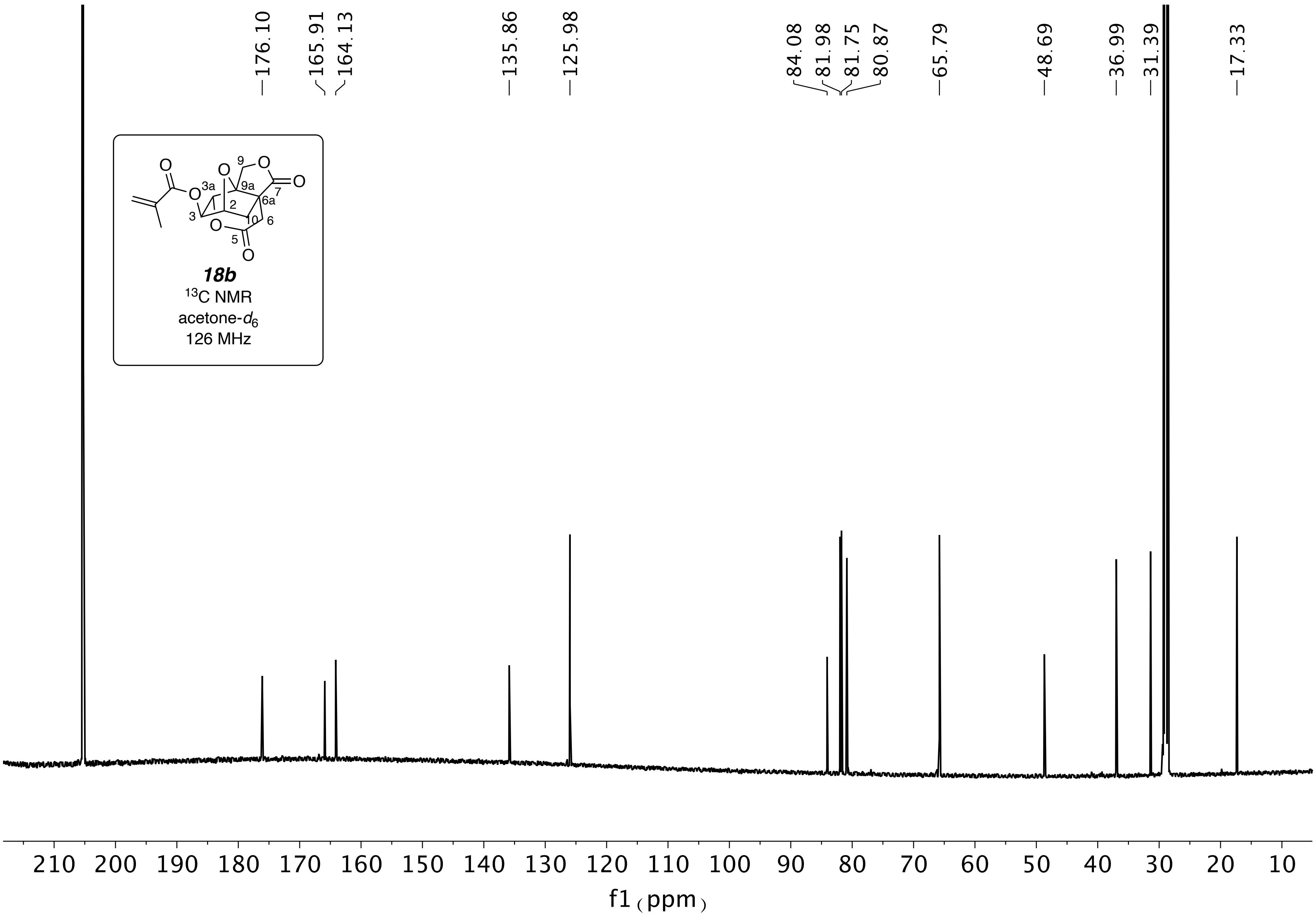
176.10
165.91
164.13
135.86
125.98
84.08
81.98
81.75
80.87
65.79
48.69
36.99
31.39
17.33
18b
13C NMR
acetone-d6
126 MHz
210 200 190 180 170 160 150 140 130 120 110 100 90 80 70 60 50 40 30 20 10
f1 (ppm)

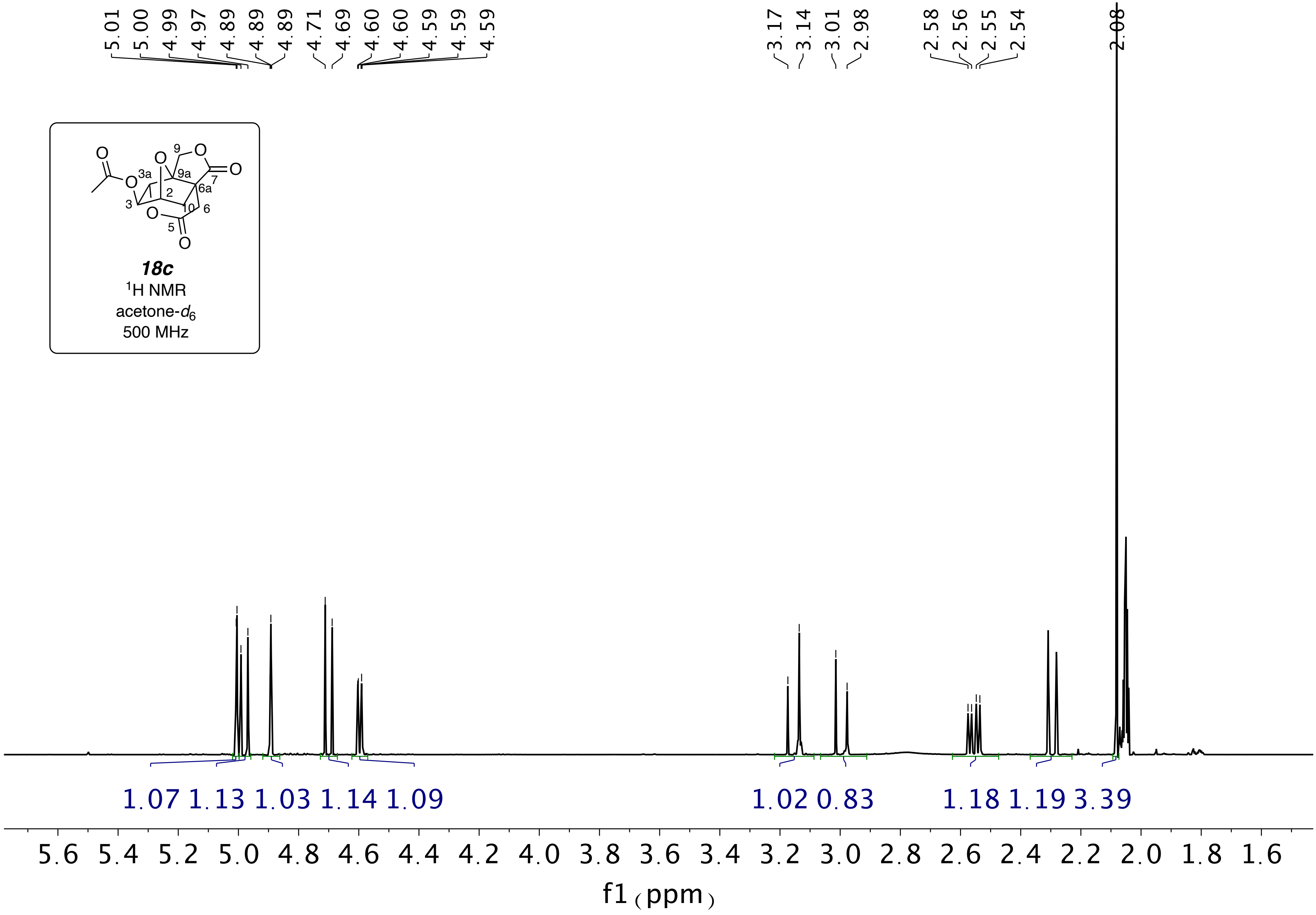
5.01 5.00 4.99 4.97 4.89 4.89 4.89 4.71 4.69 4.60 4.60 4.59 4.59 4.59
3.17 3.14 3.01 2.98
2.58 2.56 2.55 2.54
2.08
18c
1H NMR
acetone-d6
500 MHz
1.07 1.13 1.03 1.14 1.09
1.02 0.83
1.18 1.19 3.39
5.6 5.4 5.2 5.0 4.8 4.6 4.4 4.2 4.0 3.8 3.6 3.4 3.2 3.0 2.8 2.6 2.4 2.2 2.0 1.8 1.6
f1 (ppm)

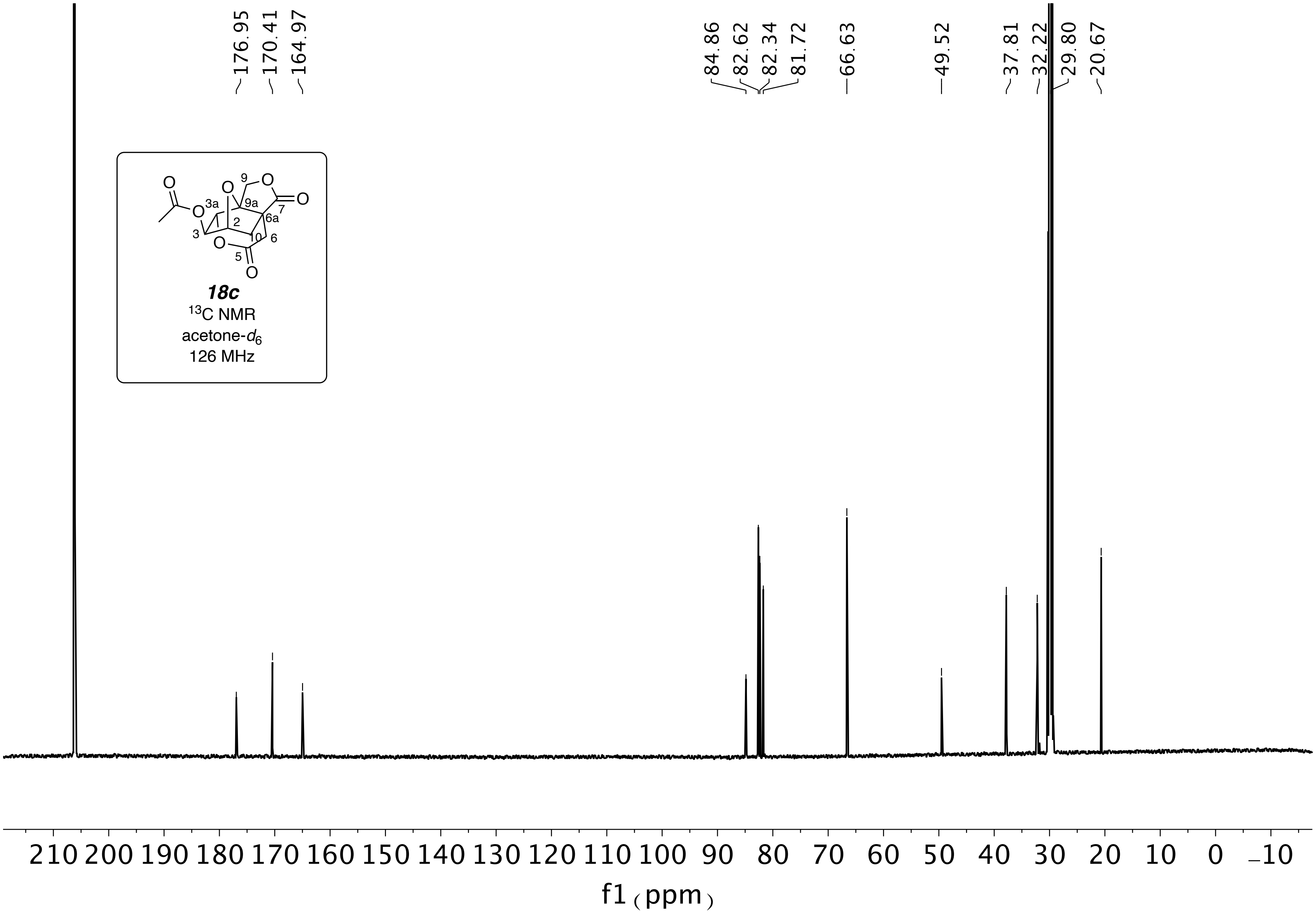
176.95
170.41
164.97
84.86
82.62
82.34
81.72
66.63
49.52
37.81
32.22
29.80
20.67
18c
^{13}C NMR
acetone-d_6
126 MHz
210 200 190 180 170 160 150 140 130 120 110 100 90 80 70 60 50 40 30 20 10 0 –10
f1 (ppm)

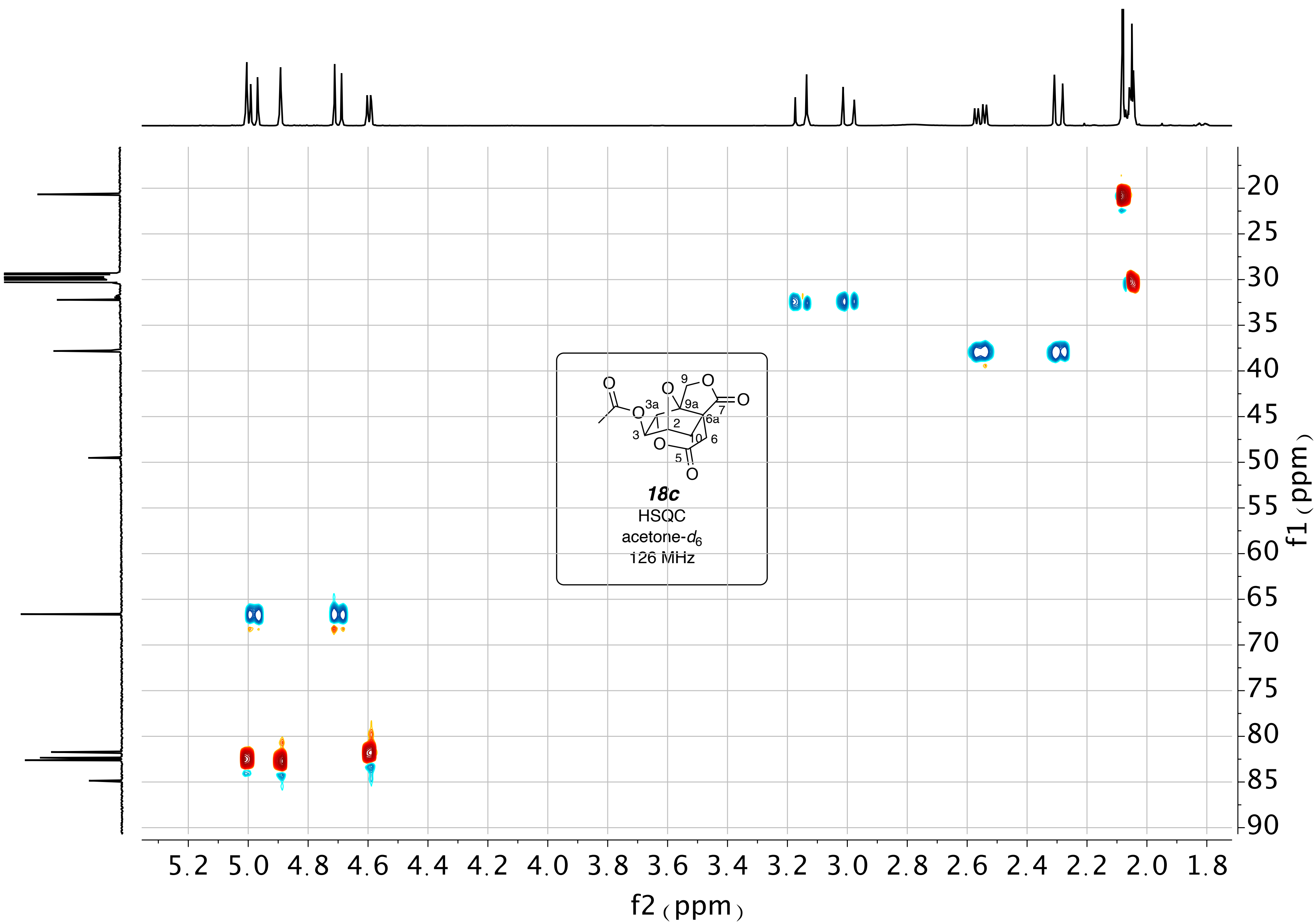

18c
HSQC
acetone-d_6
126 MHz
f2 (ppm)
f1 (ppm)
5.2 5.0 4.8 4.6 4.4 4.2 4.0 3.8 3.6 3.4 3.2 3.0 2.8 2.6 2.4 2.2 2.0 1.8
20 25 30 35 40 45 50 55 60 65 70 75 80 85 90

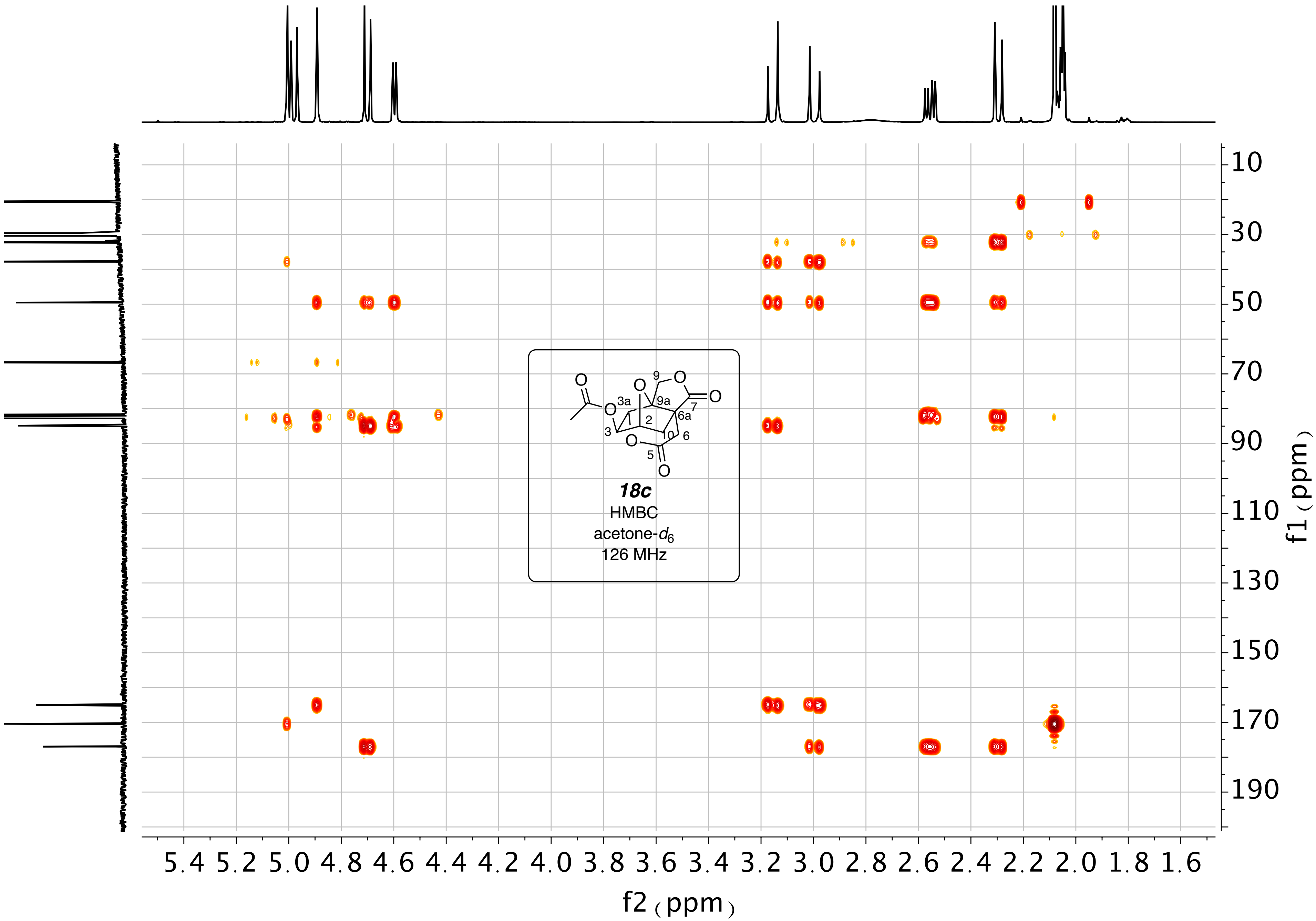
18c
HMBC
acetone-d_6
126 MHz
f2 (ppm)
f1 (ppm)

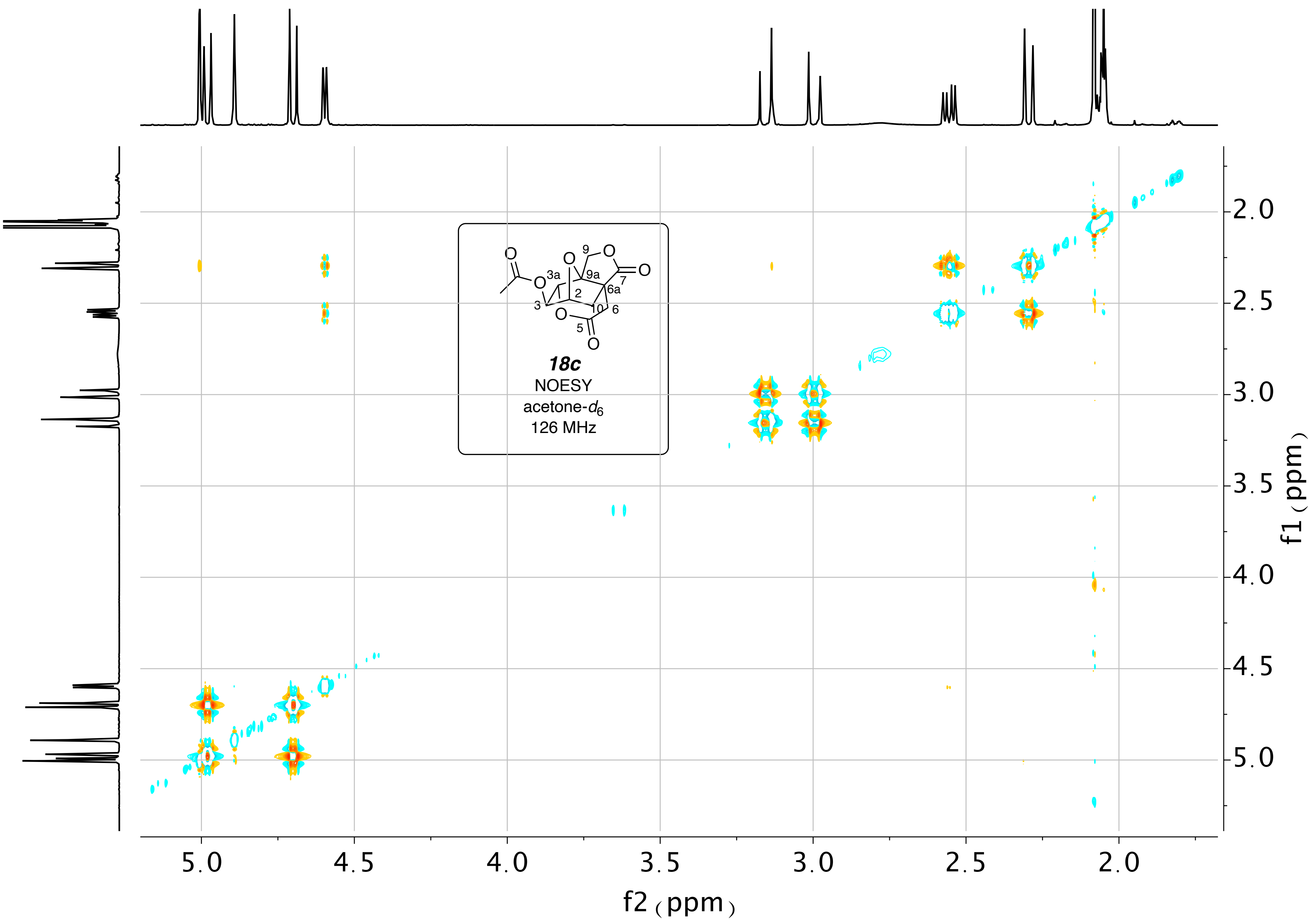
18c
NOESY
acetone-d_6
126 MHz
f2 (ppm)
f1 (ppm)
